# Near-Ambient-Temperature Dehydrogenative Synthesis of the Amide Bond: Mechanistic Insight and Applications

**DOI:** 10.1021/acscatal.1c00728

**Published:** 2021-06-07

**Authors:** Sayan Kar, Yinjun Xie, Quan Quan Zhou, Yael Diskin-Posner, Yehoshoa Ben-David, David Milstein

**Affiliations:** † Department of Molecular Chemistry and Materials Science, The Weizmann Institute of Science, Rehovot 76100, Israel; ‡ Department of Chemical Research Support, The Weizmann Institute of Science, Rehovot 76100, Israel

**Keywords:** amides, dehydrogenation, ruthenium pincer, pharmaceuticals, alcohol, amine

## Abstract

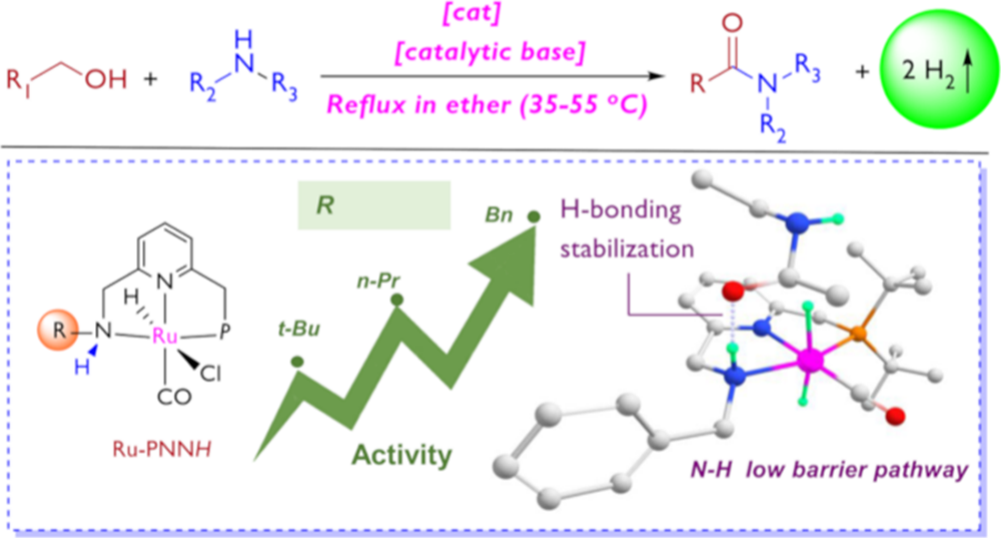

The current existing methods for the amide bond synthesis *via* acceptorless dehydrogenative coupling of amines and alcohols all require high reaction temperatures for effective catalysis, typically involving reflux in toluene, limiting their potential practical applications. Herein, we report a system for this reaction that proceeds under mild conditions (reflux in diethyl ether, boiling point 34.6 °C) using ruthenium PNNH complexes. The low-temperature activity stems from the ability of Ru–PNNH complexes to activate alcohol and hemiaminals at near-ambient temperatures through the assistance of the terminal N–H proton. Mechanistic studies reveal the presence of an unexpected aldehyde-bound ruthenium species during the reaction, which is also the catalytic resting state. We further utilize the low-temperature activity to synthesize several simple amide bond-containing commercially available pharmaceutical drugs from the corresponding amines and alcohols *via* the dehydrogenative coupling method.

## Introduction

The amide group is one of the most ubiquitous functional groups found in Nature. The formation of the amide bond is a fundamental reaction with important utility in the synthesis of natural compounds, biologically active pharmaceutical drugs, short-chain peptides, industrial chemicals, and polymers such as nylons as well as in devising liquid organic hydrogen carrier systems.^1^ Roughly 25% of the currently approved pharmaceutical drugs contain amide groups.^2^ The traditional method of facile amide bond synthesis has been the coupling of reactive acid derivatives, such as acid chlorides or anhydrides, with amines, but these methods are poorly tolerated by other nucleophilic functional groups and generate waste. Other methods involve direct coupling of carboxylic acids and amines under milder conditions in the presence of coupling promoters, which also generate significant amounts of waste.^3^ In 2005, ACS GCI Pharmaceutical Roundtable reported the “amide bond formation avoiding poor atom economy” as one of the most preferred reactions to develop.^4^

**Figure 1:**
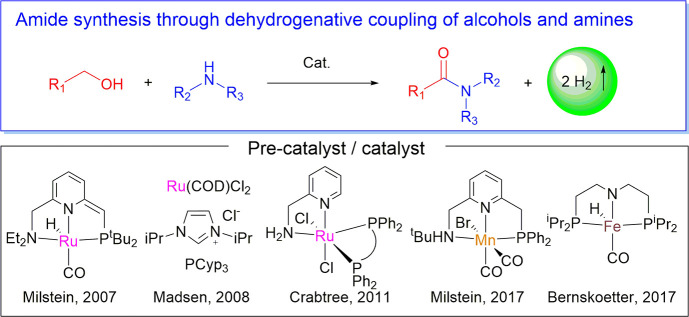
Selected examples of amide synthesis *via* dehydrogenative coupling of amines and alcohols.

In 2007, our group reported a new, environmentally benign synthesis of the amide bond by acceptorless dehydrogenative coupling of alcohols and amines with H_2_ being the sole byproduct of the reaction (Figure 1).^5^ The reaction proceeds upon refluxing the alcohol and amine in toluene in the presence of a Ru–PNN catalyst. Since then, many research groups including the groups of Madsen, Crabtree, Hong, Hazari and Bernskoetter, Prakash, and others have reported efficient catalysts that can catalyze the acceptorless dehydrogenative coupling of alcohols and amines under various conditions.^6^ Importantly, first-row transition-metal-based complexes have also been reported for this transformation by the groups of Bernskoetter^6a^ (iron) and ours^7^ (manganese). Some heterogeneous catalysts are also known to catalyze this transformation.^8^

All the systems mentioned above require a reaction temperature that is generally above 100 °C (typically reflux in toluene), which decreases their practical utility. One of the primary advantages of homogeneous catalysis by well-defined molecular complexes over heterogeneous catalysts is that the activity of the homogeneous complexes can be rationally improved *via* judicious tuning of the ligand framework. The high reaction temperature required for dehydrogenative amide bond formation is presumably due to the difficulty in the hydride abstraction from the reactant and the hemiaminal intermediate by the employed catalysts, en route to the dihydride intermediate. Wang and co-workers had computationally investigated the mechanism of this amide formation in detail with the traditional Ru–PNN_Et_ Milstein complex, and according to their calculations, hydride abstraction from the alcohol and hemiaminal steps have activation barriers of around 25 and 31 kcal/mol, respectively.^9^ The high energy requirement, especially for the hemiaminal dehydrogenation, is in line with the elevated temperature required for the amide synthesis. Moreover, the overall formation of amide and H_2_ from alcohol and amine is generally thermodynamically uphill, also contributing toward the high required temperature for amide synthesis. In this context, development of lower-temperature dehydrogenative amide synthesis protocols by rational catalyst design is desirable in order to improve the applicability of this atom-economical dehydrogenative coupling process.

## Results and Discussion

These considerations led us to explore the possibility of a low-temperature amide synthesis pathway *via* the acceptorless dehydrogenative coupling.^10^ We started our investigation with the synthesis of *N*-heptylhexanamide by coupling of 1-hexanol and 1-heptylamine (Table 1). Several ruthenium and manganese pincer complexes were screened for the amide synthesis under Et_2_O reflux (boiling point 34.6 °C) in the presence of catalytic amounts of *t*-BuOK. Interestingly, among these complexes, Ru–PNNH complexes^11^**1** and **2**, featuring a terminal N–H moiety, displayed catalytic activities toward amide formation even at this low temperature (entries 1–2). Ru–PNNH complexes **1–3** are capable of two distinct modes of metal–ligand cooperation (MLC), amido–amine and aromatization–dearomatization,^11^ unlike complexes **4–6**, where only aromatization–dearomatization is possible. Ru–P^Ph^NNH complex **3**, with electron-withdrawing Ph substituents on the P donor atom, did not show catalytic activity at this temperature (entry 3). Comparing **1** and **2**, the *N*-benzyl-substituted PNNH complex **2** showed higher activity than the *tert*-Bu-substituted complex **1**. The reason behind the higher activity of complex **2** is investigated in more detail during mechanistic investigations (*vide infra*).

**Table 1:**
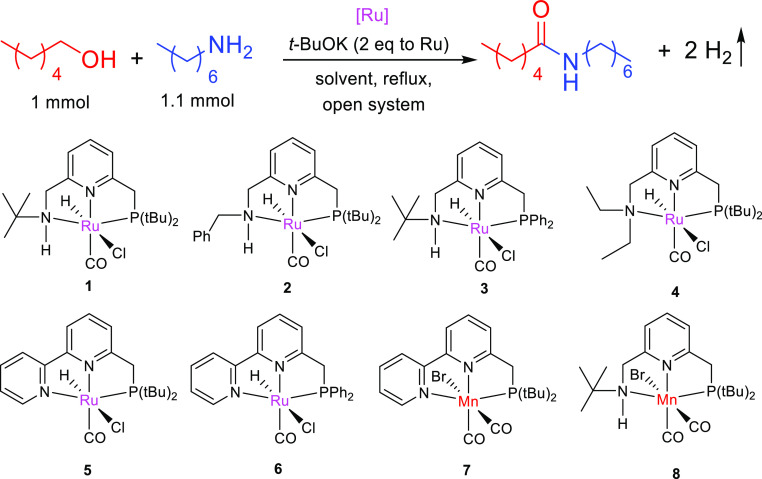
Conditions Screening for Low-Temperature Synthesis of *N*-Heptylhexanamide^a^

entry	[Ru]	solvent (b.p./°C)	time (h)	amide (%)^b^
1	1	Et_2_O (34.6)	12	11
2	2	Et_2_O	12	66
3	3	Et_2_O	12	0
4	4	Et_2_O	12	0
5	5	Et_2_O	12	1
6	6	Et_2_O	12	0
7	7	Et_2_O	12	0
8	8	Et_2_O	12	0
9^d^	2	Et_2_O	22	92
10	2	MTBE (55.2)	12	97 (95)^c^

a Reaction conditions: 1-hexanol (1 mmol), 1-heptylamine (1.1 mmol), [Ru] (0.01 mmol), *t*-BuOK (0.02 mmol), solvent (2 mL), reflux with bath temperatures of 50 and 70 °C for Et_2_O and MTBE, respectively, time as specified.

b Yields are calculated based on ^1^H NMR spectra with mesitylene as an internal standard.

c Isolated yield.

d Reaction scaled down by a factor of 2 (solvent 2 mL).

Traditional PNN complexes, **4–6** (entries 4–6), with only one mode of MLC (aromatization–dearomatization) were not active at low temperature, although they can catalyze the reaction at the higher temperature of 110 °C (entries 4–8).^5,6b^ Thus, the terminal N–*H* moiety of the Ru–PNNH complexes likely plays an important role in their low-temperature catalytic activity. Mn-based PNN and PNNH complexes were also not active at this low temperature (entries 7–8). High conversion of 1-hexanol and 1-heptylamine to the corresponding amide was obtained with **2** as a pre-catalyst and diethyl ether as a solvent after 22 h of reflux (entry 9). A faster reaction was observed at a higher temperature such as reflux in the methyl *tert*-butyl ether (MTBE) solvent (b.p.: 55.2 °C) (entry 10). The generation of H_2_ gas was confirmed by carrying out the reaction in a closed vessel and analyzing the headspace gas by gas chromatography after the reaction (Figure S5).

The substrate scope of this low-temperature amide synthesis system was subsequently explored (Table 2). Simple amides such as *N*-heptylhexanamide, *N*-benzylhexanamide, *N*-heptylbenzamide, *N*-benzylbenzamide, and *N*-(furfuryl)hexanamide were synthesized in high yields under refluxing conditions in diethyl ether (Table 2, entries 1–6). Besides primary amines, the secondary amine morpholine and 1-hexanol can also couple at low temperature in the presence of **2**, producing the tertiary amide *N*-hexanoylmorpholine in excellent yield (entry 7). Different halogen substituents such as −F and −Br are also tolerated under the reaction conditions (entries 8–9). For the synthesis of some other amides, such as *N*-heptyl-2-methoxyacetamide and *N*-heptylisovaleramide, a slightly higher temperature was required for complete conversions (reflux in MTBE) (entries 10–13).^5^ Highly reducible groups, such as a C–C double bond, are also tolerated under the conditions despite the reaction being associated with the evolution of H_2_ gas (entry 14). It is to be noted that the nucleophilicity of the amine plays an important role in the rate of the reactions. For example, in the case of the synthesis of *N*-phenylhexanamide, the low nucleophilicity of aniline necessitates either a higher reaction temperature (reflux in toluene) or an increased base concentration (50 mol % KOtBu) for effective amidation (entries 15–17). Notably, ethylenediamine and ethanol can also dehydrogenatively couple at low temperatures (reflux in MTBE) in the presence of complex **2** to provide the diamide in 95% yield (entry 18). We also investigated the possibility of the synthesis of chiral amides *via* this method using a β-chiral alcohol and an α-chiral amine. The chiral centers of the substrates were largely retained in the product amide molecules in both cases, as determined from optical rotation analysis of the products in comparison with literature data, demonstrating the potential utility of this method in the synthesis of biologically active amide molecules (entries 19–20, Figures S41 and S42).

**Table 2:**
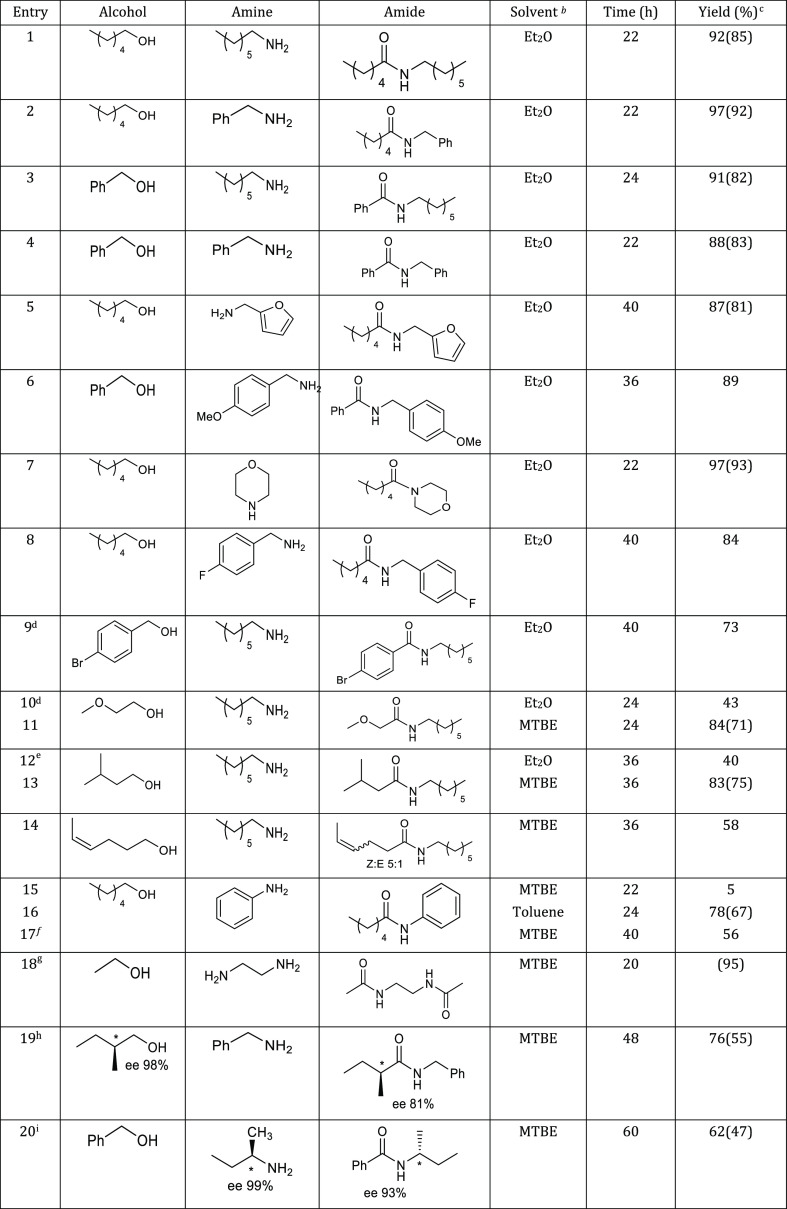
Dehydrogenative Synthesis of Various Amides at Low Temperature^a^

a Reaction conditions: alcohol (0.5 mmol), amine (0.55 mmol), **2** (0.005 mmol), *t*-BuOK (0.01 mmol), solvent (2 mL), reflux under Ar in an open system [the bath temperature was 50 °C (Et_2_O) or 70 °C (MTBE) or 130 °C (toluene)].

b b.p. under 1 atmospheric pressure.

c Yields calculated similar to those in Table 1; in parentheses are isolated yields.

d Rest of the alcohol unchanged.

e Ester 10% observed, rest 50% of alcohol unchanged.

f 50 mol % KO^*t*^Bu.

g Ethylenediamine (0.5 mmol), EtOH (1.5 mmol), **1** (0.005 mmol), and *t*-BuOK (0.01 mmol).

h Cat (2 mol %), in a 100 mL closed flask.

i Cat (2 mol %), in a 25 mL closed flask; the generated gas inside released intermittently after cooling down (24th and 48th h).

**Scheme 1:**
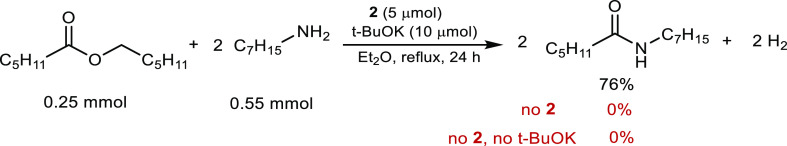
Amide Formation from Ester Catalyzed by **2**

In some of the reactions of Table 2 (entries 4, 5, 8, and 14), esters in moderate amounts were detected (∼0–10%). To understand whether the generated ester is converted to the amide at this low temperature or is exclusively a competing reaction pathway, we set up a reaction of hexyl hexanoate with 1-heptylamine. After 24 h of refluxing in Et_2_O under similar reaction conditions, the formation of the corresponding amide, *N*-heptylhexanamide, in 76% yield was observed (Scheme 1), signifying that complex **2** can also catalyze the synthesis of amides from esters at low temperatures, presumably *via* initial nucleophilic substitution of the ester with the amine to form an amide and an alcohol, followed by dehydrogenative coupling between the released alcohol and amine to form amide.^12^ In the absence of the ruthenium complex, no conversion of ester to amide was observed, signifying that the ruthenium complex catalyzes this reaction, likely acting as a Lewis acid to activate the ester during the initial amine nucleophilic attack on the ester.

**Scheme 2:**
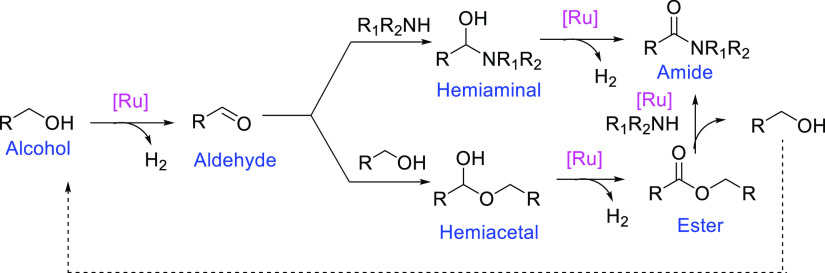
Pathway of Amide Formation

Based on these observations, the reaction pathway for the amide formation is shown in Scheme 2. The initial dehydrogenation of alcohol forms the aldehyde, which further converts to hemiaminal or hemiacetal *via* nucleophilic attack of the amine or alcohol, respectively. Subsequent dehydrogenation of the hemiaminal and hemiacetal intermediates produces the amide and ester, respectively. The ester is further converted to amide *via* nucleophilic substitution by the amine, assisted by the ruthenium complex under the reaction conditions.

**Scheme 3:**
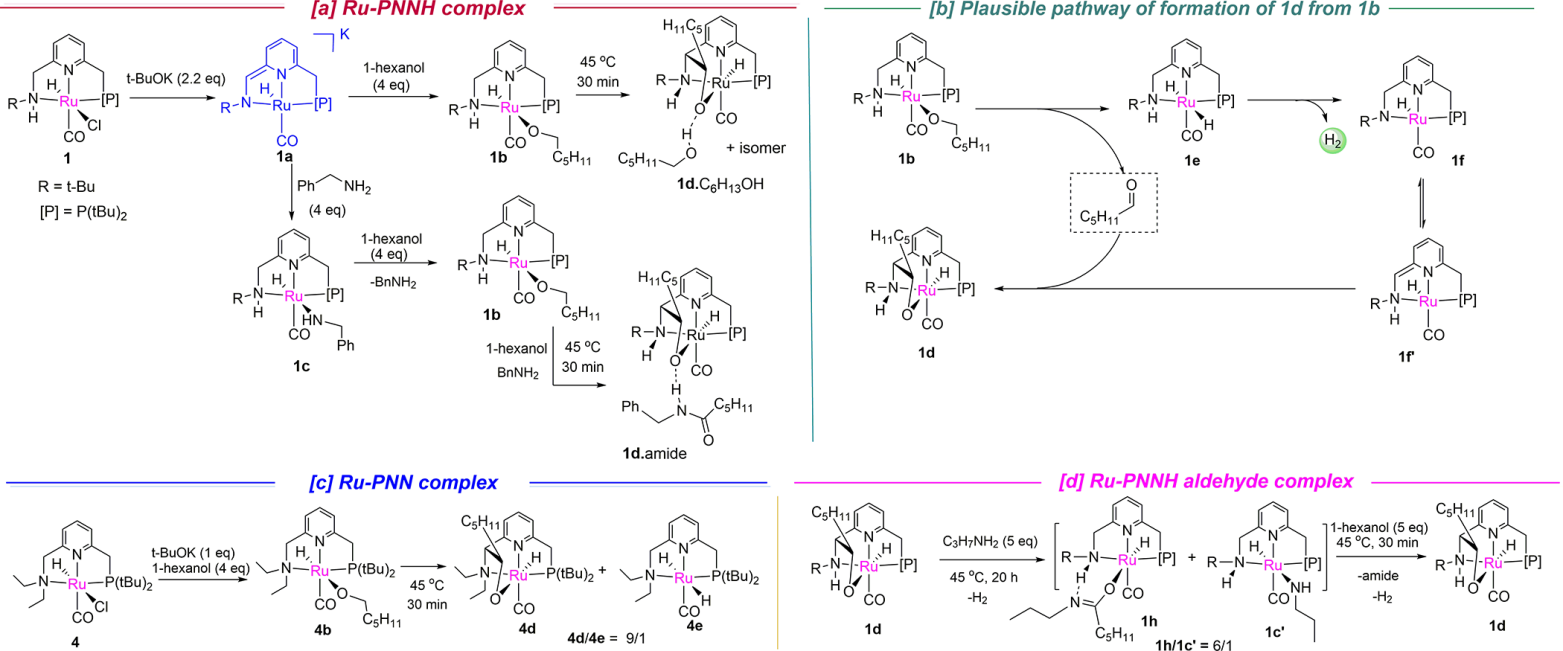
Reactivity of Ru Complexes with the Base, Alcohol, and Amine

Mechanistic studies were carried out to further understand the mechanism of the low-temperature catalytic activities of the PNNH complexes (Scheme 3). Complex **1**, upon addition of 2 eq of *t*-BuOK, forms the anionic complex **1a** which is intensely violet in diethyl ether solution (Scheme 3a).^11^ Addition of 1-hexanol (4 equiv) to this complex results in the formation of the aromatic alkoxy complex **1b** (see Figure S20 for reaction progress). Noteworthily, the alkoxy ligand of **1b** exchanges quickly with the free alcohol in solution, and as the excess alcohol is removed from the solution, peak broadening in ^31^P{^1^H} and ^1^H NMR is observed.^13^ Similar to the formation of the alkoxy complex, addition of benzylamine(4 equiv) to a solution of **1a** resulted in the formation of the amido complex **1c**. Further addition of alcohol to the amido complex replaced the amido ligand to form the alkoxy complex along with the generation of amine (Scheme 3a; Figure S21). On a similar note, addition of a 1/1 alcohol and amine solution to complex **1a** resulted in the selective formation of the alkoxy complex in solution.

**Figure 2:**
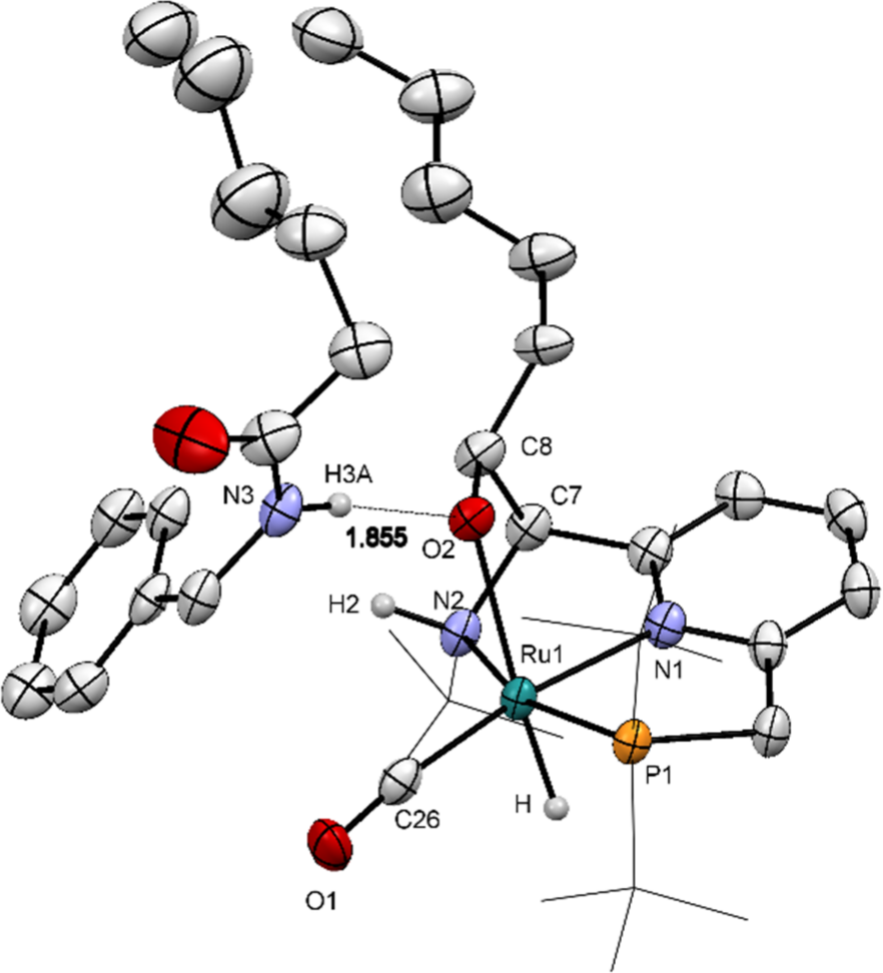
ORTEP diagram of complex **1d**.amide. Atoms are drawn with a probability level of 50%. Selected hydrogen atoms omitted for clarity. *Tert*-butyl groups displayed as a wireframe for clarity. Selected bond lengths (Å) and angles (^o^): Ru(1)–P(1) 2.2576(5), Ru(1)–O(2) 2.2380(15), Ru(1)–N(1) 2.0850(19), Ru(1)–N(2) 2.2010(17), Ru(1)–C(26) 1.818(2), O(2)–C(8) 1.394(3), C(7)–C(8) 1.570(3); P(1)–Ru(1)–H 81.5(11), O(2)–Ru(1)–P(1) 105.13(4), O(2)–Ru(1)–H 168.8(11), N(1)–Ru(1)–P(1) 81.81(5), N(1)–Ru(1)–O(2) 81.74(7), N(2)–Ru(1)–O(2) 72.92(6), C(8)–O(2)–Ru(1) 113.36(13), O(2)–C(8)–C(7) 109.38(18).

When the resulting solution containing complex **1b**, amine, and alcohol was heated at 45 °C in a *J*. Young NMR tube, the formation of a new complex was observed after 30 min with almost quantitative conversion of **1b** (Scheme 3a; Figure S21). This complex exhibits a characteristic peak in the ^31^P NMR at 119.4 ppm (major isomer) (Figure S14).^14^ In the proton NMR, a hydride peak corresponding to this complex was observed at −15.2 ppm as a doublet (*J* = 28.5 Hz) (Figure S8). In the IR spectrum, a strong absorption band at 1900 cm^–1^ was observed, corresponding to a CO ligand. Interestingly, the ^13^C NMR spectrum indicated the activation of the *N*-arm as the secondary picolylic CH_2_ unit of the *N*-arm was converted to a tertiary CH unit along with another CH unit, presumably from an alcohol derivative (Figure S11). Based on 1D and 2D NMR analysis, the structure of **1d** was assigned to a new complex where an *in situ* generated aldehyde binds to the N-arm of the ligand through MLC. Single crystals suitable for X-ray diffraction (XRD) analysis were grown by slow evaporation from a THF/pentane solution of **1d**, and the XRD analysis confirmed the assigned structure of **1d** (Scheme 3a, Figure 2). Interestingly, in the unit cell of the crystal, a product amide molecule of *N*-benzylhexanamide was found attached to **1d**, forming **1d**.amide *via* hydrogen bonding (Figure 2). The aldehyde addition across the metal and ligand arm has been documented before by us with a Ru–PNP system at much lower temperatures (−78 °C)^13^ and also by Sanford and co-workers with the traditional Ru–PNNEt_2_ system at room temperature following the addition of benzaldehyde to the dearomatized complexes.^15^ However, it is quite interesting that this aldehyde complex can also be readily and quantitatively accessed form the alkoxy complex upon brief, mild heating, signifying the possible generation of dearomatized complexes during the reaction.

Complex **1d** can also be accessed upon heating the alkoxy complex **1b** in the presence of alcohol but without amine, forming an alcohol adduct **1d**.C_6_H_13_OH (Scheme 3a), as verified by NMR analysis (Figures S8 and S9). The formation of **1d** from **1b** presumably happens *via* the formation of the dihydride **1e**, followed by the formation of amido complex **1f** with the evolution of H_2_ (Scheme 3b). The amido complex **1f** can further convert to the *N*-arm dearomatized complex **1f′**, to which the addition of aldehyde affords complex **1d** (Scheme 3b). However, the dihydride complex was not observed by NMR, signifying that the H_2_ elimination from the dihydride complex **1e** is facile. A similar aldehyde adduct complex also forms with the traditional Ru–PNNEt_2_ complex **4** at 45 °C in the presence of a base and alcohol, although, in this case, the dihydride complex is also observed in the NMR (Scheme 3c, Figure S22). This signifies that (i) even in the case of Ru–PNNEt_2_, the first alcohol dehydrogenation step can proceed at low temperature, at least, stoichiometrically, and (ii) H_2_ generation from **1e** is more facile than from the dihydride complex **4e**, presumably due to the new mode of MLC *via* the involvement of the terminal N–*H* moiety.

**Scheme 4:**
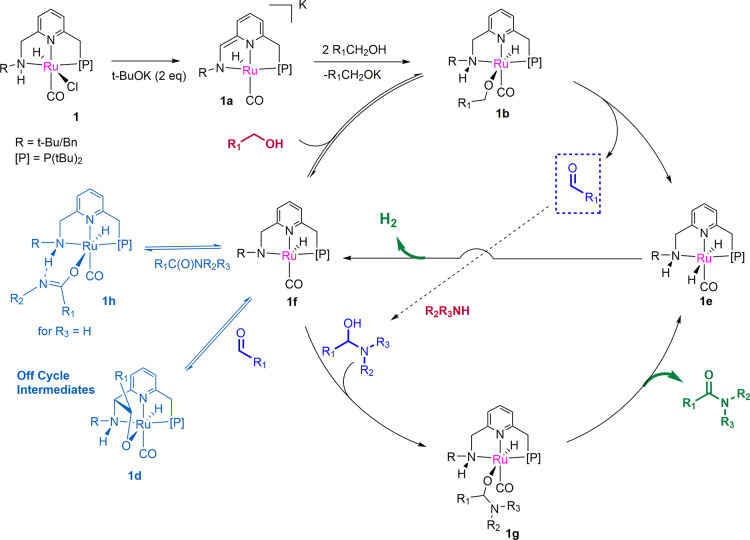
Plausible Mechanistic Cycle

The aldehyde binding to the side arm in complex **1d** is reversible and rapidly exchanges with free benzaldehyde in solution as verified by an aldehyde-exchange experiment (Figure S23).^16^ Complex **1d**, upon addition of *n*-propylamine (5 equiv), followed by mild heating (45 °C), gradually formed a new complex (Scheme 3d) in which the C–C bond was broken as concluded from ^1^H and ^31^P{^1^H} NMR. The transformation is also associated with the formation of H_2_ gas, as expected. Based on the NMR analysis, the new complex is assigned the structure of the amidate complex **1h**, which is formed *via* addition of the *in situ* generated amide product to the amido complex **1f** (Scheme 4). We have previously observed the reversible formation of similar amidate complexes upon addition of amide to the dearomatized PNNH complexes.^17^ The formation of complex **1c′** was also observed in a minor amount resulting from the addition of free amine to complex **1f** (Scheme 3d, Figure S26). The amidate and amido ligands from **1h** and **1c′** are gradually replaced in the presence of 1-hexanol (5 equiv) at 45 °C, regenerating complex **1d** (Scheme 3d, Figure S26). These results clearly demonstrate the competing binding routes of different substrates, intermediates, and products to the PNNH amido complex that are operational during active catalysis (Scheme S1).

**Scheme 5:**
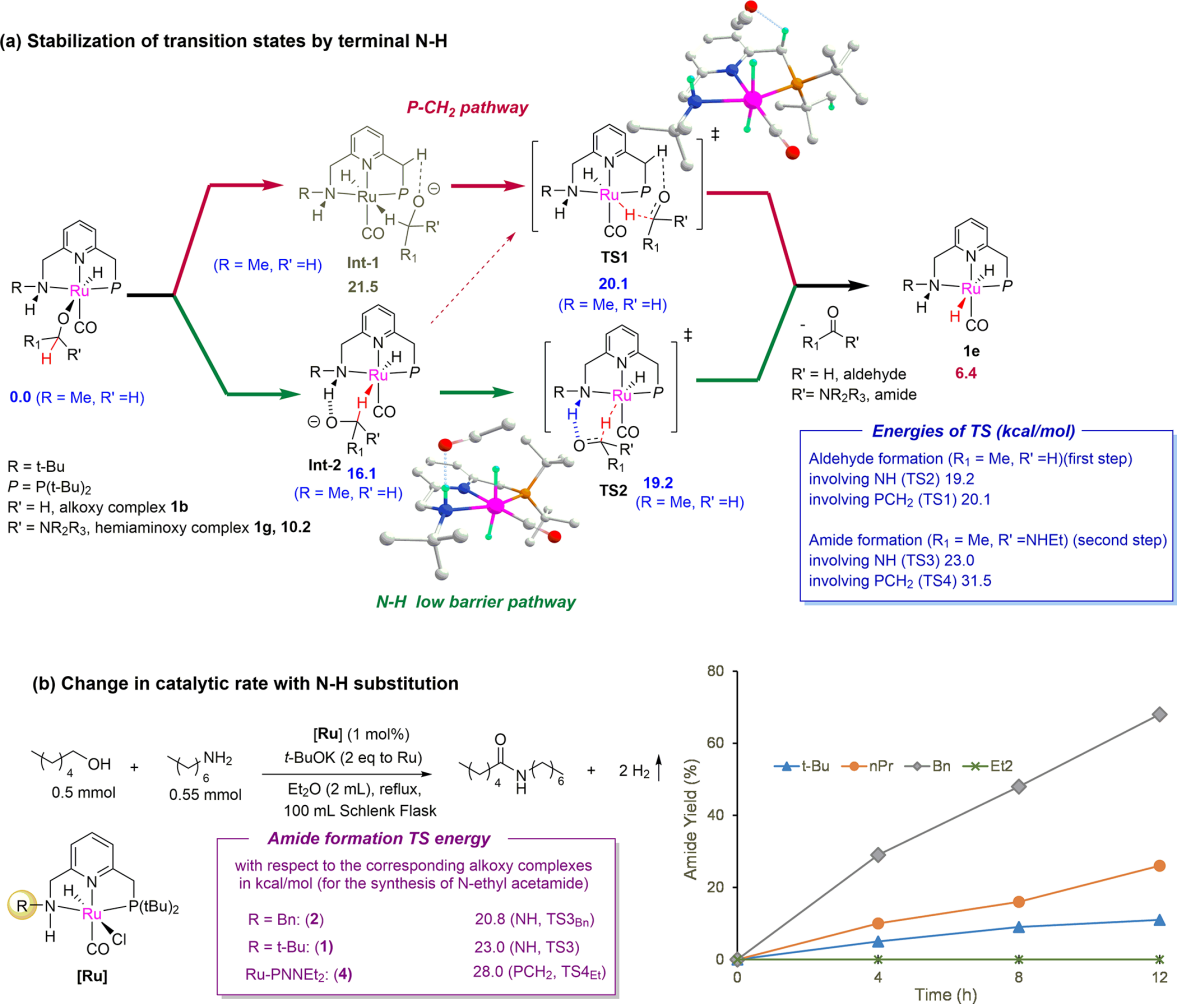
Importance of the Terminal N–H Moiety Energy values correspond to Gibbs free energies (kcal mol^–1^) with respect to the ethoxy complex + ethylamine at 298.15 K in a diethyl ether continuum. All reactant concentrations are 1 M, except for H_2_ which is at 1 atm. Mass balance is ensured throughout.

Based on the mechanistic experiments and on prior investigation by our group and others on the dehydrogenative amide bond formation, we propose a catalytic cycle as depicted in Scheme 4. The pre-catalyst, in the presence of the base and alcohol, forms the alkoxy complex **1b**. Hydride elimination from the alkoxy ligand through an outer-sphere mechanism (Scheme 5a, *vide infra*) forms the dihydride complex **1e**, along with the aldehyde. Subsequently, H_2_ elimination from the dihydride forms the amido complex **1f**. This H_2_ evolution from the dihydride complex can happen by itself or through the assistance of an alcohol molecule. The amido complex then binds to the aldehyde to form the likely off-cycle intermediate complex **1d** (*via* a pathway as depicted in Scheme 3b) which is also the catalytic resting state of the reaction. **1d**, in the presence of an amine, generates the hemiaminoxy complex **1g**, presumably *via* the formation of **1f**. In the subsequent reaction step, hydride elimination from the hemiaminoxy complex generates the amide and the dihydride complex again. Further H_2_ elimination from the dihydride complex **1e**, followed by the addition of alcohol to the resulting amido complex, regenerates the alkoxy complex **1b**, closing the catalytic cycle. It is to be noted that based on our experimental evidence, we cannot exclude the possibility of a beta hydride elimination pathway *via N*-arm opening, although this route is calculated to have higher energy requirement than the stepwise outer-sphere pathway for the traditional Ru–PNNEt_2_ complex.^9^

The hydride abstraction from the alkoxy and the hemiaminoxy complexes, leading to the formation of the aldehyde and amide, respectively, and the dihydride complex, is proposed to proceed *via* the formation of a high-energy intermediate where the hydrogen atom coordinates to the metal center (Scheme 5a, Int 1–2).^9,18^ This step is also associated with a high activation barrier, especially the amide formation step, which has been previously computed to be the highest energy-requiring step in the case of the PNNEt_2_ system.^9^ In the case of the PNNEt_2_ system, this hydride abstraction transition state can proceed *via* the involvement of the picolyl CH_2_ protons. Our computation suggests that in the case of the PNNH system, the terminal N–H moiety can allow the mechanism to proceed *via* a low-energy pathway. For example, the pathway involving the terminal N–H is 0.9 kcal/mol lower in energy for the aldehyde formation step than the PCH_2_ pathway. On the other hand, for the amide formation step, which is also a higher energy-demanding step compared to the aldehyde formation, the new pathway was found to be 8.5 kcal/mol lower in energy (Scheme 5a). This additional stabilization likely explains the high catalytic activities of the PNNH complexes at low reaction temperatures. Please note that although we compute here a stepwise outer-sphere reaction pathway, the possibility of a concerted outer-sphere reaction pathway involving simultaneous proton and hydride transfer from the substrate to the ligand and metal, respectively, similar to the mechanisms associated with many reactions with Noyori and Shvo’s catalysts, cannot be ruled out (Scheme S2).^19^ Nevertheless, the concerted mechanism can also be surmised to be more stabilized by the terminal N–H route as compared to the P–CH_2_ one.

Further support of the importance of the N–*H* proton and its acidity is obtained from comparing the catalysis rate of different Ru–PNNH complexes. As is seen in Table 1 and Scheme 5b, a significant increase in the catalytic activity was observed when the N substitution of the Ru–PNNH complex was changed from *tert*-butyl to the benzyl group. To understand whether this change in activity is likely due to steric or electronic factors, we synthesized the novel *N*-propyl derivative of Ru–PNNH complex **9** (synthesis methods and characterization data are in the Supporting Information). The catalytic activity of the *n*-Pr Ru–PNNH analogue was found to be in between those of the *t*-Bu and Bn analogues under similar reaction conditions (Scheme 5b). This suggests that the higher activity of the benzyl derivative likely results from the electronic properties of the benzyl group since *n*-Pr and the benzyl moiety display similar steric bulks around the N donor. In the case of the benzyl substitution, the terminal N–*H* is more acidic, likely providing the highest stabilization to the transition state of hydride elimination from alkoxy and hemiaminoxy complexes. In addition, based on the density functional theory calculations, an overall lower activation energy was computed for the amide formation in the PNNH_Bn_ system compared to the PNNH_tBu_ system and the PNNEt_2_ system (Scheme 5b). Thus, the acidity of the N–*H* bond is an important factor influencing the low-temperature catalytic activity of these complexes. This also hints at a possible avenue toward developing more efficient catalysts, perhaps functioning under even ambient conditions.

**Scheme 6:**
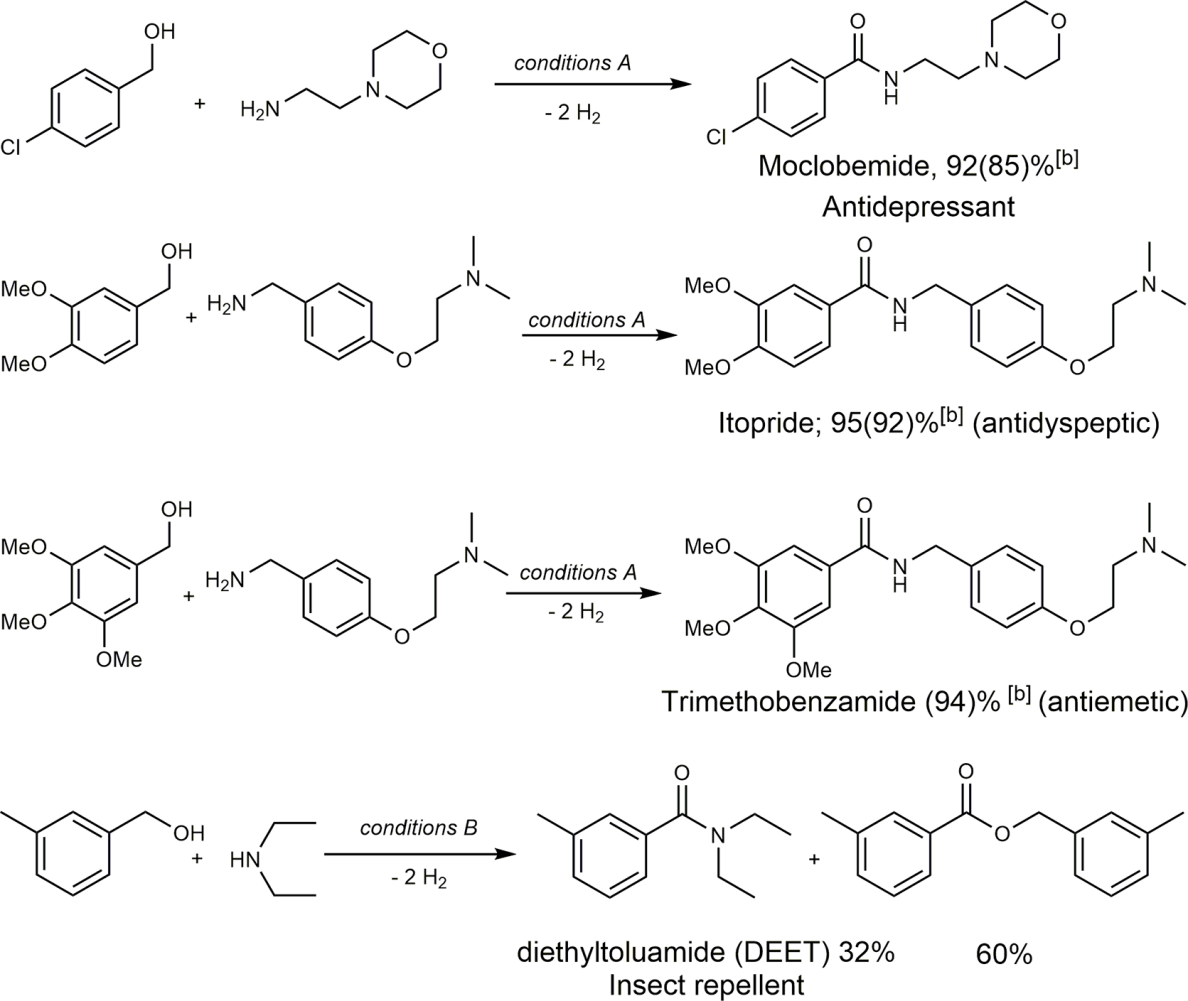
Synthesis of Various Pharmaceutical Drugs Conditions A: alcohol (0.5 mmol), amine (0.6 mmol), **2** (0.005 mmol), *t*-BuOK (0.01 mmol), MTBE (2 mL, bp 55.2 °C), reflux (bath temp. 70 °C), 60 h. Conditions B: alcohol (0.5 mmol), amine (5 mmol), **1** (0.005 mmol), *t*-BuOK (0.01 mmol), K_3_PO_4_ (0.5 mmol), dioxane (2 mL), reflux in a closed tube for 60 h (see the Supporting Information for details). Yields in parentheses are isolated yields, and yields outside parentheses are ^1^H NMR yields.

We also synthesized several commercially available amide bond-containing pharmaceuticals *via* this method at low temperatures to demonstrate its utility (Scheme 6). Despite the activity of several complexes toward catalyzing the dehydrogenative formation of the amide bond, the synthesis of even simple pharmaceutical drugs *via* this atom-economical method has not been demonstrated until now, even at higher reaction temperatures.^20^ Moclobemide is a reversible inhibitor of monoamine oxidase A, approved as an antidepressant in United Kingdom and Canada.^21^ Using the dehydrogenative coupling method, moclobemide was conveniently synthesized from the corresponding alcohol and amine in 92% yield (isolated yield 85%) upon refluxing in MTBE with **1** as a catalyst.^22^ Notably, with H_2_ being the only byproduct of the reaction, the resulting moclobemide can be easily purified by recrystallization in the cold diethyl ether/pentane solvent mixture without the requirement of purification by column chromatography. Similarly, the antidyspeptic drug itopride (brand name: Ganaton)^23^ and the antiemetic trimethobenzamide were synthesized from the corresponding alcohol and amine with 92 and 94% yields, respectively, under similar reaction conditions, without the requirement of purification *via* column chromatography. In the case of the insect repellant diethyltoluamide, it was synthesized in 32% yield *via* this method at higher temperatures (reflux in 1,4-dioxane) and in the presence of 1 eq K_3_PO_4_ due to lower nucleophilicity of the secondary amine compared to the primary ones.^6b^ Further investigation on the synthesis of more complex pharmaceuticals *via* this method through catalyst improvement and condition optimization is ongoing in our group.

## Conclusions

In summary, we report the near-ambient-temperature dehydrogenative formation of the amide bond (reflux in Et_2_O) from alcohols and amines. The Ru–PNNH complexes, featuring a terminal N–*H* unit, showed remarkable catalytic activities at this low temperature. This high activity is presumably due to the new catalytic pathway involving the terminal N–*H* moiety, and the acidity of this terminal N–*H* heavily influenced the low-temperature catalytic rate. An unusual resting state of the complex during catalysis was observed, where the aldehyde binds between the N-side arm and Ru center of the complex *via* a reversible C–C bond formation. Using Ru–PNNH complex **2**, we synthesized several amides under refluxing conditions in Et_2_O or MTBE. Furthermore, several amide bonds containing commercially available pharmaceuticals, including moclobemide, itopride, and trimethobenzamide, were prepared at low temperature *via* this atom-economical method. Our future efforts in this context will be aimed toward the development of even more efficient catalysts for this transformation based on the mechanistic insights gained during this study as well as toward the synthesis of more complex pharmaceuticals *via* this method.

## Supporting Information Available

General information and experimental details (PDF)
Crystallographic data of compounds (CIF)

## Supplementary Material

cs1c00728_si_001.pdf

cs1c00728_si_002.cif

## Notes

Synthetic procedures, NMR spectra, and characterization data for all the new compounds are available within this article and its Supporting Information. The X-ray crystallographic coordinates for the structure reported in this article have been deposited at the Cambridge Crystallographic Data Centre (CCDC) under deposition number CCDC 2043153. The data can be obtained free of charge from The Cambridge Crystallographic Data Centre *via*http://www.ccdc.cam.ac.uk/data_request/cif. Any further relevant data are available from the authors upon reasonable request.
