# Supplementary material for: Near-Ambient-Temperature Dehydrogenative Synthesis of the Amide Bond: Mechanistic Insight and Applications
Source: ACS Catal. 2021 Jun 7;11(12):7383–93. doi: 10.1021/acscatal.1c00728 (PMC8218306; doi:10.1021/acscatal.1c00728)
Supplement: Supplementary file 1 — cs1c00728_si_001.pdf [file cs1c00728_si_001.pdf]

Supporting Information

Near-Ambient Temperature Dehydrogenative Synthesis of the Amide Bond: Mechanistic Insight and Application

Sayan Kar,[a],† Yinjun Xie,[a],†, ‡ Quan Quan Zhou,[a] Yael Diskin-Posner,[b] Yehoshoa Ben-David,[a] and David Milstein*[a]

[a]Department of Molecular Chemistry and Materials Science, [b]Department of Chemical Research Support, The Weizmann Institute of Science, Rehovot 76100, Israel.

Email: david.milstein@weizmann.ac.il
†These authors contributed equally.

‡Present address: Ningbo Institute of Materials Technology & Engineering, Chinese Academy of Sciences, Ningbo, 315201, P.R. China

Contents

1. General Information *S2*
2. ^{1}H and $^{31}P\{^{1}H\}$ NMR spectra of selected complexes *S3*
3. Standard procedure for the dehydrogenative coupling *S5*
4. ^{1}H NMR spectra of selected crude reaction mixtures *S7*
5. Mechanistic reactions *S8*
5.1. Binding of different substrates to the dearomatized/ deprotonated PNNH complexes *S8*
5.2. Synthesis of complex ***1d*** *S9*
5.3. Synthesis of complex ***9*** *S14*
5.4. NMR observations *S18*
6. X ray crystallography *S26*
7. Procedure for the dehydrogenative synthesis of pharmaceuticals *S28*
8. ^{1}H and $^{13}C\{^{1}H\}$ spectra of isolated compounds *S31*
9. Computational details *S46*
10. References *S77*

1. General Information

All dehydrogenation experiments were carried out under an inert atmosphere (with N_2 or Ar) using standard Schlenk techniques. Complexes RuHCl(CO)P^{tBu}NN$_{tBu}$H (**1**),[1] RuHCl(CO)P^{tBu}NN$_{Bn}$H (**2**),[1] RuHCl(CO)P^{Ph}NN$_{tBu}$H (**3**),[2] RuHCl(CO)P^{tBu}NN$_{Et}$ (**4**),[3] RuHClP^{tBu}NN^{Bpy} (**5**),[4] RuHClP^{Ph}NN^{Bpy} (**6**),[5] Mn(CO)$_2$BrP^{tBu}NN^{BPy} (**7**),[6] and Mn(CO)$_2$BrP^{tBu}NN$_{tBu}$H (**8**)[7] were prepared according to the previously reported procedures. All catalysts were weighed inside nitrogen filled glove box. Reagent grade amines, alcohols and hexyl hexanoate were purchased from commercial sources and used without further purification. All solvents were purified according to standard procedures under an argon atmosphere, sparged with argon, and stored over 4 Å molecular sieves. Liquid amines, alcohols and esters were purged with argon for half an hour prior to their use. 1,3,5-trimethylbenzene (mesitylene) and potassium tert-butoxide were purchased from commercial sources and used as received.

NMR spectra were recorded at room temperature on a Bruker AMX-300 (300 MHz) or AMX-400 (400 MHz) or AMX-500 (500 MHz) spectrometers. Chemical shifts of the NMR spectra are reported relative to residual signals of $CDCl_3$ (^{1}H NMR: δ = 7.26 ppm, ^{13}C NMR: δ = 77.16 ppm), benzene-D_6 (^{1}H NMR: δ = 7.16 ppm, ^{13}C NMR: δ = 128.06 ppm), CD_2Cl_2 (^{1}H NMR: δ = 5.32 ppm, ^{13}C NMR: δ = 54.00 ppm) or the internal standard mesitylene. ^{31}P{^{1}H} NMR chemical shifts are reported in ppm downfield from H_3PO_4 and referenced to an external 85% solution of phosphoric acid in D_2O. GC–MS was carried out on HP 6890 / 5973 (MS detector) instruments equipped with a 30 m column (Restek 5MS, 0.32 mm internal diameter) with a 5% phenylmethylsilicone coating (0.25 mm) and helium as carrier gas. GC was carried out on HP 6890 or Agilent 7890B Series GC System with N_2 or Helium as carrier gas. IR spectra were recorded on a Nicolet FTIR spectrophotometer (KBr, thin Film). Optical rotations were measured in a PerkinElmar 341 series polarimeter with sodium lamp.

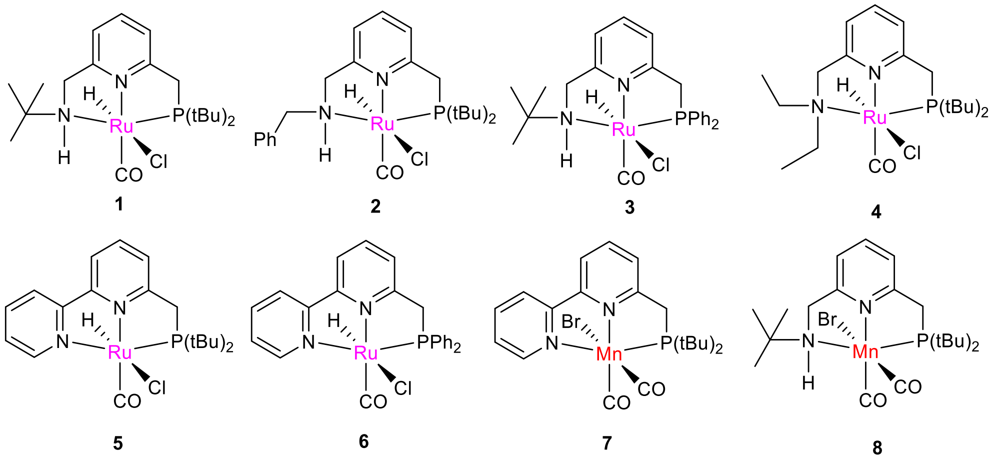

Figure S1. Complexes screened for near-ambient temperature dehydrogenative amide synthesis in this study.

2. ^{1}H and ^{31}P{^{1}H} NMR spectra of selected complexes

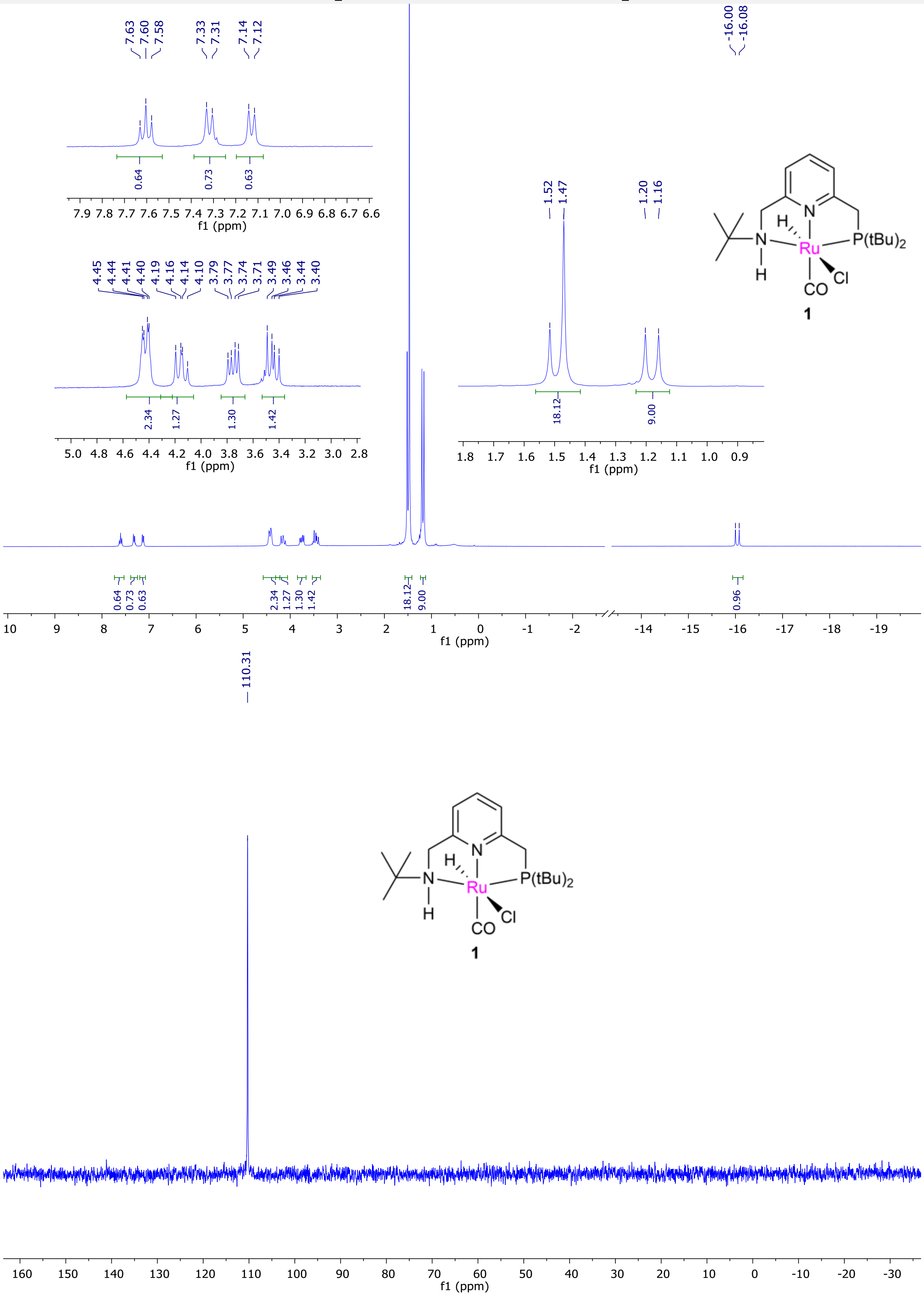

Figure S2. ^{1}H (top, 300 MHz) and ^{31}P{^{1}H} (bottom, 121 MHz) NMR spectra of complex **1** in C_6D_6.

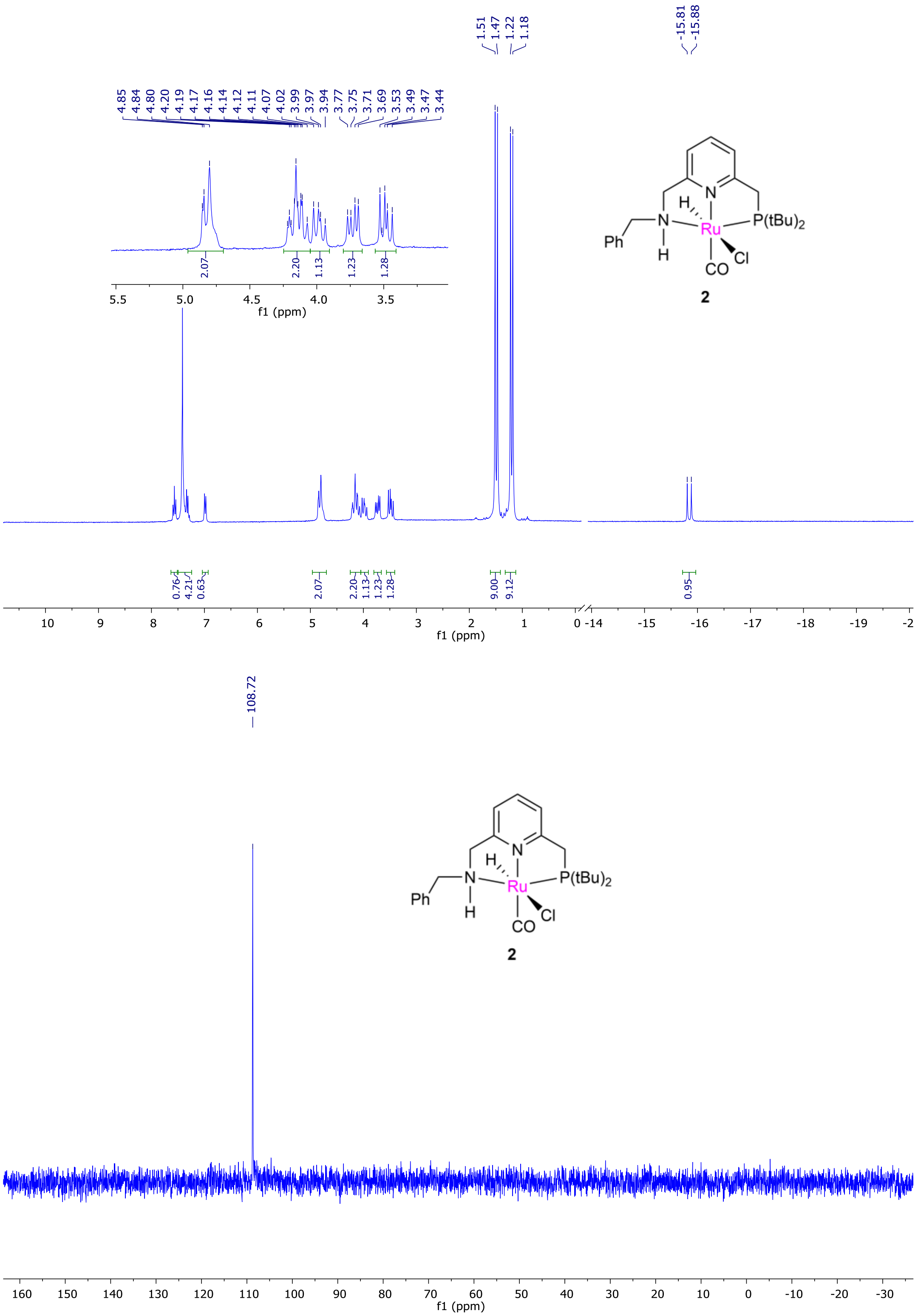

Figure S3. ^{1}H (top, 300 MHz) and $^{31}P\{^{1}H\}$ (bottom, 121 MHz) NMR spectra of complex **2** in C_6D_6.

3. Standard procedure for the dehydrogenative coupling

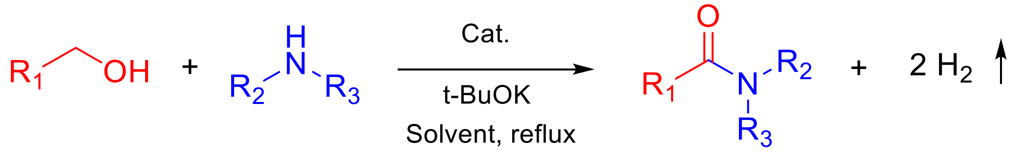

In a N_2 glove box, 5 µmol of ruthenium catalyst and 10 µmol of *t*-BuOK were dissolved in 1 mL of dry solvent (diethyl ether or methyl *tert*-butyl ether (MTBE) or toluene) in a 5 mL vial. The solution was stirred for 5 mins after which a solution of pre-dissolved alcohol (0.5 mmol) and amine (0.55 mmol) in 1 mL dry solvent was added to the initial solution. The resulting solution was transferred to a Schlenk tube. The tube was sealed, taken out of the box, and was connected to a condenser (ethylene glycol/ water: T = 1 ^{o}C) under argon flow. The Schlenk tube was subsequently dipped into a preheated oil bath (50 ^{o}C for Et_2O; 70 ^{o}C for MTBE; and 130 ^{o}C for toluene as solvent) and the solution was refluxed under argon flow for the given time. Afterwards, the reaction solution was cooled to room temperature. A known amount of mesitylene was added to the solution as an internal standard. For some reactions where amide precipitation from solution was observed, THF was added to produce a homogeneous solution. A sample of the resulting solution was then analyzed by GC-MS and ^{1}H NMR with $CDCl_3$ as the deuterated solvent. NMR yields were calculated from integration ratios between amide characteristics peaks and mesitylene methyl protons peak ($\delta \sim 2.2$ ppm). The amides were isolated as described below.

For Table 2, entries 2, 4, 16

The solvent, mesitylene standard, and any unreacted alcohol and amine were removed in vacuo to afford almost pure amide. The resulting solid was washed with pentane and subsequently recrystallized in cold ethyl acetate / hexane solvent mixture.

For Table 2, entries 1,3,5,7,11,13

For amides which were viscous oils, after removing all the volatile solvent and reactants the remaining oily substance was extracted with hexane. Afterwards the hexane was removed in vacuo to obtain pure amides.

For Table 2, entry18, diamide

White precipitation of the amide was observed during the reaction. The amide was isolated by filtering the solid from the solution after the reaction and then was washed with pentane to afford pure amide.

The chiral amides from Table 2, entries 19-20 were purified via column chromatography (ethyl acetate/ hexane)

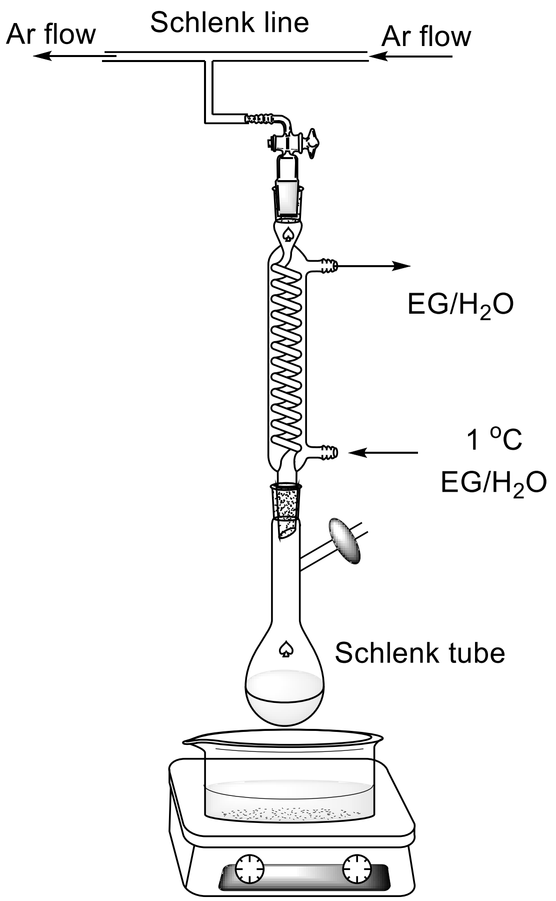

Figure S4. Schematic representation of the reaction setup

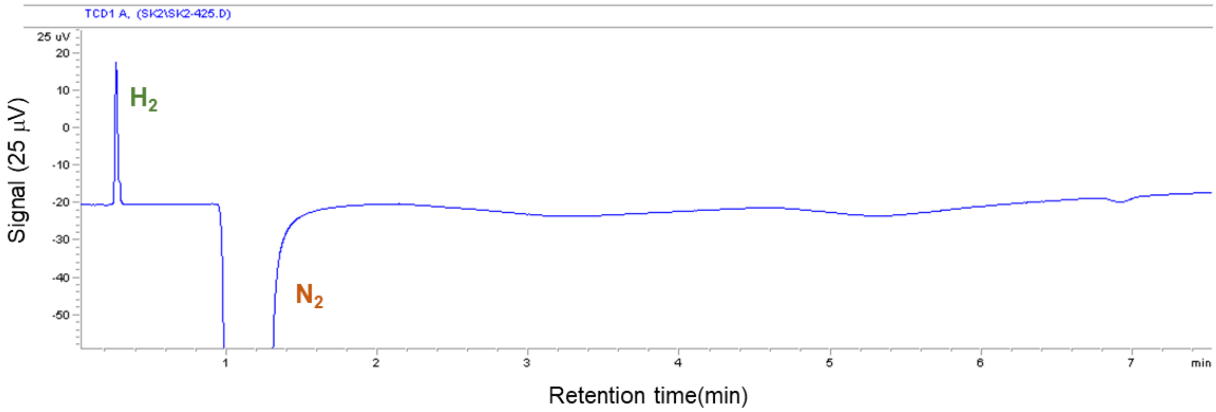

Figure S5. Detection of H_2 gas after the reaction in a closed vessel. Helium was used as the carrier gas.

4. ^{1}H NMR spectra of selected crude reaction mixtures

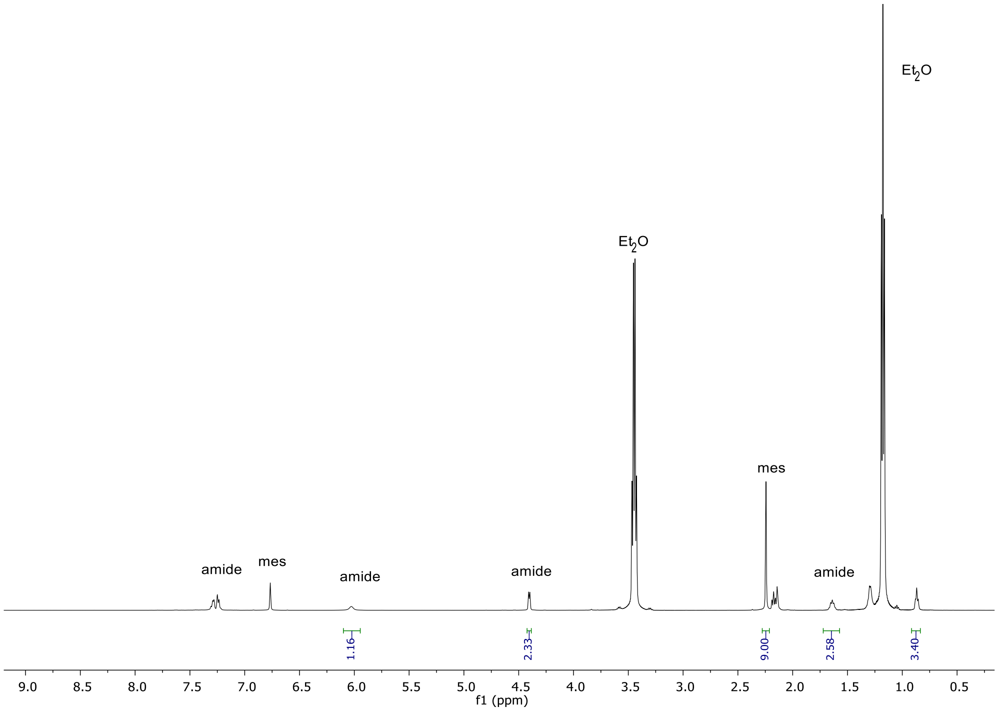

Figure S6. ^{1}H NMR spectra of the crude reaction mixture after dehydrogenative synthesis of N-benzyllhexanamide (Table 2, entry 2) in $CDCl_3$.

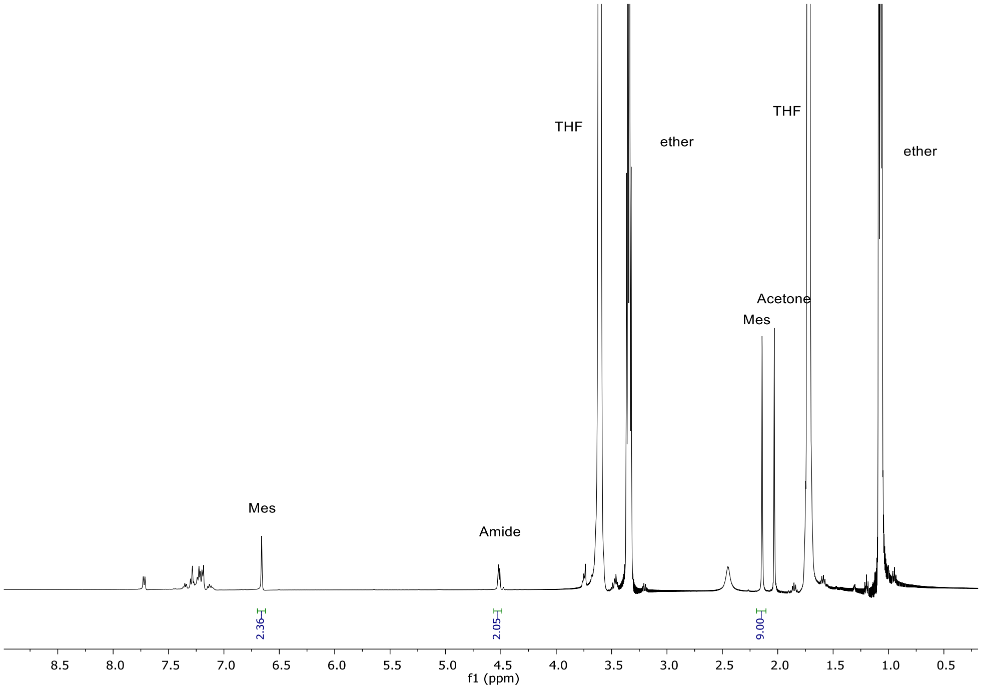

Figure S7. ^{1}H NMR spectra of the crude reaction mixture after dehydrogenative synthesis of N-benzylbenzamide (Table 2, entry 4) in $CDCl_3$. THF was added after the reaction to dissolve precipitating amide. Acetone impurity from NMR tube.

5. Mechanistic reactions

5.1. Binding of different substrates to the dearomatized/ deprotonated PNNH complexes

A scheme summarizing the different binding modes of different substrates to the dearomatized/ deprotonated ruthenium PNNH complexes is shown below, as observed during the course of this study.

Scheme S1

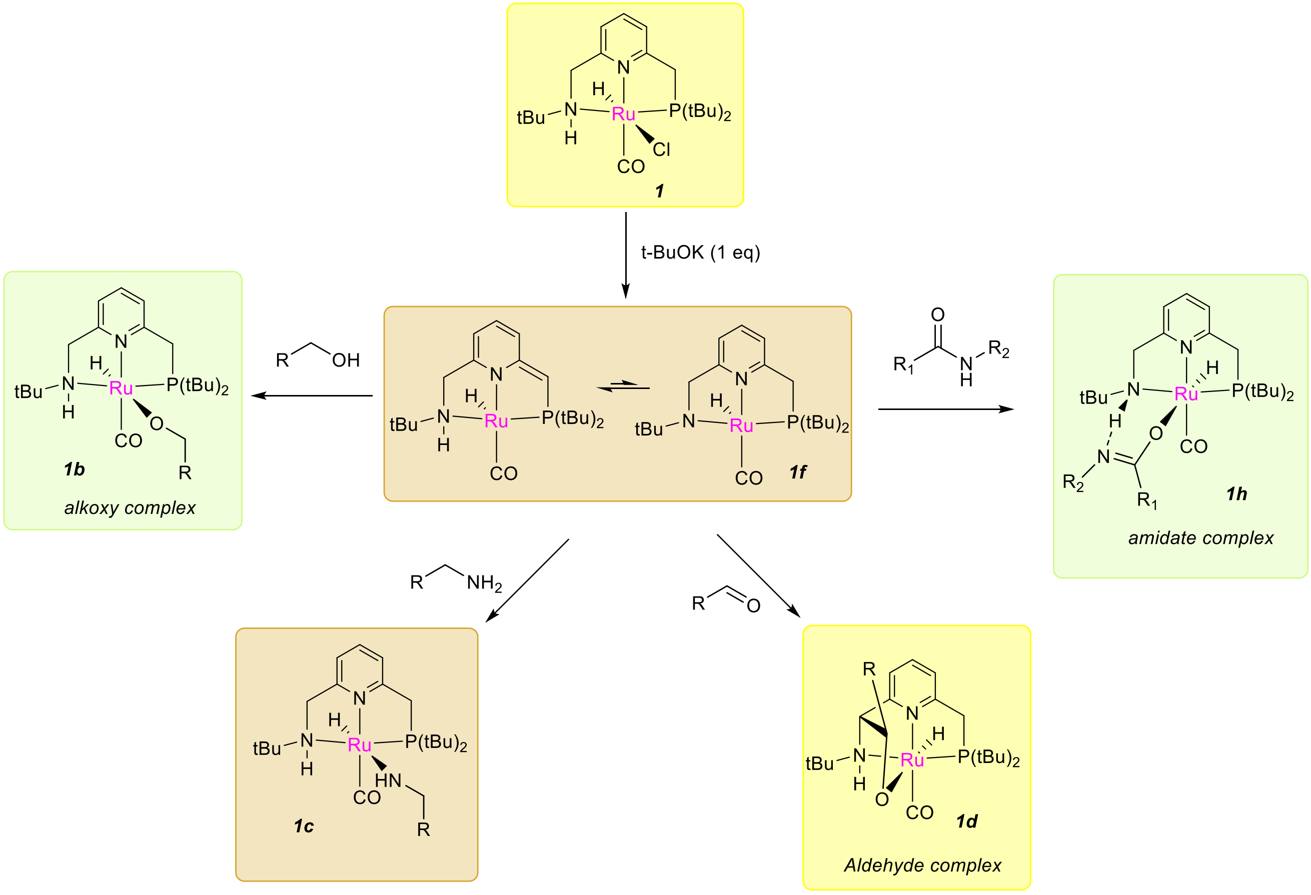

5.2. Synthesis of complex **1d**

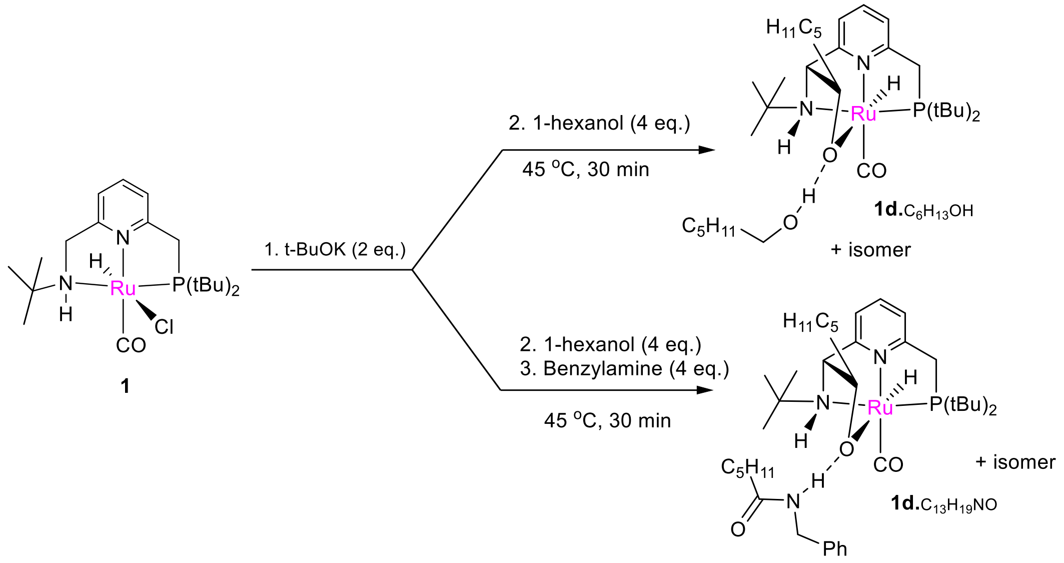

In a 20 mL vial, 30 mg (63 µmol) of complex **1** was dissolved in 1 mL diethylether. 14.1 mg of t-BuOK was then added to the solution upon which the solution color changed to violet from light yellow. The solution was stirred for 5 mins after which 26 mg of 1-hexanol (pre-dissolved in 1 mL Et_2O) was added to the solution. Subsequently, the resulting green colored solution was filtered through a short Celite® pad and transferred to a J. Young NMR tube. The tube was then heated at 45 °C for half an hour and the green solution turned yellow. Afterwards, the tube was taken back inside the box and the solvent and excess hexanol was removed in vacuum to afford complex **1d**.$C_6H_{13}OH$. For **1d**.$C_{13}H_{19}NO$, 4 eq of amine was also added alongside 4 eq of 1-hexanol with rest of the procedure being unchanged. ^1H and ^{31}P NMR spectra suggests that at room temperature, **1d** exist as two isomers in 9:1 ratio. Single crystals of **1d** suitable for XRD analysis were grown by dissolving **1d** in a mixture of THF/pentane followed by slow evaporation of the solvent.

$^{31}P\{^1H\}$ NMR (**1d**.$C_6H_{13}OH$) (97 MHz, Benzene-d_6): δ 119.5 (s) (major, 91%); 120.18 (s) (minor, 9%)
^1H NMR (**1d**.$C_6H_{13}OH$) (400 MHz, Benzene-d_6) (major): δ 6.93 (t, *J* = 8.0 Hz, 1H), 6.57 (dd, *J* = 8.4, 3.0 Hz, 2H), 4.64 (dd, *J* = 8.5, 4.1 Hz, 1H), 4.22 (s, 1H), 4.15 (s, 1H), 3.98 (t, *J* = 6.4 Hz, 2H), 2.97 (d, *J* = 8.6 Hz, 2H), 2.03 – 1.90 (m, 1H), 1.85 (p, *J* = 6.7 Hz, 2H), 1.62 (dddt, *J* = 27.0, 16.1, 7.9, 3.8 Hz, 2H), 1.55 – 1.39 (m, 11H), 1.35 (d, *J* = 12.9 Hz, 9H), 1.30 (d, *J* = 13.0 Hz, 9H), 1.22 – 1.12 (m, 4H), 1.09 (s, 9H), 1.06 – 0.88 (m, 4H), 0.74 – 0.55 (m, 1H), -15.12 (d, *J* = 28.6 Hz, 1H).

^{13}C NMR (**1d**.$C_6H_{13}OH$) (126 MHz, Toluene-d_8) (major): δ 208.18 (d, *J* = 15.6 Hz), 161.90 (d, *J* = 2.7 Hz), 159.98 (d, *J* = 4.3 Hz), 134.88, 118.35, 118.12 (d, *J* = 8.4 Hz), 77.64, 70.66, 61.96, 54.12, 38.16, 37.83 (d, *J* = 11.0 Hz), 36.98 (d, *J* = 19.6 Hz), 33.83, 32.58, 32.21, 30.05 (d, *J* = 3.2 Hz), 29.14 (d, *J* = 4.6 Hz), 28.40, 26.38, 26.20, 22.96, 14.14, 14.06.

IR (thin film, KBr) = 1900 cm^{-1} (νCO).

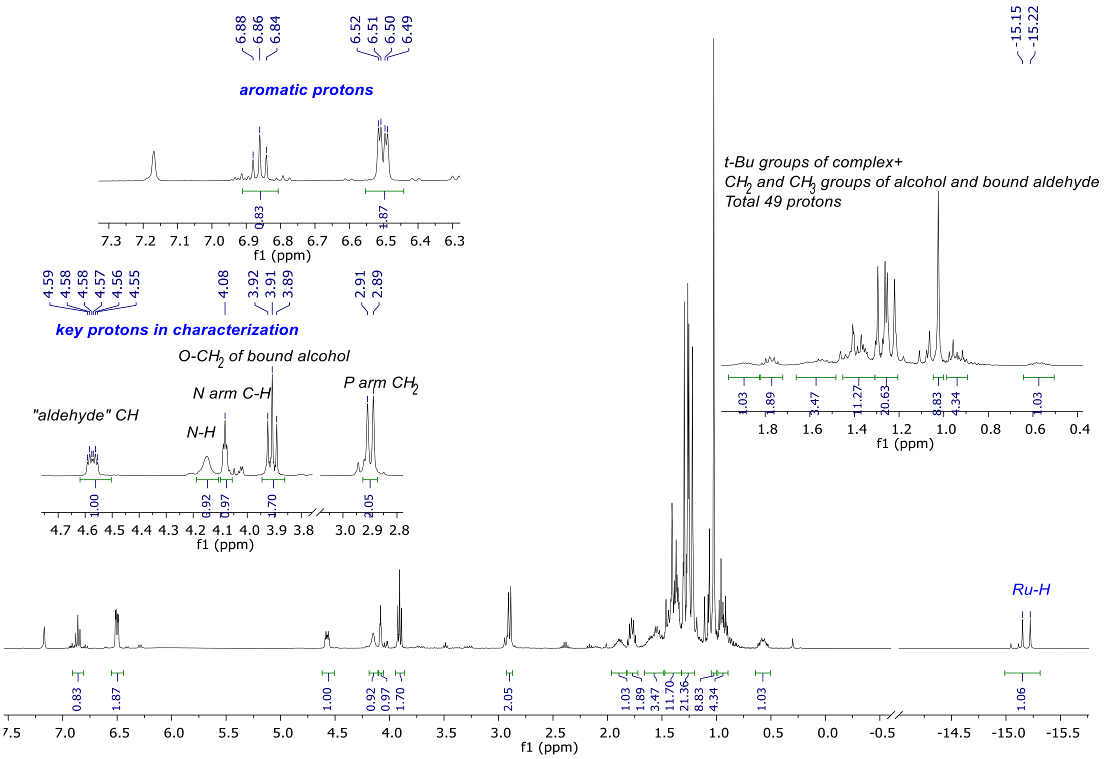

Figure S8. ^{1}H NMR spectrum of complex **1d**.$C_6H_{13}OH$ in C_6D_6.

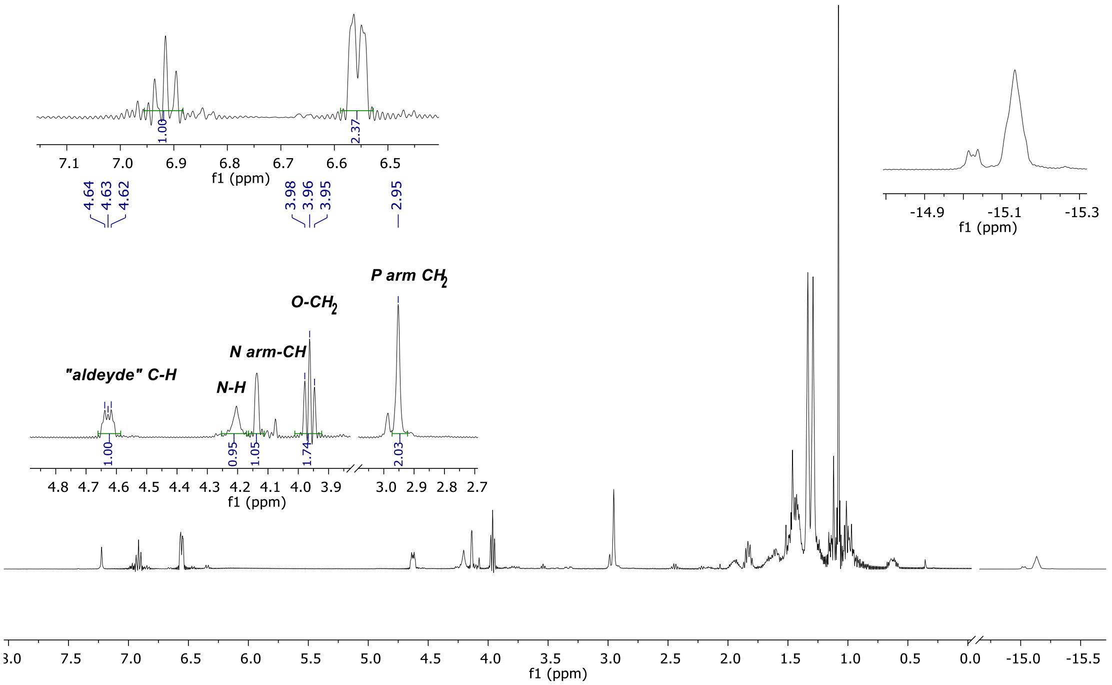

Figure S9. $^{1}H\{^{31}P\}$ NMR spectra of complex **1d**.$C_6H_{13}OH$ in C_6D_6.

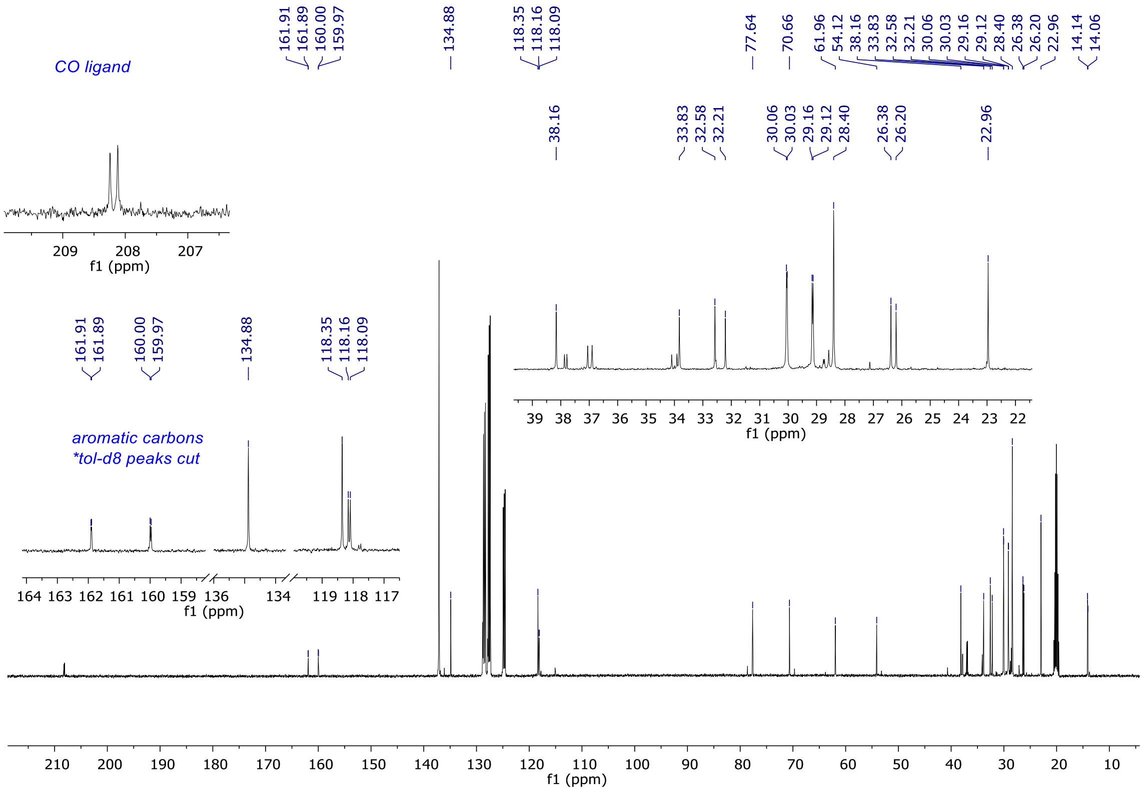

Figure S10. $^{13}C\{^{1}H\}$ NMR spectrum of complex **1d**.$C_6H_{13}OH$ in Tol-d_8.

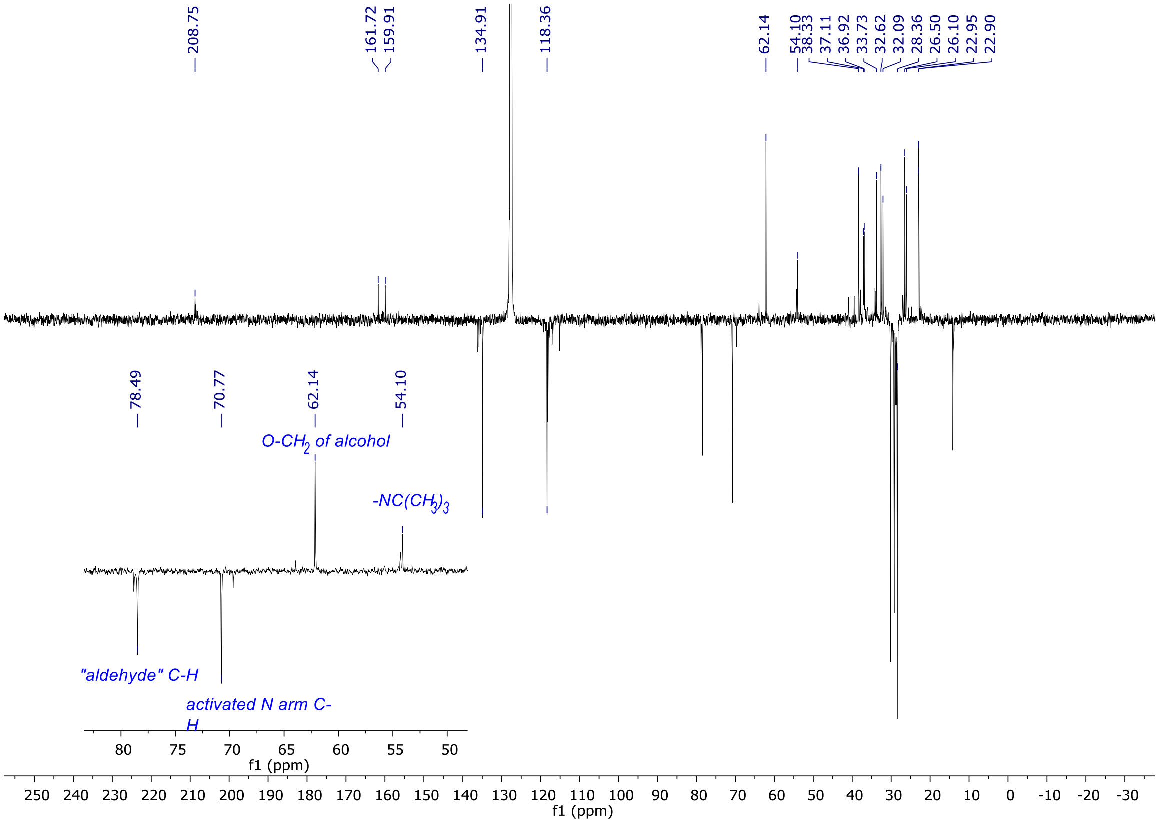

Figure S11. DEPTQ 135 NMR spectrum of complex **1d**.$C_6H_{13}OH$ in C_6D_6.

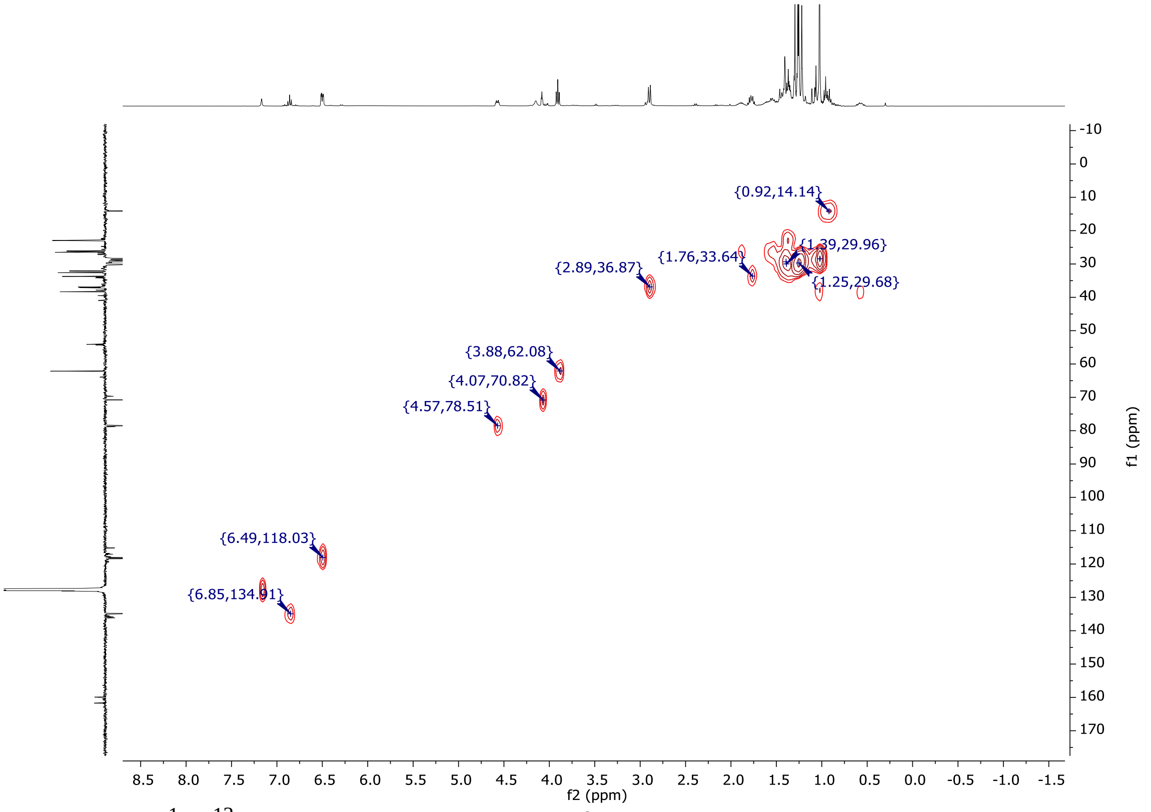

Figure S12. ^{1}H-^{13}C HSQC NMR spectrum of complex **1d**.$C_6H_{13}OH$ in C_6D_6.

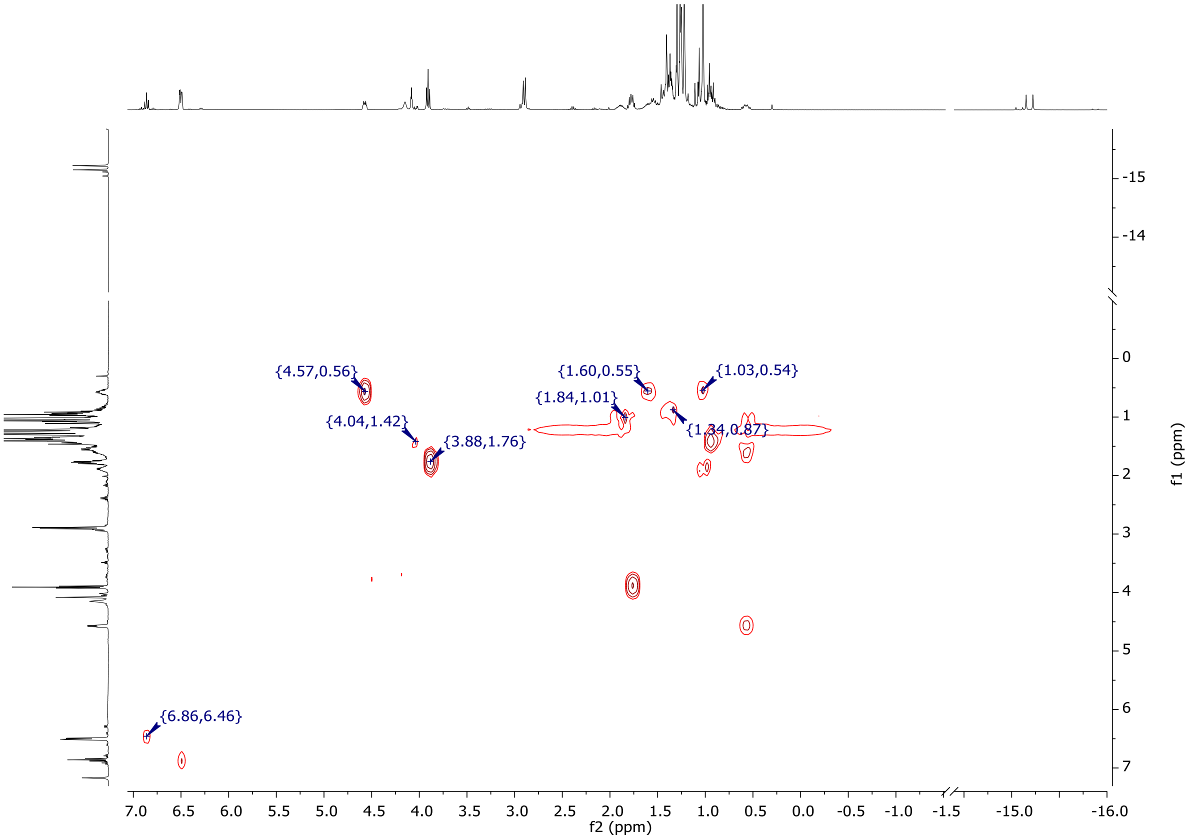

Figure S13. ^{1}H-^{1}H COSY NMR spectrum of complex **1d**.$C_6H_{13}OH$ in C_6D_6.

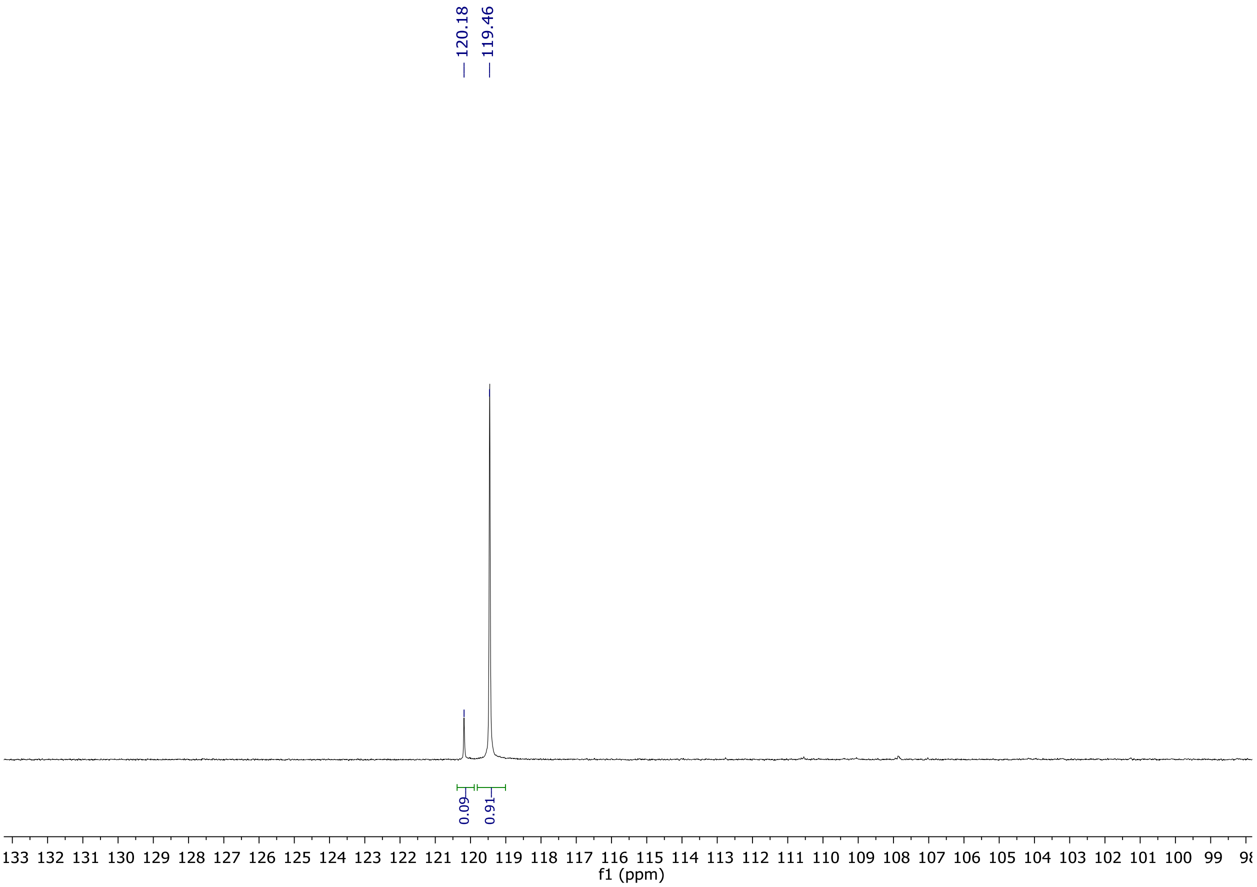

Figure S14. $^{31}P\{^{1}H\}$ NMR spectrum of complex **1d**.$C_6H_{13}OH$ in C_6D_6.

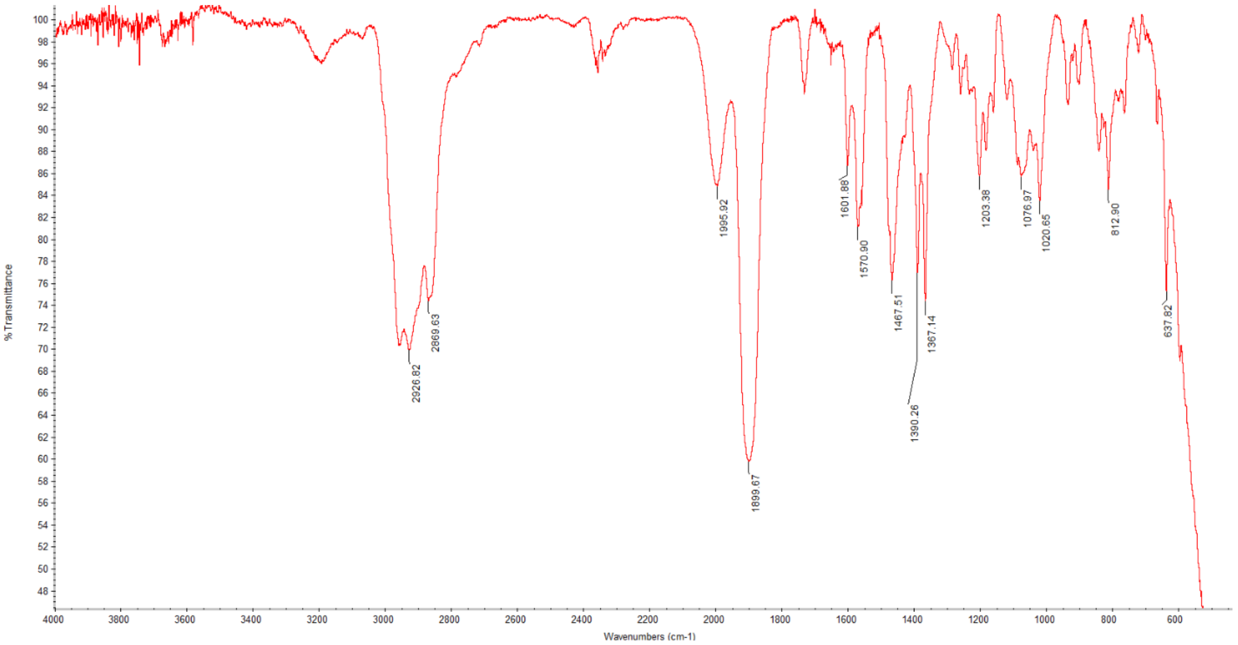

Figure S15. IR spectrum of complex **1d**.$C_6H_{13}OH$ (thin film, KBr).

5.3. Synthesis of complex **9**

5.3.1. Synthesis of the ligand

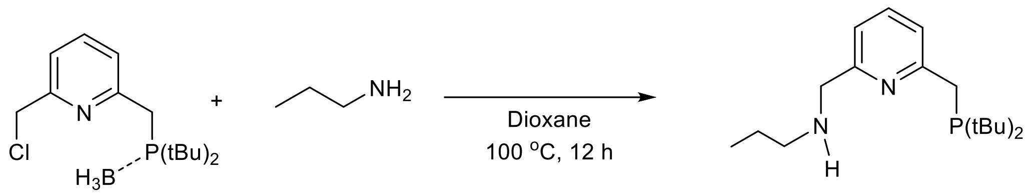

In a 20 mL vial, 100 mg of the precursor was dissolved in 1 mL propylamine. 4 mL dioxane was added to the solution and the resulting mixture was heating at 100 °C in an oil bath with stirring for 16 h inside a closed Schlenk flask. Afterwards, the flask was cooled to room temperature followed by the removal of flask atmosphere through vacuum. The flask was then filled with argon and was heated at 100 °C for an additional 30 min. Afterwards, the flask was cooled down to room temperature and the dioxane and excess amine were removed under vacuum. 10 mL pentane was added to the remaining sticky white solid, stirred and the resulting solution was filtered through a short Celite® pad. The remaining solid was washed again with 10 mL pentane, which was also filtered. Both pentane solutions were combined from which the removal of pentane afforded the desired ligand as a colorless oily liquid with 78% yield.

^{31}P NMR (202 MHz, Toluene-d$_8$) δ 35.77 (s).

^{1}H NMR (500 MHz, Toluene-d$_8$) δ 7.24 (t, *J* = 7.7Hz, 1H), 7.20 (d, *J* = 7.3 Hz, 1H), 6.92 (d, *J* = 7.4 Hz, 1H), 3.85 (s, 2H, NHCH_2Py), 3.05 (bs, 2H, PCH_2Py), 2.51 (t, *J* = 6.8 Hz, 2H, NHCH_2CH$_2$), 2.13 (s, 1H, N*H*), 1.48 (sextet, J = 7.0 Hz, 2H, NHCH$_2$CH_2CH$_3$), 1.13 (bd, J = 10.5 Hz, 18H, PC(CH_3)$_3$), 0.93 (t, J = 7.6 Hz, 3H, NHCH$_2$CH$_2$CH_3).

^{13}C NMR (126 MHz, Toluene-d$_8$) δ 161.53 (d, J = 15.0 Hz, Py-C2), 159.84 (s, Py-C6), 135.63 (s, Py-C4), 121.59 (d, *J*C,P = 9.4 Hz, Py-C3), 118.35 (d, J = 1.8 Hz, Py-C5), 55.45 (s, NH*C*H$_2$Py), 51.38 (s, N*C*H$_2$CH2CH$_3$), 31.95 (d, *J*C,P = 26.0 Hz, P*C*(CH3)3), 31.51 (d, *J*C,P = 24.3 Hz, P*C*H$_2$Py), 29.45 (d, *J*C,P = 13.8 Hz, PC(*C*H3)3), 23.51 (s, NHCH$_2$*C*H$_2$CH3), 11.69 (s, NHCH$_2$CH$_2$*C*H$_3$).

5.3.2. Synthesis of the complex

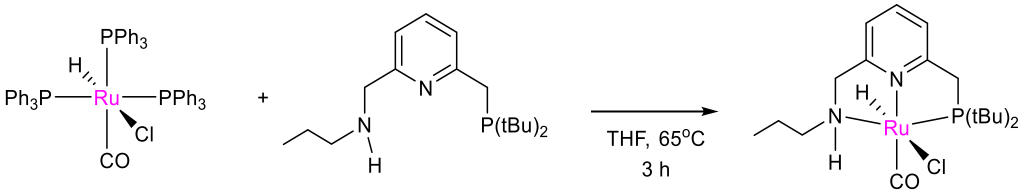

9

In a 20 mL vial, 120 mg (0.126 mmol) of $RuHCl(CO)(PPh_3)_3$ was suspended in 3 mL THF. A solution of 55 mg PNNH(*n*-Pr) ligand (0.178 mmol) in 5 mL THF was added dropwise to the stirring suspension. The resulting solution was transferred to a pressure tube and heated at 66 ^{o}C for 3` hours. THF was then removed from the yellow solution under vacuum till the solution volume became 2 mL. Afterwards, 10 mL of pentane was slowly added to the solution while stirring resulting in the formation of pale yellow precipitates. The supernatant was decanted, and the pale yellow solid was washed with pentane (2x10 mL) and ether (2x5 mL) to afford complex **9** in 72% (43 mg) yield.

^{31}P NMR (202 MHz, Methylene Chloride-d_2) δ 108.74 (s).

^{1}H NMR (500 MHz, Methylene Chloride-d_2) δ 7.68 (t, J = 7.8 Hz, 1H), 7.39 (d, J = 7.9 Hz, 1H), 7.19 (d, J = 7.8 Hz, 1H), 4.58 (dt, J = 15.2, 4.0 Hz, 1H), 4.31 (bs, 1H), 4.11 (dd, J = 15.2, 10.5 Hz, 1H), 3.68 (dd, J = 16.7, 7.7 Hz, 1H), 3.59 – 3.42 (m, 1H), 3.27 – 3.08 (m, 1H), 3.06 – 2.93 (m, 1H), 2.03 – 1.84 (m, 1H), 1.45 (d, J = 13.4 Hz, 9H), 1.19 (d, J = 13.0 Hz, 9H), 1.05 (t, J = 7.5 Hz, 3H), -15.77 (d, J = 23.4 Hz, 1H)

^{13}C NMR (126 MHz, Methylene Chloride-d_2) δ 208.17 (dd, J = 15.5, 3.0 Hz), 160.83 (d, J = 4.2 Hz), 158.64 (d, J = 2.3 Hz), 137.14, 120.99 (d, J = 9.1 Hz), 118.06, 60.95, 59.41, 36.65 (d, J = 16.3 Hz), 29.92 (d, J = 4.1 Hz), 28.26, 22.73, 11.19.

IR (thin film, KBr) = 1898 cm^{-1} (ν_{CO})

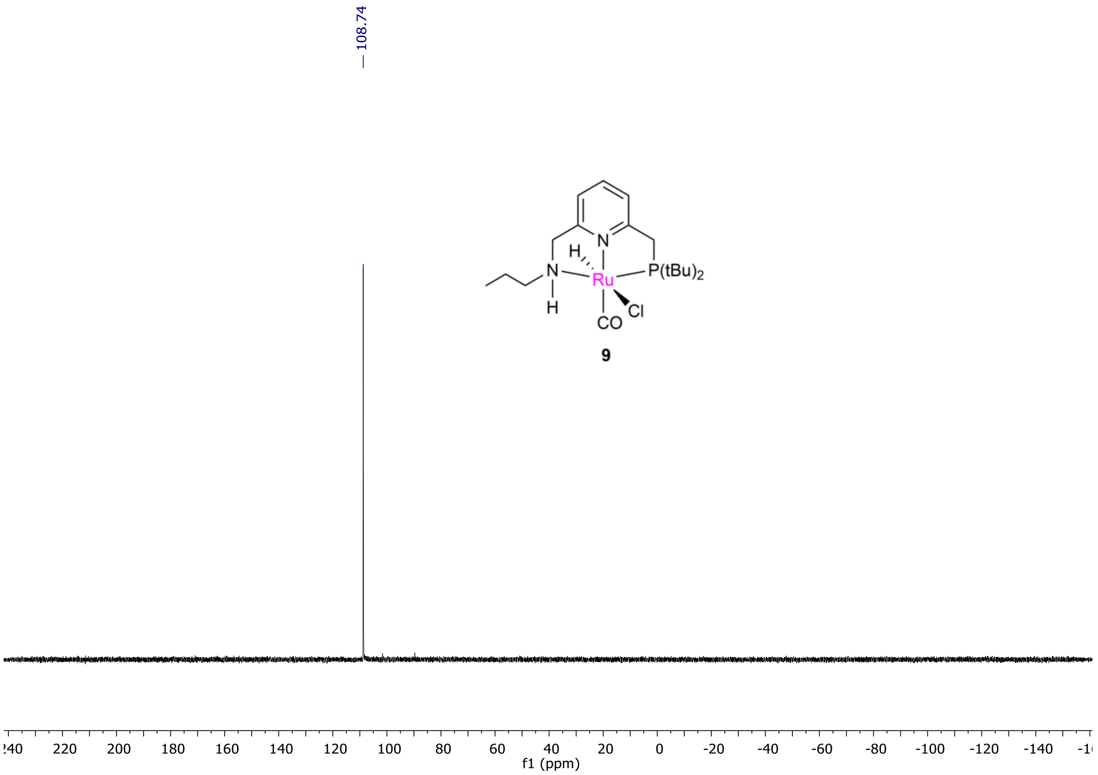

Figure S16. $^{31}P\{^1H\}$ NMR spectrum of complex **9** in CD_2Cl_2.

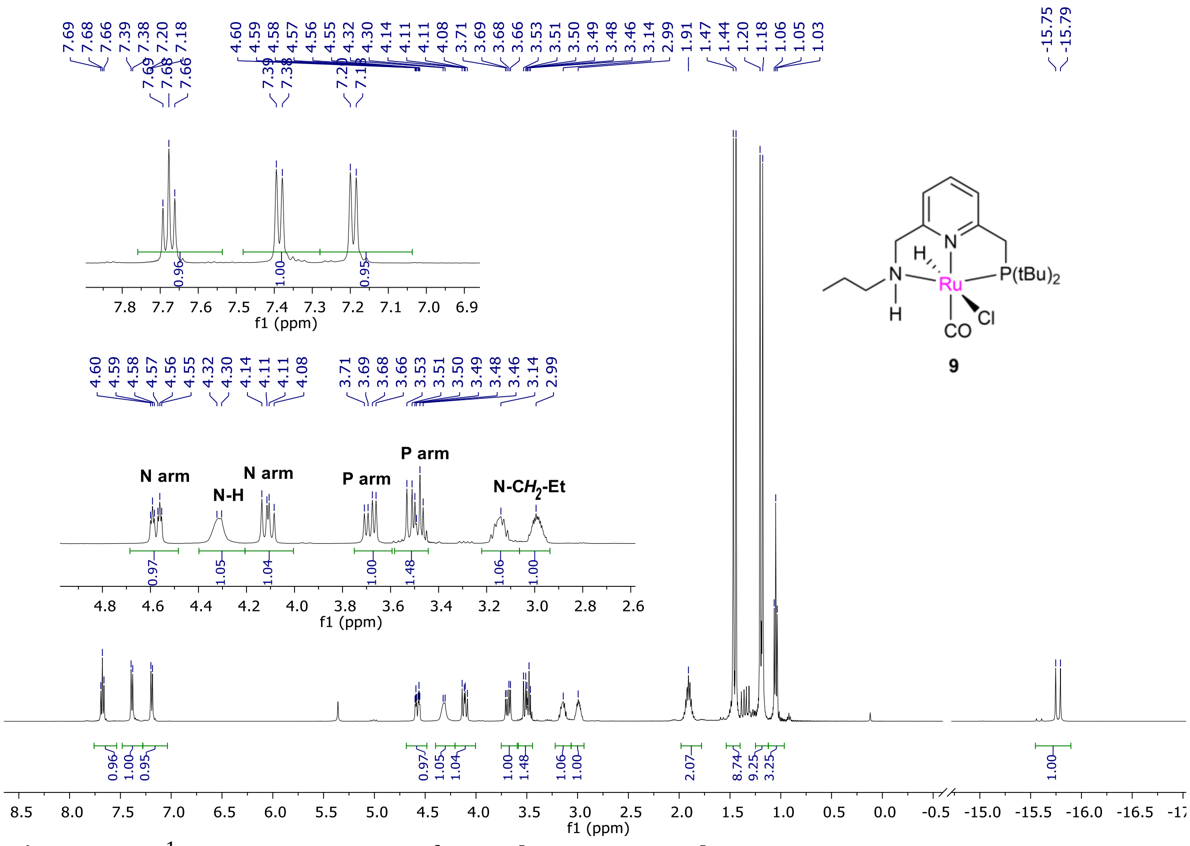

Figure S17. ^1H NMR spectrum of complex **9** in CD_2Cl_2.

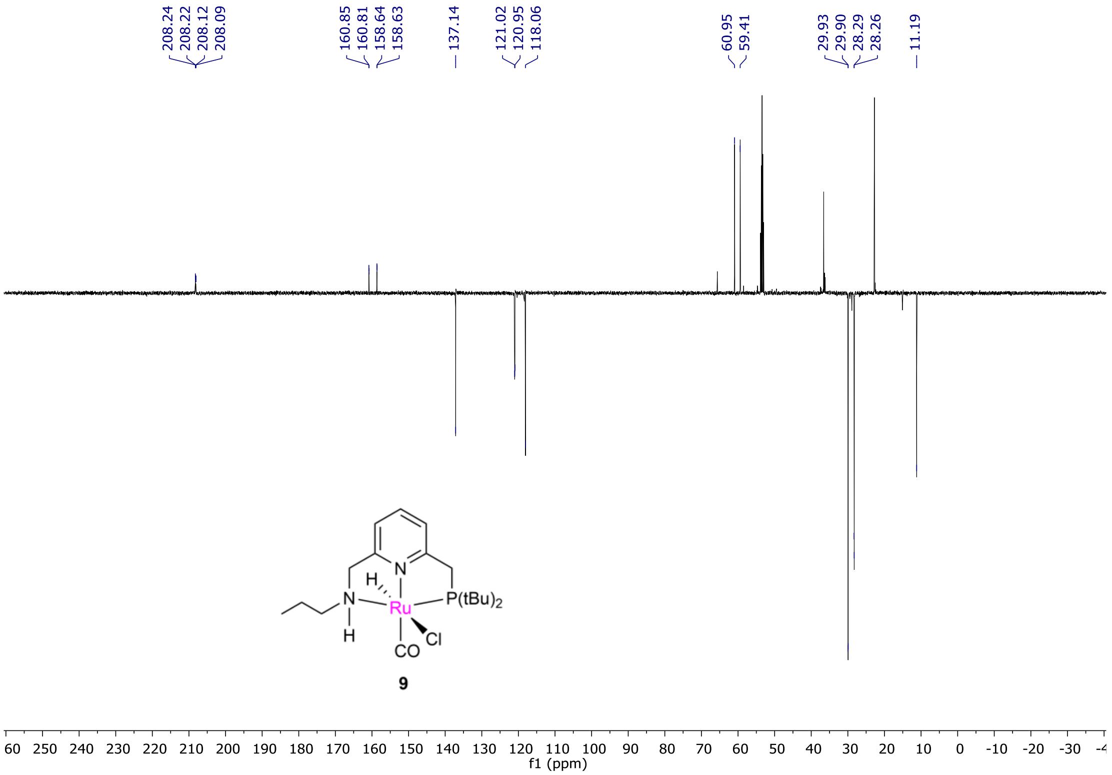

Figure S18. DEPTQ 135 NMR spectrum of complex **9** in CD_2Cl_2.

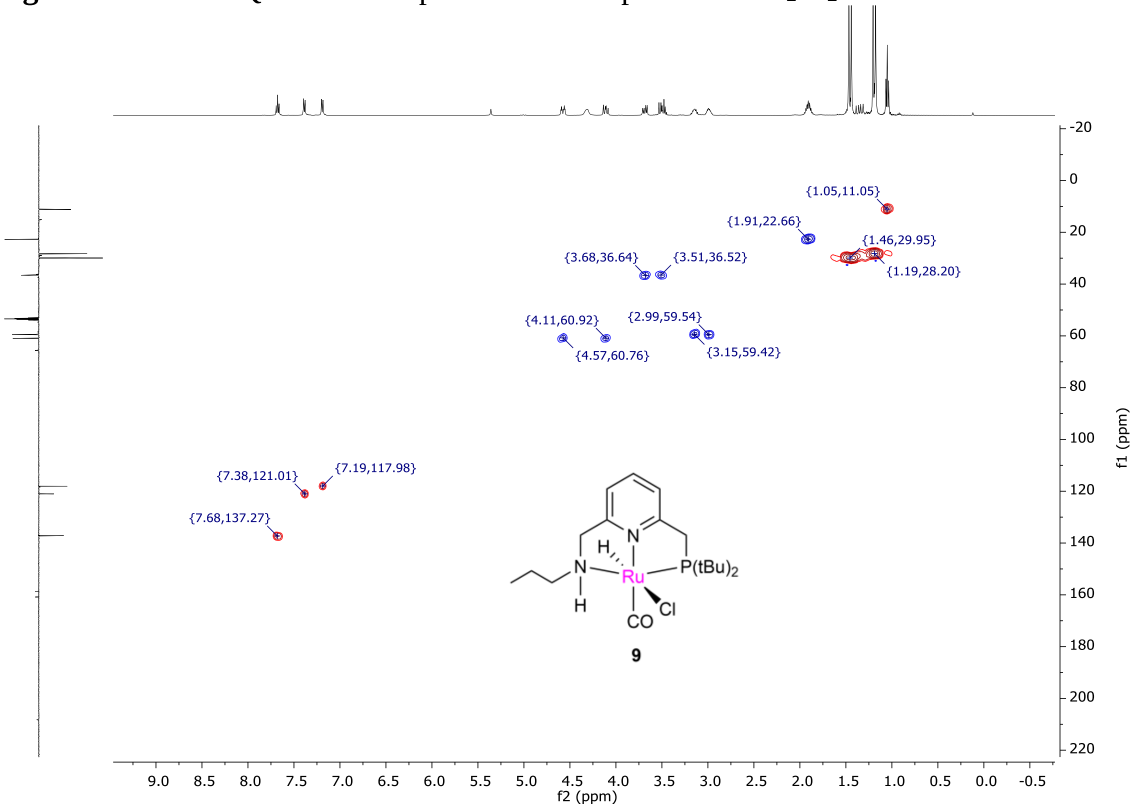

Figure S19. ^{1}H-^{13}C HSQC NMR spectrum of complex **9** in CD_2Cl_2.

5.4. NMR observations

*a) Reaction of complex **1** with t-BuOK, and hexanol*

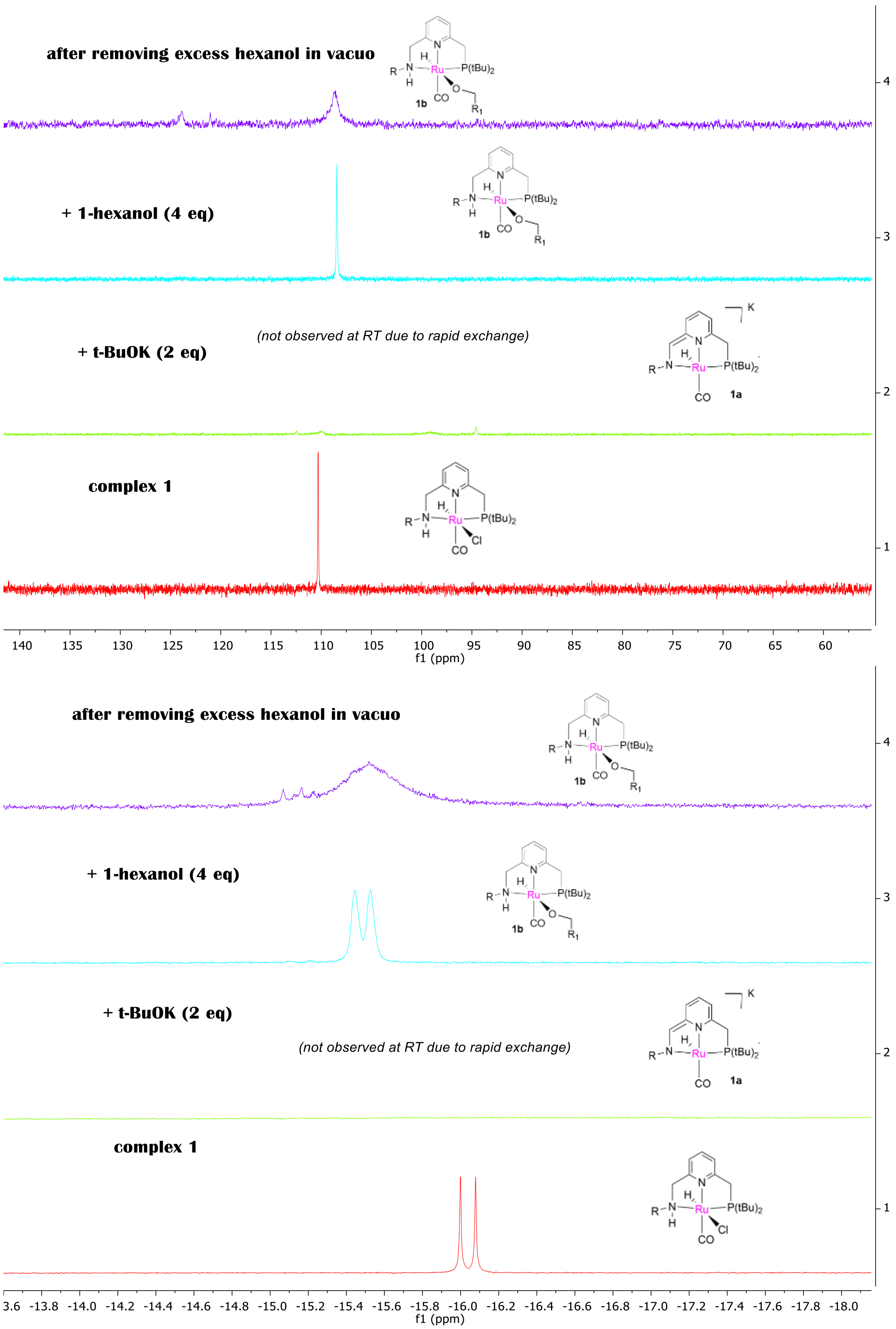

Figure S20. Reaction of complex **1** with t-BuOK, and hexanol in Et_2O as observed in $^{31}P\{^1H\}$(top) and ^1H (bottom, hydride region) NMR. [R = t-Bu; $R_1 = C_5H_{11}$]

*b) Reaction of complex **1** with t-BuOK, benzylamine and hexanol*

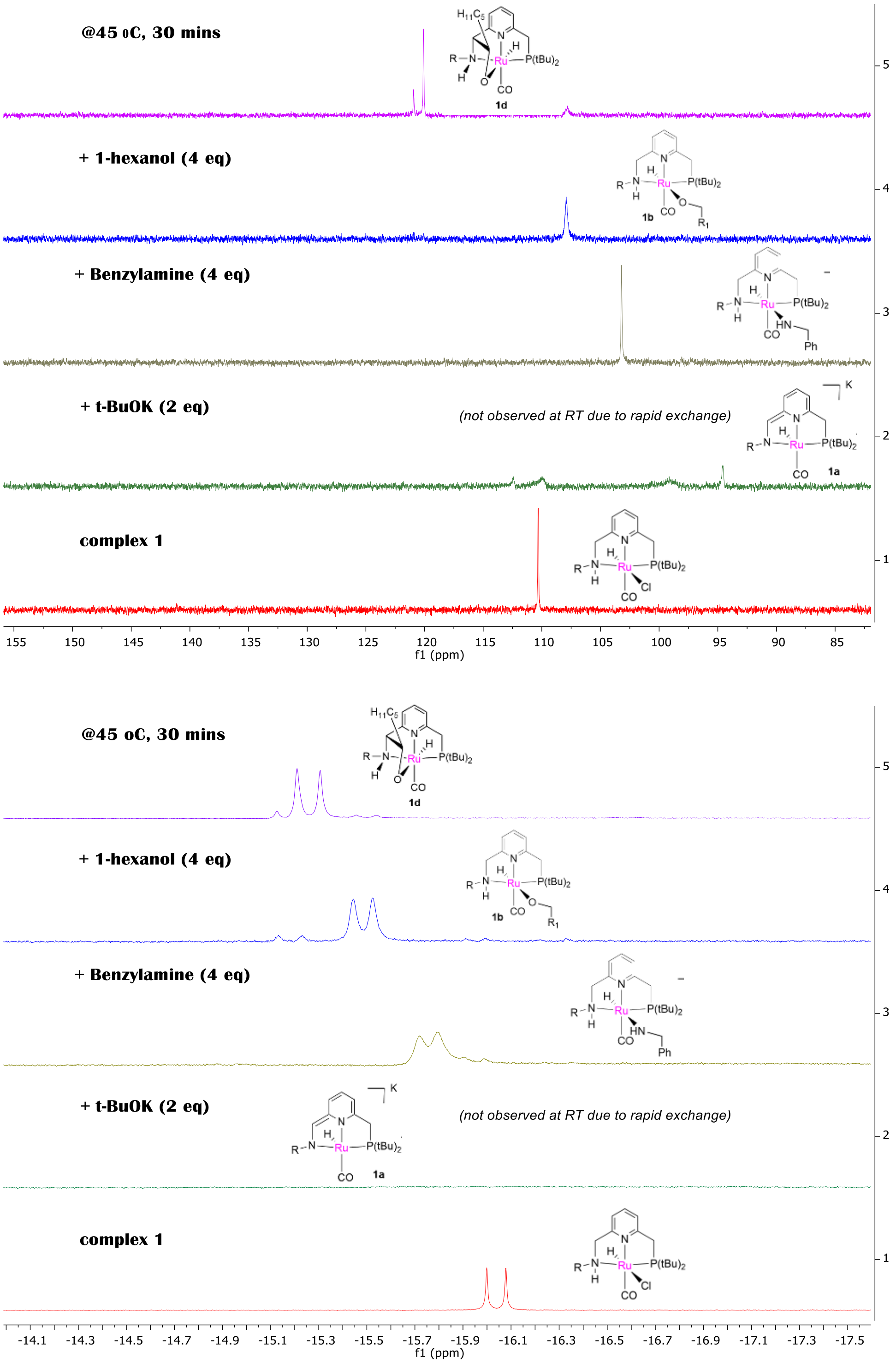

Figure S21. Reaction of complex **1** with t-BuOK, benzylamine and hexanol in Et_2O as observed in $^{31}P\{^{1}H\}$ and ^{1}H (hydride region) NMR. [R = t-Bu]

*c) Reaction of complex **4** with t-BuOK, benzylamine and hexanol*

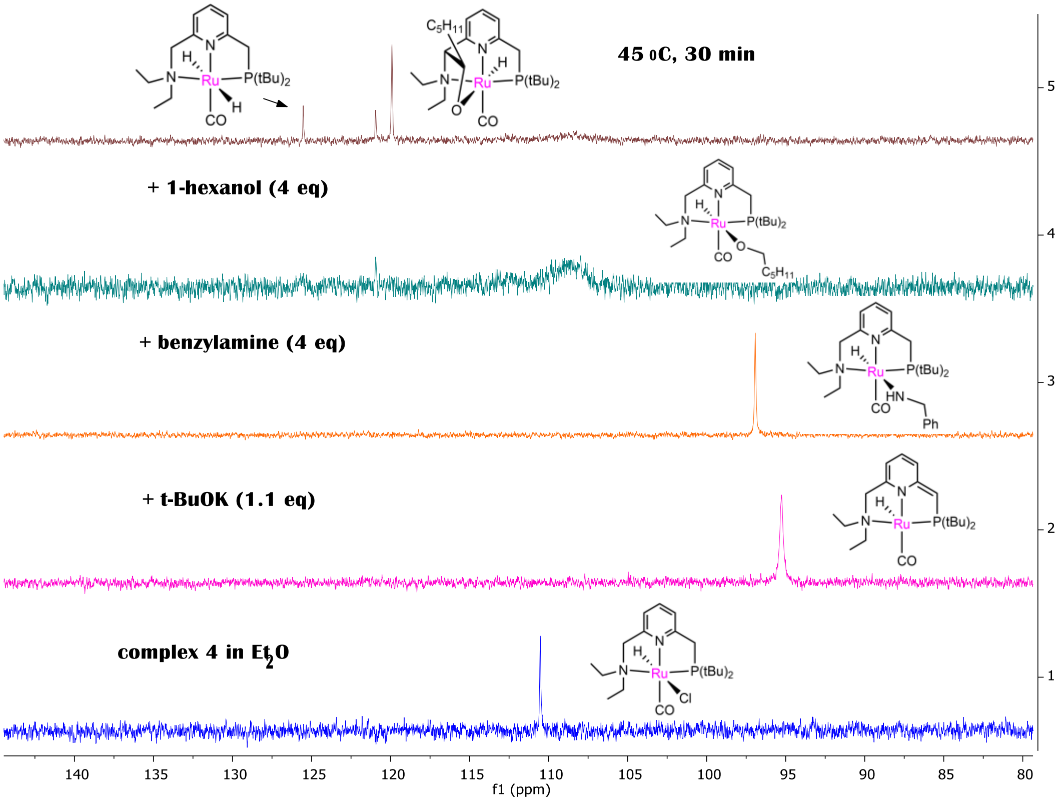

Figure S22. Reaction of complex **4** with t-BuOK, benzylamine and hexanol in Et_2O as observed in $^{31}P\{^1H\}$ NMR.

d) aldehyde exchange experiments

Exchange of bound hexanal with benzaldehyde

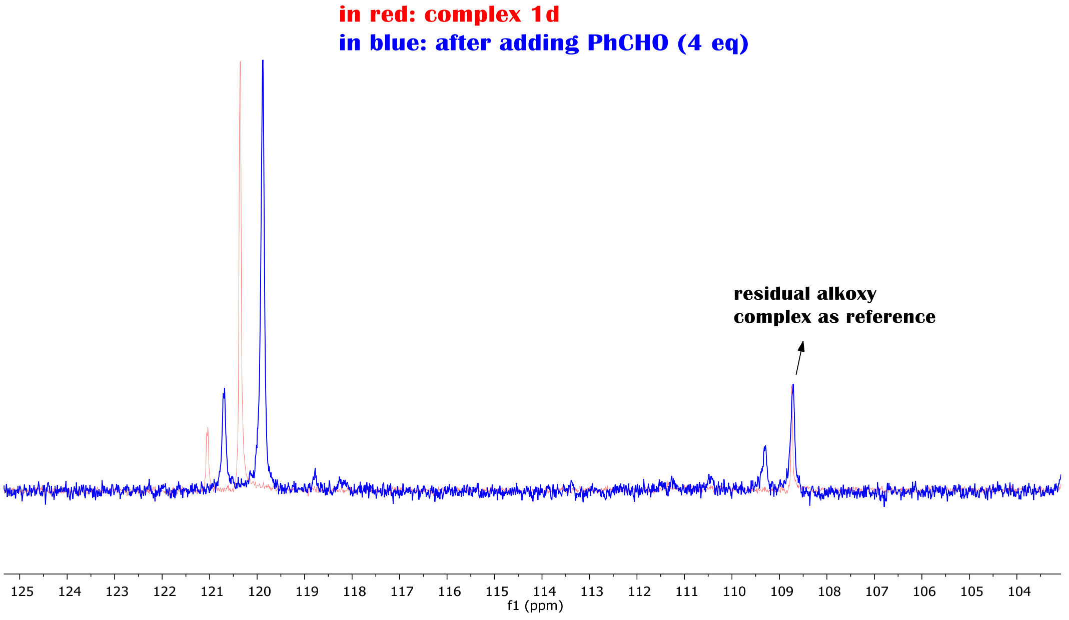

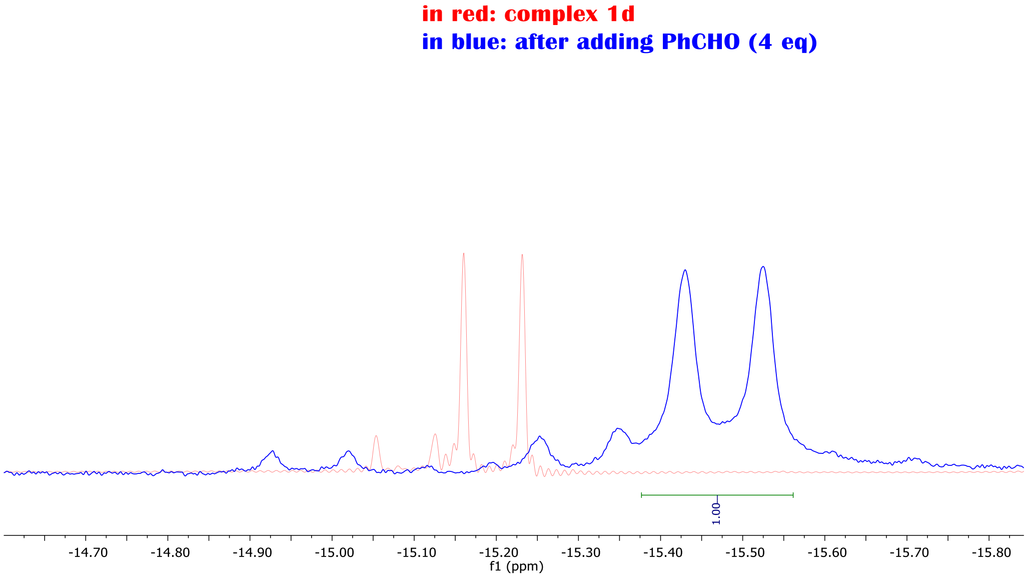

Figure S23. Exchange of the bound aldehyde of **1d** with free benzaldehyde in solution as observed in ^{1}H and $^{31}P\{^{1}H\}$ NMR.

Exchange of bound benzaldehyde with hexanal

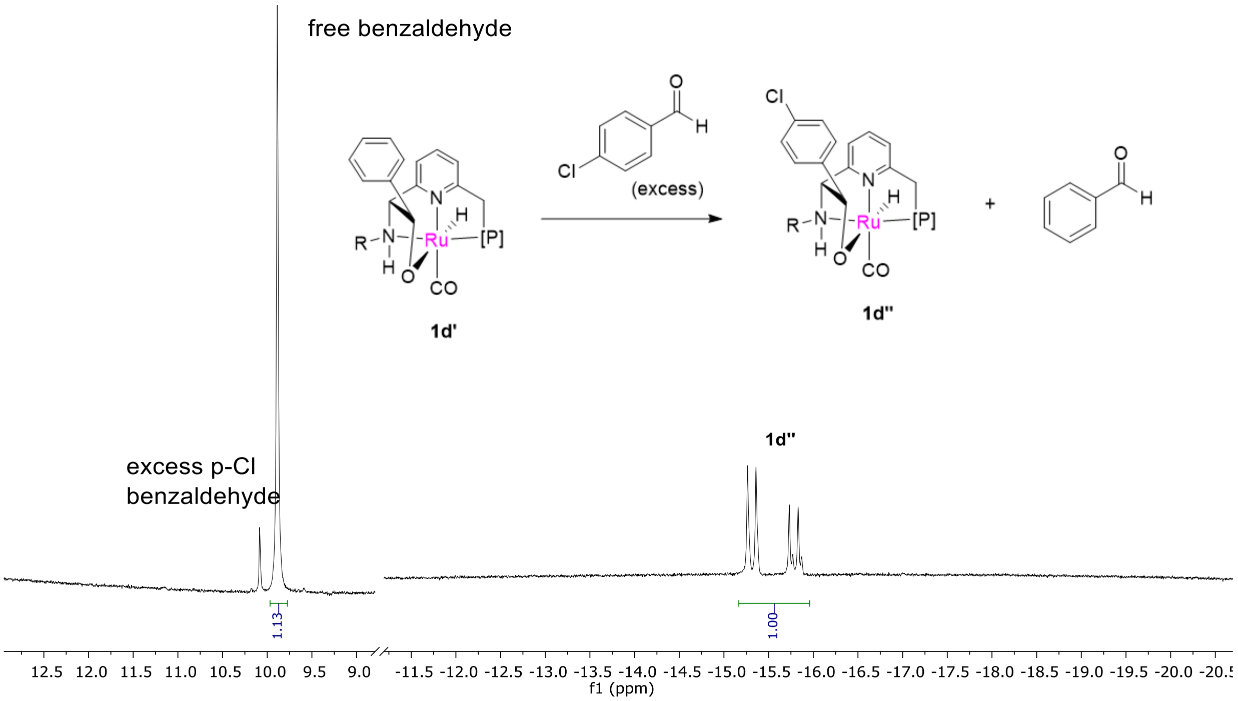

Figure S24. Exchange of bound benzaldehyde with *p*-Cl benzaldehyde. The complex **1d'** was synthesized by heating a diethyl ether solution the alkoxy complex at 45 ^{o}C for 1 hour. The free benzaldehyde is observed in the solution after adding excess *p*-Cl benzaldehyde. ^{1}H spectrum is shown only for the hydride and aldehyde region for clarity.

Exchange of bound benzaldehyde with p-Cl benzaldehyde

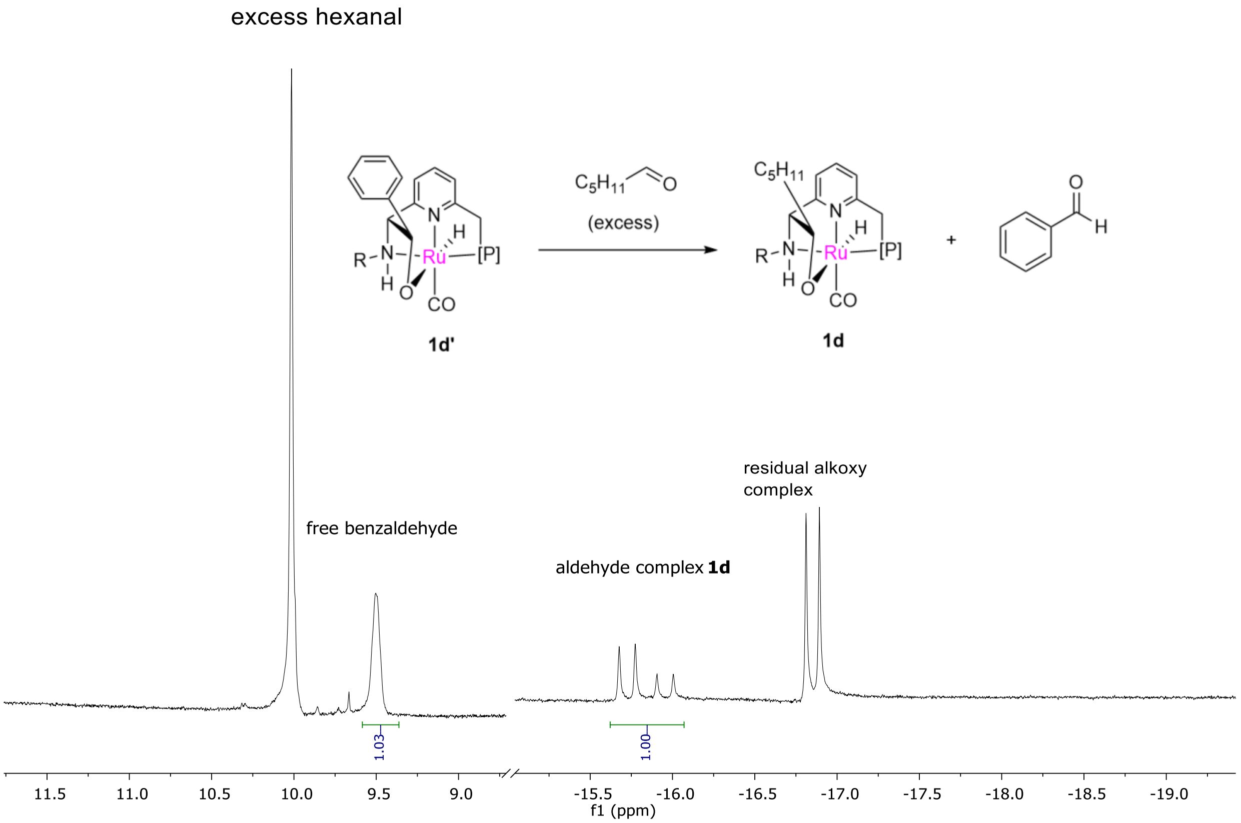

Figure S25. Exchange of bound benzaldehyde with hexanal. The complex **1d'** was synthesized by heating a diethyl ether solution the alkoxy complex at 45 °C for 15 min, which only resulted in partial conversion (rest of the alkoxy complex is seen in the spectrum). The free benzaldehyde is observed in the solution after adding excess hexanal. ^{1}H spectrum is shown only for the hydride and aldehyde region for clarity.

e) *Formation of the amidate complex from* ***1d*** *after addition of amine and regeneration of the aldehyde bound complex upon addition of alcohol*

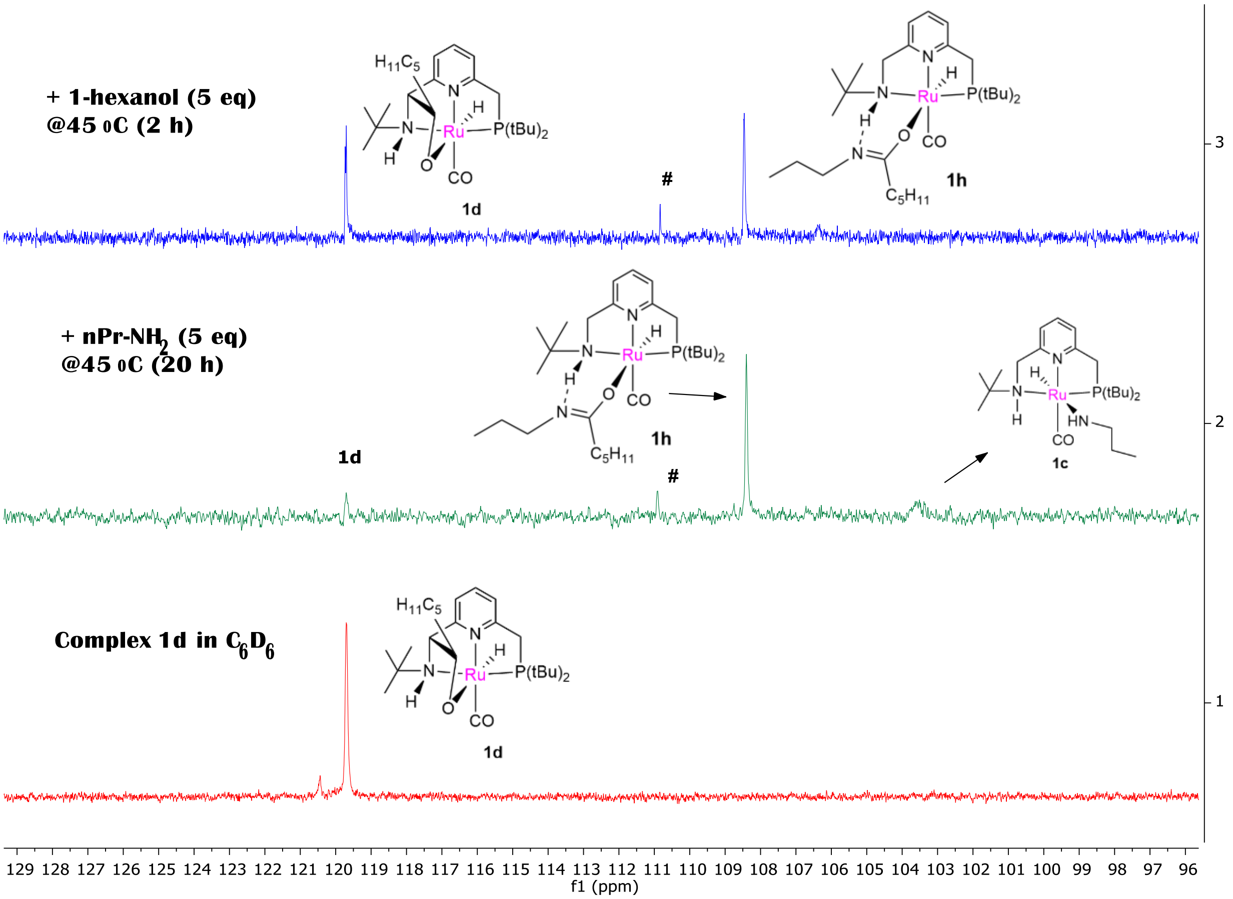

Figure S26. Formation of the amidate (and amido in minor amounts) complex from the aldehyde complex upon addition of amine and mild heating (panel 2), and the regeneration of **1d** upon addition of alcohol and mild heating (panel 3), as observed from $^{31}P\{^{1}H\}$ NMR. #unidentified species.

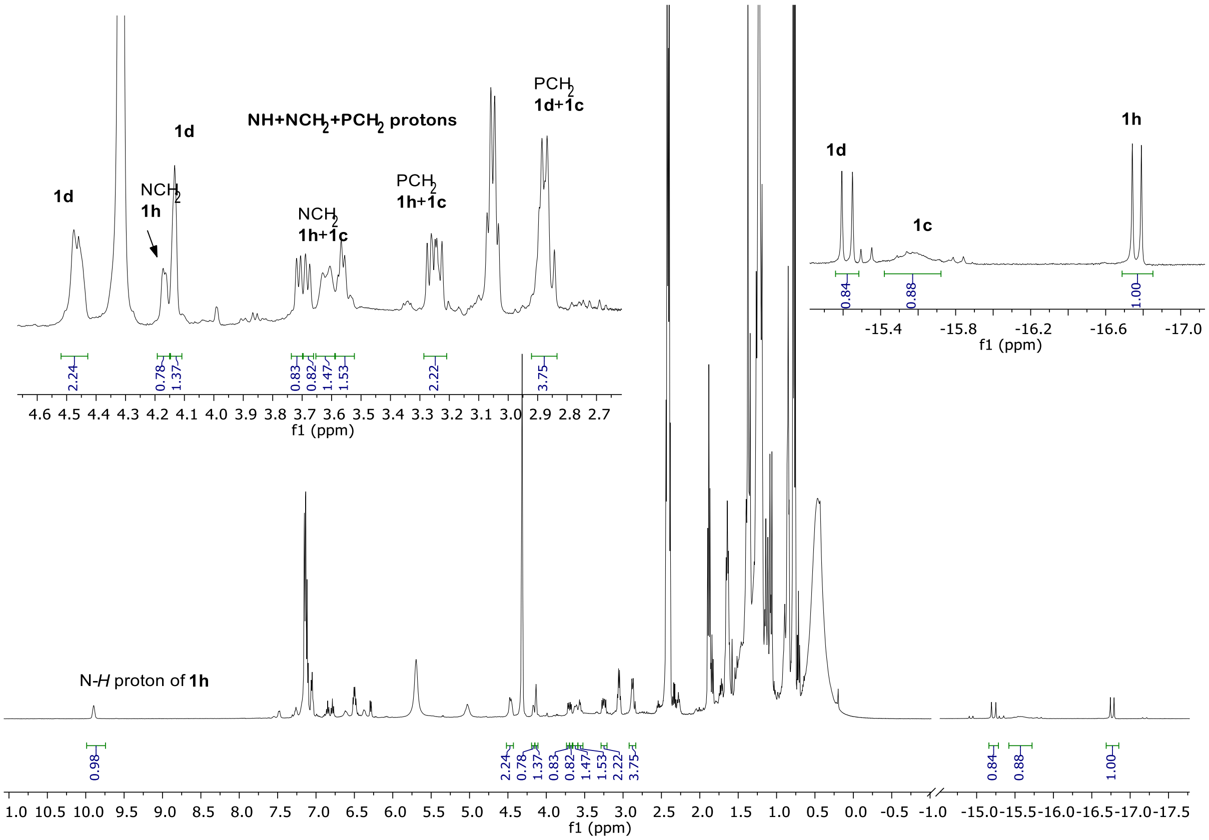

Figure S27. ^{1}H NMR spectrum of sample containing **1c**,**1d** and **1h** (**1c**/**1d**/**1h** ~ 6/6/7). The N*H* proton of **1h** in very downfield due to hydrogen bonding. N arm, P arm, and NH protons of the ligand are shown in left inset. Hydride region magnified in right inset.

6. X ray crystallography

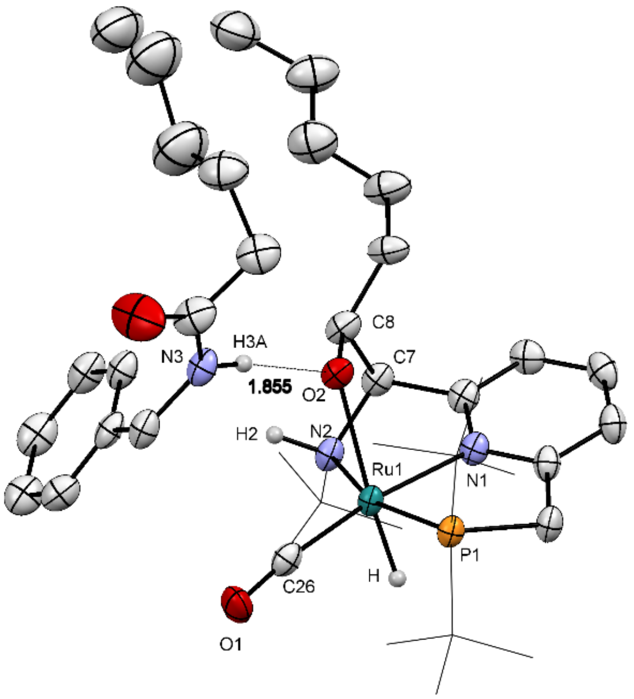

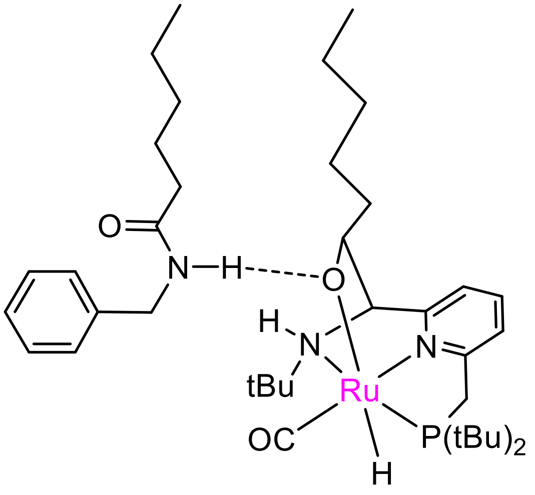

1d.$C_{13}H_{19}NO$

Key bond lengths (A) and angles (°): Ru(1)-P(1) 2.2576(5), Ru(1)-O(2) 2.2380(15), Ru(1)-N(1)2.0850(19),Ru(1)-N(2)2.2010(17), Ru(1)-C(26)(carbonyl) 1.818(2), O(2)-C(8) 1.394(3), C(7)-C(8) 1.570(3); P(1)-Ru(1)-H 81.5(11), O(2)-Ru(1)-P(1) 105.13(4), O(2)-Ru(1)-H 168.8(11), N(1)-Ru(1)-P(1) 81.81(5), N(1)-Ru(1)-O(2) 81.74(7), N(2)-Ru(1)-O(2) 72.92(6), C(8)-O(2)-Ru(1) 113.36(13), O(2)-C(8)-C(7) 109.38(18).

Data were collected on a Rigaku Xtalab Pro diffractometer dual source equipped with Dectris Pilatus 200K detector and microfocus, with CuKα (λ=1.54184 Å). The data were processed with CrysAlis^{PRO}. Structure was solved by direct methods with SHELXT.[8] Data were refined as Full-matrix least-squares refinement based on F^2 with SHELXL[9] and OLEX2[10]. All non-hydrogen atoms were further refined with anisotropic displacement coefficients. Hydrogen atoms were assigned isotropic displacement coefficients, and their coordinates were allowed to ride on their respective carbons. Hydride was located in the electron density map. Crystallographic data and refinement parameters are summarized in Supplementary Table S1.

Table S1. Crystal data and structure refinement for 1d.$C_{13}H_{19}NO$.

**1d.$C_{13}H_{19}NO$**		
**Empirical formula**	C_{39} H_{66} N_3 O_3 P Ru	
**Formula weight**	756.98 g/mol	
**Temperature**	100.01(10) K	
**Wavelength**	1.54184 A	
**Crystal system**	Monoclinic	
**Space group**	C*2/c*	
**Unit cell dimensions**	a = 38.7143(4) Å	α = 90°
	b = 11.06422(11) Å	β = 94.3975(8)°
	c = 18.64152(18)Å	γ = 90°
**Volume**	7961.47(14) A^3	
**Z**	8	
**Density (calculated)**	1.263 Mg/m^3	
**Absorption coefficient**	3.855 mm^{-1}	
**F(000)**	3232	
**Crystal size**	0.065 x 0.03 x 0.011 mm	
**Theta angle for data collection**	4.157 to 68.243 deg.	
**Limiting indices**	-45<=h<=46, -13<=k<=13, -21<=l<=22	
**Reflections collected/ unique**	58215 / 7298 [R(int) = 0.0654]	
**Completeness to theta =** 67.684	100.0 %	
**Absorption correction**	Gaussian	
**Max. and min, transmission**	1.000 and 0.926	
**Refinement method**	Full-matrix least-squares on F^2	
**Data/ restraints/ parameters**	7298 / 0 / 461	
**Goodness-of-fit on F^2**	1.053	
**Final R indices [I>2σ(I)]**	R1 = 0.0329, wR2 = 0.0870	
**R indices (all data)**	R1 = 0.0352, wR2 = 0.0884	
**Largest diff. peak and hole**	0.919 and -0.712 $e.A^{-3}$	

7. Procedure for the dehydrogenative synthesis of pharmaceuticals

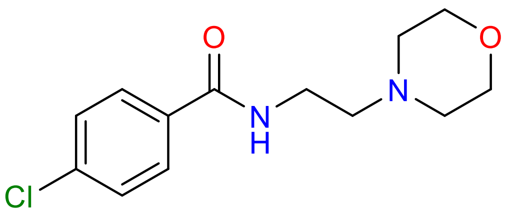

Moclobemide: 5 μmol of catalyst **1** and 10 μmol of *t*-BuOK were mixed in a 5 mL vial and 1 mL MTBE was added. The resulting brown solution was stirred for 5 min. In a separate 5 mL vial, 0.5 mmol of p-chlorobenzyl alcohol and 0.6 mmol of 4-(2-aminoethyl)morpholine (CAS# 2038-03-1) were dissolved in 1 mL MTBE. The latter solution was then added to the former brown solution at which the color disappeared to give a light red solution. The whole solution was then transferred to a 20 mL Schlenk flask with a side arm. The flask was sealed, taken outside the box, and connected to a condenser (1 ^{o}C, EG/H_2O). The solution was then refluxed for 60 h under argon flow (bath temperature 70 ^{o}C). After the given time, the flask was cooled down to room temperature, and a known amount of mesitylene was added to the solution as a standard. A portion of the solution was analyzed by ^{1}H NMR with $CDCl_3$ as the solvent to determine the NMR yield. The $CDCl_3$ solution was then added back to the parent solution and pure moclobemide was obtained from the crude solution by removing the solvent in vacuo, followed by recrystallization in a cold diethylether pentane solvent mixture (85%). Alternatively, moclobemide can also be purified by flash column chromatography (MeOH 1-2% in CH_2Cl_2) and obtained as a white powder (88% yield).

^{1}H NMR (400 MHz, $CDCl_3$) δ 7.69 (d, *J* = 8.5 Hz, 2H), 7.38 (d, *J* = 8.4 Hz, 2H), 6.83 (t, *J* = 5.1 Hz, 1H), 3.70 (t, *J* = 4.6 Hz, 4H), 3.51 (q, *J* = 5.7 Hz, 2H), 2.57 (t, *J* = 6.1 Hz, 2H), 2.47 (s, 4H).
^{13}C{^{1}H} NMR (101 MHz, $CDCl_3$) δ 166.35, 137.60, 132.98, 128.80, 128.38, 66.98, 56.85, 53.34, 36.14.

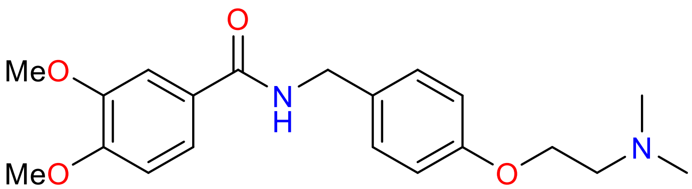

Itopride: 5 μmol of catalyst **1** and 10 μmol of *t*-BuOK were mixed in a 5 mL vial and 1 mL MTBE was added. The resulting dark brown solution was stirred for 5 min. In a separate 5 mL vial, 0.5 mmol of 3,4-dimethoxybenzyl alcohol and 0.6 mmol of 4-[2-(dimethylamino)ethoxy]benzylamine (CAS# 20059-73-8) were dissolved in 1 mL MTBE. The latter solution was then added to the former dark brown solution at which the brown color disappeared to give a light red solution. The whole solution was then transferred to a 20 mL Schlenk flask with a side arm. The flask was sealed, taken outside the box, and connected to a condenser (1 ^{o}C, EG/H_2O). The solution was then refluxed for 60 h under argon flow (bath temperature 70 ^{o}C). After the given time, the flask was cooled down to room temperature, and a known amount of mesitylene was added to the solution as a standard. A portion of the solution was analyzed by ^{1}H NMR with $CDCl_3$ as the deuterated solvent to determine the NMR yield. The NMR solution was then added back to the parent solution and pentane was added

to the solution. Formation of a white precipitate was observed which was filtered, washed with copious amounts of pentane and diethyl ether and dried to afford pure itopride in 92% yield.

^{1}H NMR (500 MHz, $CDCl_3$) δ 7.53 (d, *J* = 1.8 Hz, 1H), 7.39 (dd, *J* = 8.3, 1.9 Hz, 1H), 7.33 (d, *J* = 8.5 Hz, 2H), 6.96 (d, *J* = 8.6 Hz, 2H), 6.90 (d, *J* = 8.4 Hz, 1H), 6.77 (t, *J* = 5.1 Hz, 1H), 4.62 (d, *J* = 5.6 Hz, 2H), 4.12 (t, *J* = 5.7 Hz, 2H), 3.97 (d, *J* = 1.7 Hz, 6H), 2.80 (t, *J* = 5.7 Hz, 2H), 2.41 (s, 6H).
^{13}C NMR (126 MHz, $CDCl_3$) δ 166.94, 158.26, 151.69, 148.92, 130.61, 129.20, 127.06, 119.47, 114.73, 110.68, 110.23, 65.98, 58.23, 55.97, 55.96, 45.84, 43.57.

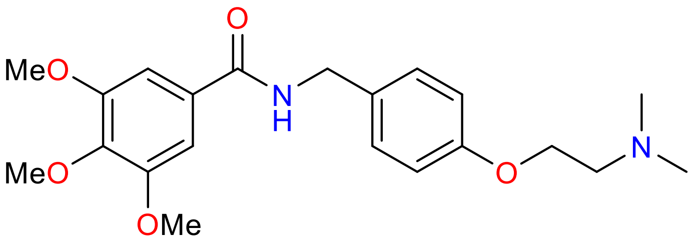

Trimethobenzamide: 5 µmol of catalyst **1** and 10 µmol of *t*-BuOK were mixed in a 5 mL vial and 1 mL MTBE was added. The resulting dark brown solution was stirred for 5 min. In a separate 5 mL vial. 0.5 mmol of 3,4,5-trimethoxybenzyl alcohol and 0.6 mmol of 4-[2-(dimethylamino)ethoxy]benzylamine (CAS# 20059-73-8) were dissolved in 1 mL MTBE. The latter solution was then added to the former dark brown solution at which the brown color disappeared to give a light red solution. The whole solution was then transferred to a 20 mL Schlenk flask with a side arm. The flask was sealed, taken outside the box, and connected to a condenser (1 ^{o}C, EG/H_2O). The solution was then refluxed for 60 h under argon flow (bath temperature 70 ^{o}C). After the given time, the flask was cooled down to room temperature, and pentane was added to the solution. Formation of white precipitate was observed which was filtered, washed with copious amounts of pentane and diethyl ether, and dried to afford pure trimethobenzamide in 94% yield.

^{1}H NMR (300 MHz, $CDCl_3$) δ 7.26 (d, *J* = 8.1 Hz, 2H), 7.05 (s, 2H), 6.89 (d, *J* = 8.3 Hz, 2H), 6.74 (d, *J* = 5.8 Hz, 1H), 4.54 (d, *J* = 5.6 Hz, 2H), 4.06 (t, *J* = 5.7 Hz, 2H), 3.87 (s, 3H), 3.86 (s, 6H), 2.73 (t, *J* = 5.7 Hz, 2H), 2.34 (s, 6H).
^{13}C NMR (75 MHz, $CDCl_3$) δ 167.02, 158.32, 153.12, 140.83, 130.43, 129.85, 129.23, 114.73, 104.44, 66.01, 60.87, 58.25, 56.26, 45.87, 43.65.

N, N-Diethyl-meta-toluamide (DEET): 10 µmol of catalyst **1** and 20 µmol of t-BuOK were mixed in a 5 mL vial and 1 mL dioxane was added. The resulting violet solution was stirred for 5 min. In a separate 5 mL vial 0.5 mmol of 3-methylbenzyl alcohol and 5 mmol of diethylamine were dissolved in 1 mL dioxane. The latter solution was then added to the former violet solution at which the violet color disappeared to give a light red solution. The resulting solution was then transferred to a 50 mL Schlenk tube. 0.5 mmol of powdered K_3PO_4 was subsequently added to the reaction flask. Afterwards, the tube was sealed and heated at a bath temperature of 130 ^{o}C for 60 h. The generated gas mixture was released in the 6th, 28th and 50th hour. After the given time, the reaction mixture was cooled down to room temperature and filtered via a short Celite pad to remove the K_3PO_4 affording a light yellow color solution. A known amount of mesitylene was added to the solution and a part of the solution was analyzed by ^{1}H NMR with $CDCl_3$ as the deuterated solvent to determine the NMR yield (32%). The product was not isolated due to low yield.

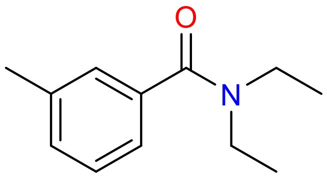

8. ^{1}H and ^{13}C{^{1}H} spectra of isolated compounds

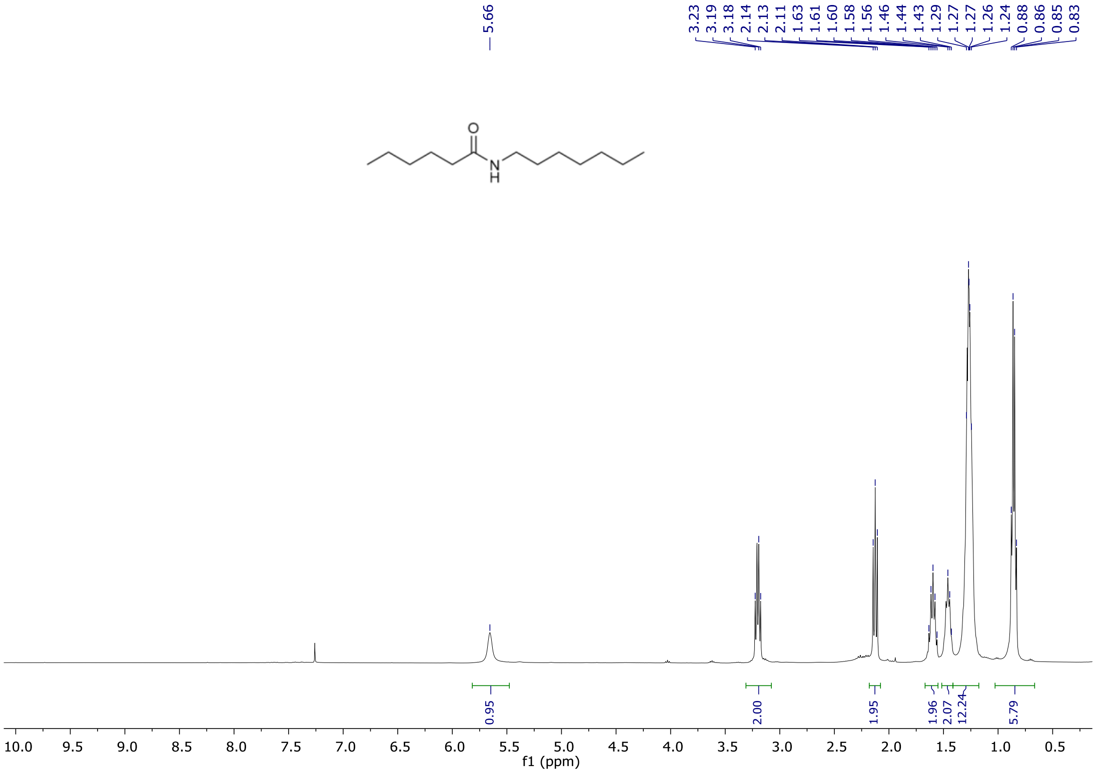

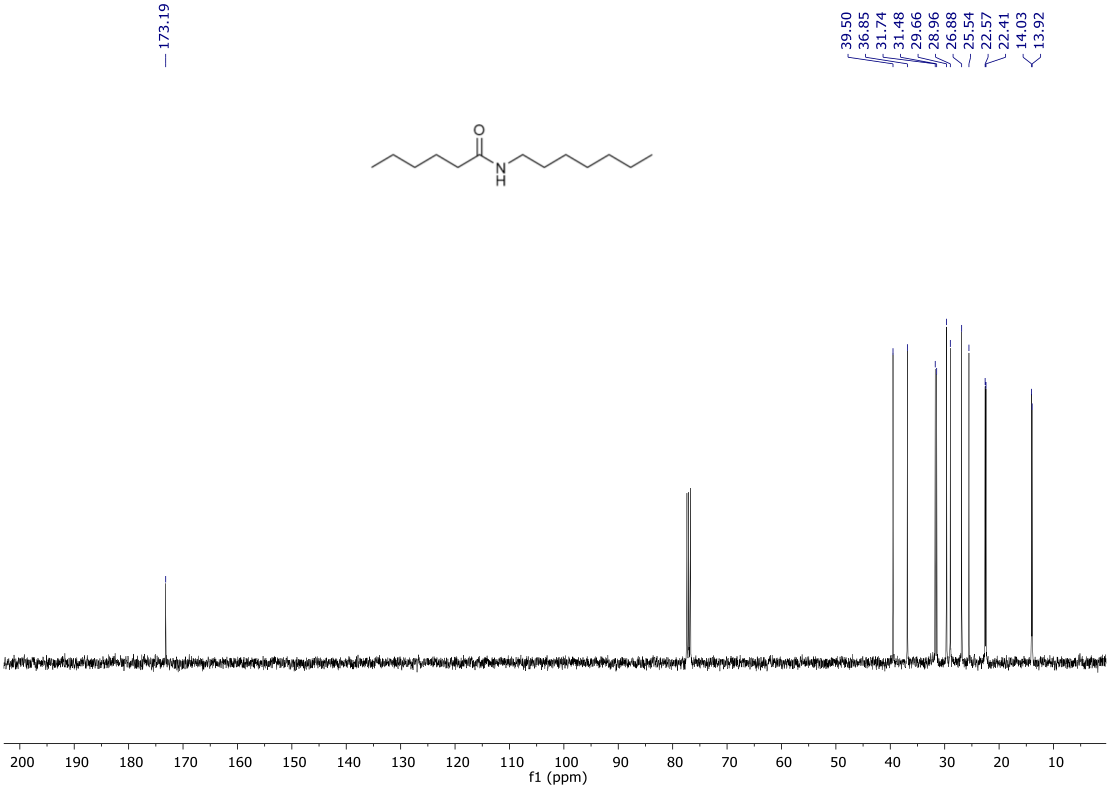

Figure S28. ^{1}H (top, 400 MHz) and ^{13}C{^{1}H} (bottom, 101 MHz) NMR spectra of isolated N-heptylhexanamide (T2, E1) in $CDCl_3$.

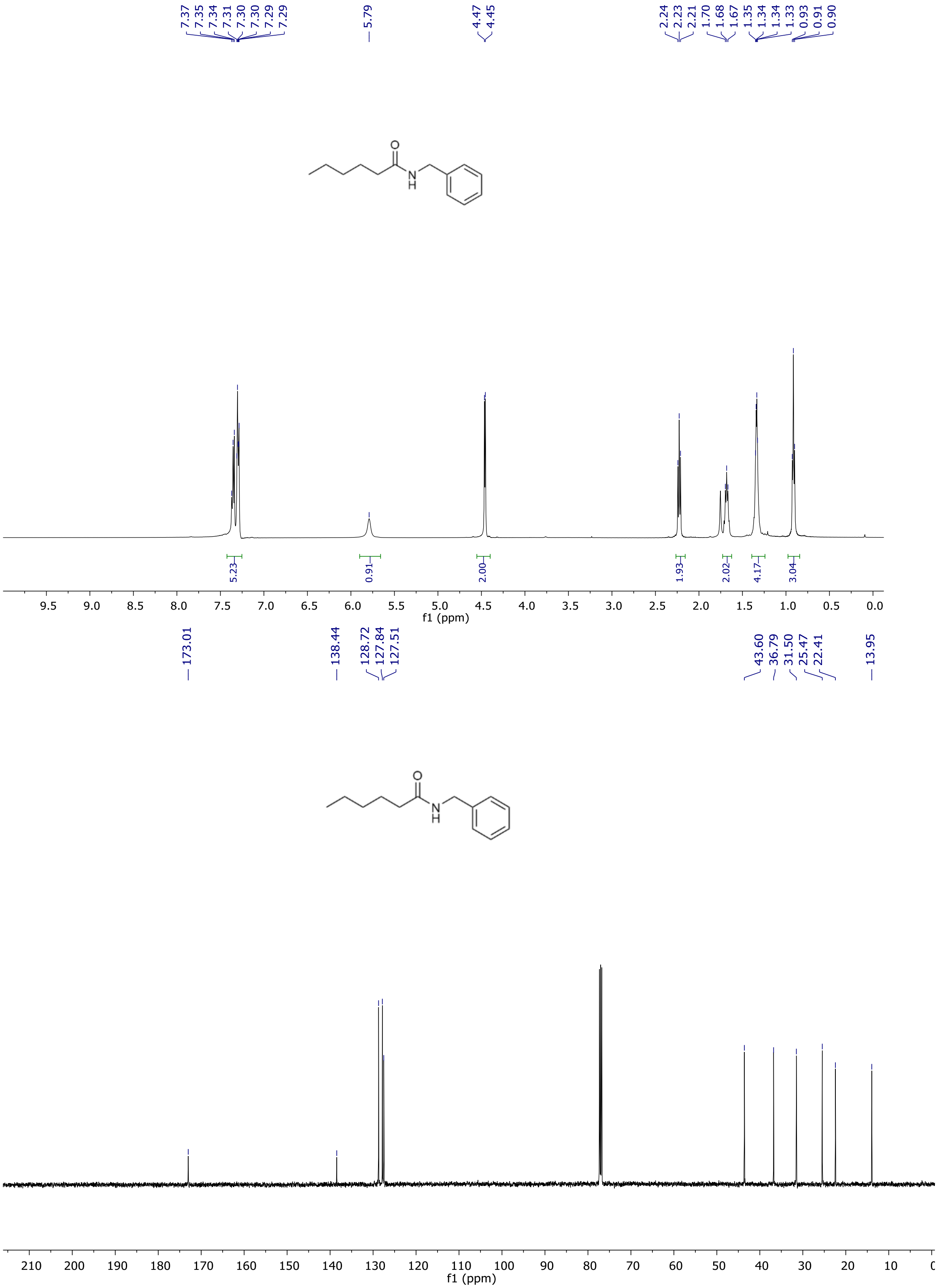

Figure S29. ^{1}H (top, 500 MHz) and $^{13}C\{^{1}H\}$ (bottom, 125 MHz) NMR spectra of isolated N-benzylhexanamide (T2, E2) in $CDCl_3$.

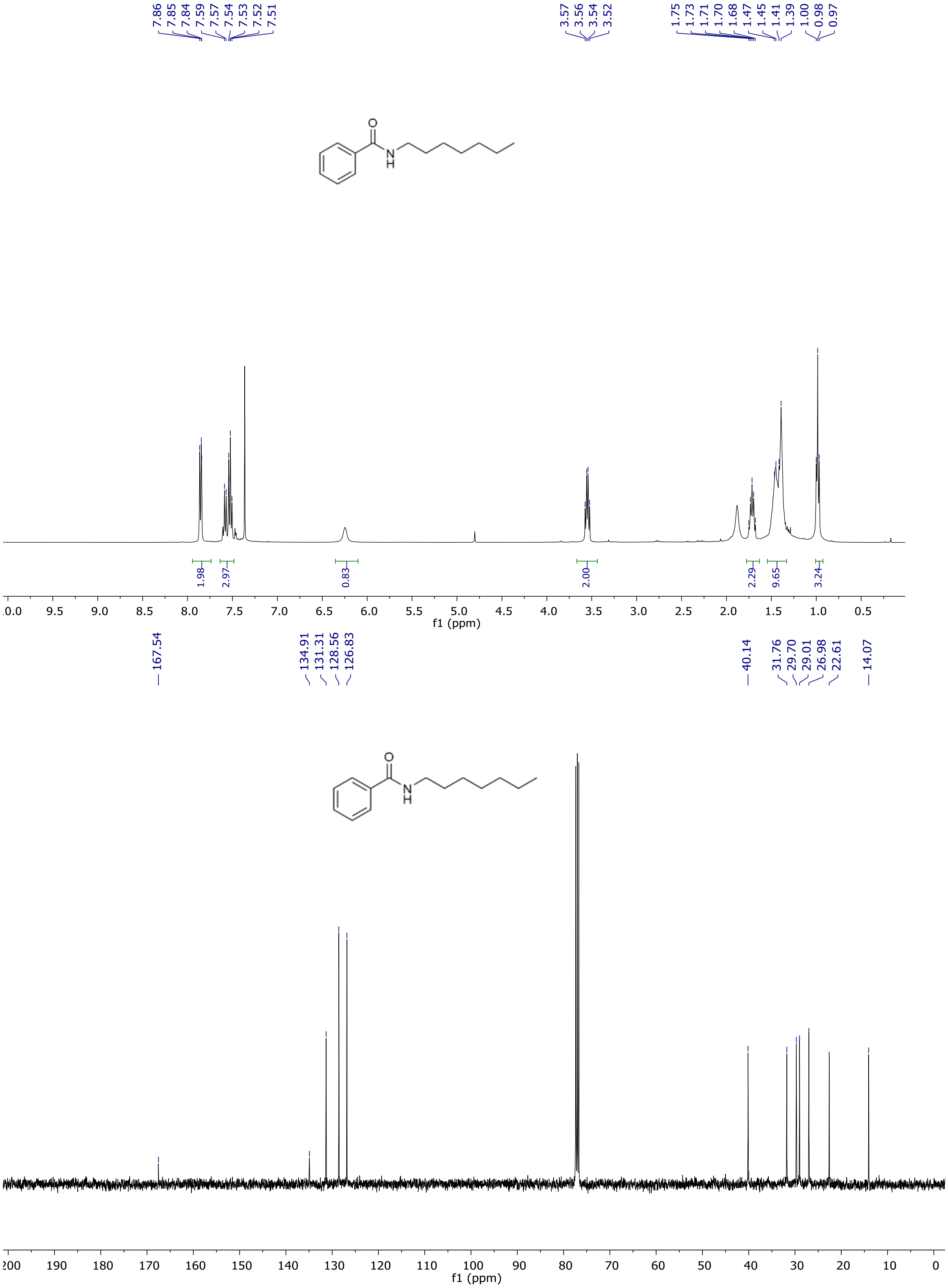

Figure S30. ^{1}H (top, 400 MHz) and $^{13}C\{^{1}H\}$ (bottom, 101 MHz) NMR spectra of isolated N-heptylbenzamide (T2, E3) in $CDCl_3$.

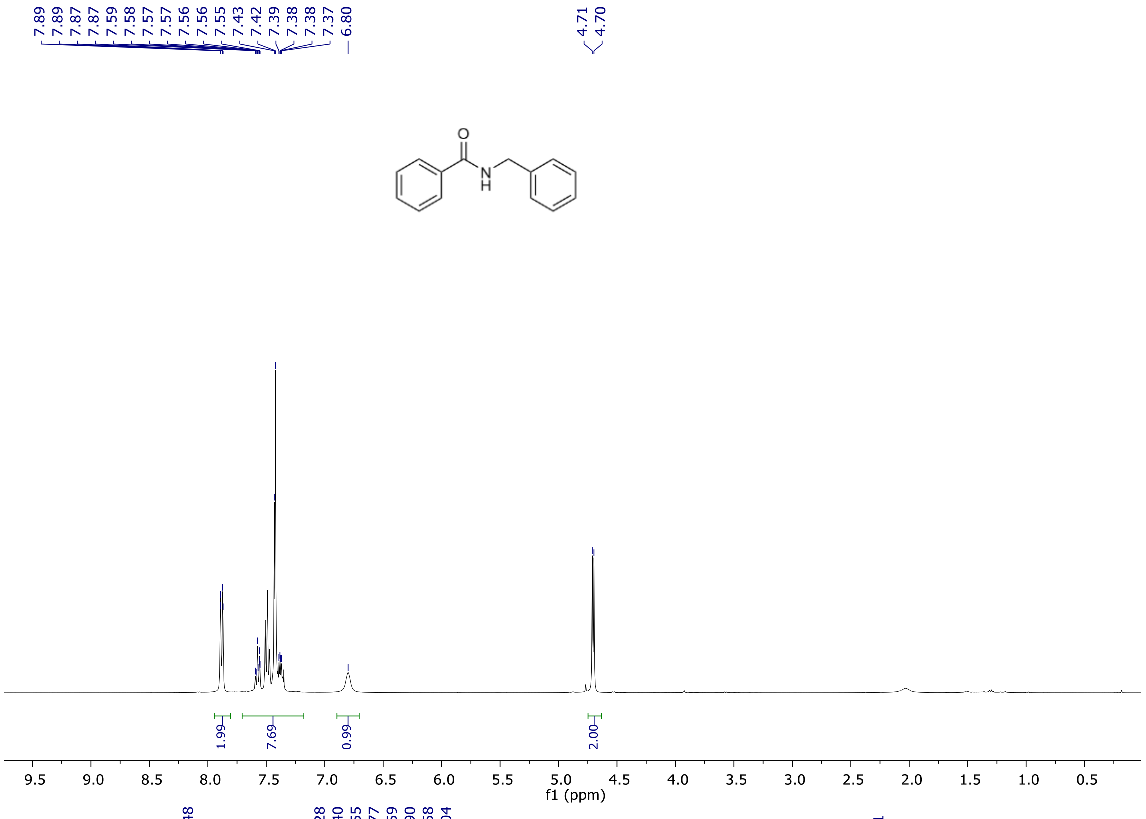

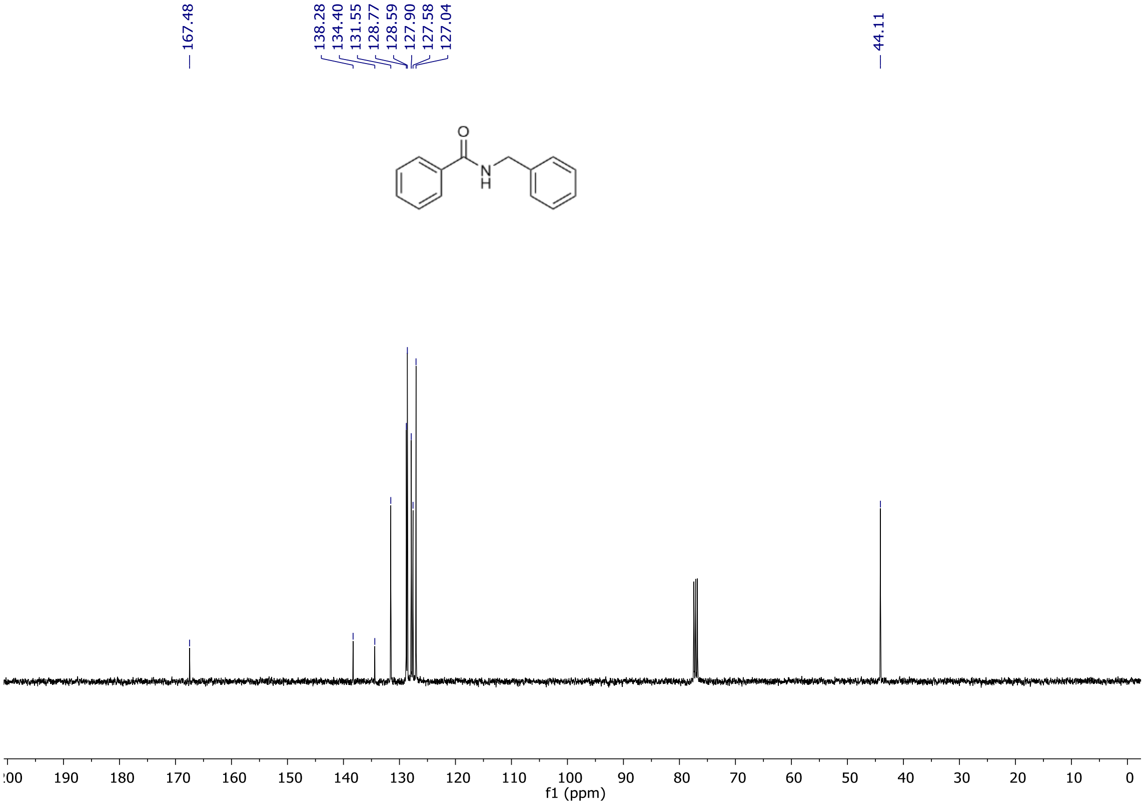

Figure S31. ^{1}H (top, 400 MHz) and $^{13}C\{^{1}H\}$ (bottom, 101 MHz) NMR spectra of isolated N-benzylbenzamide (T2, E4) in $CDCl_3$.

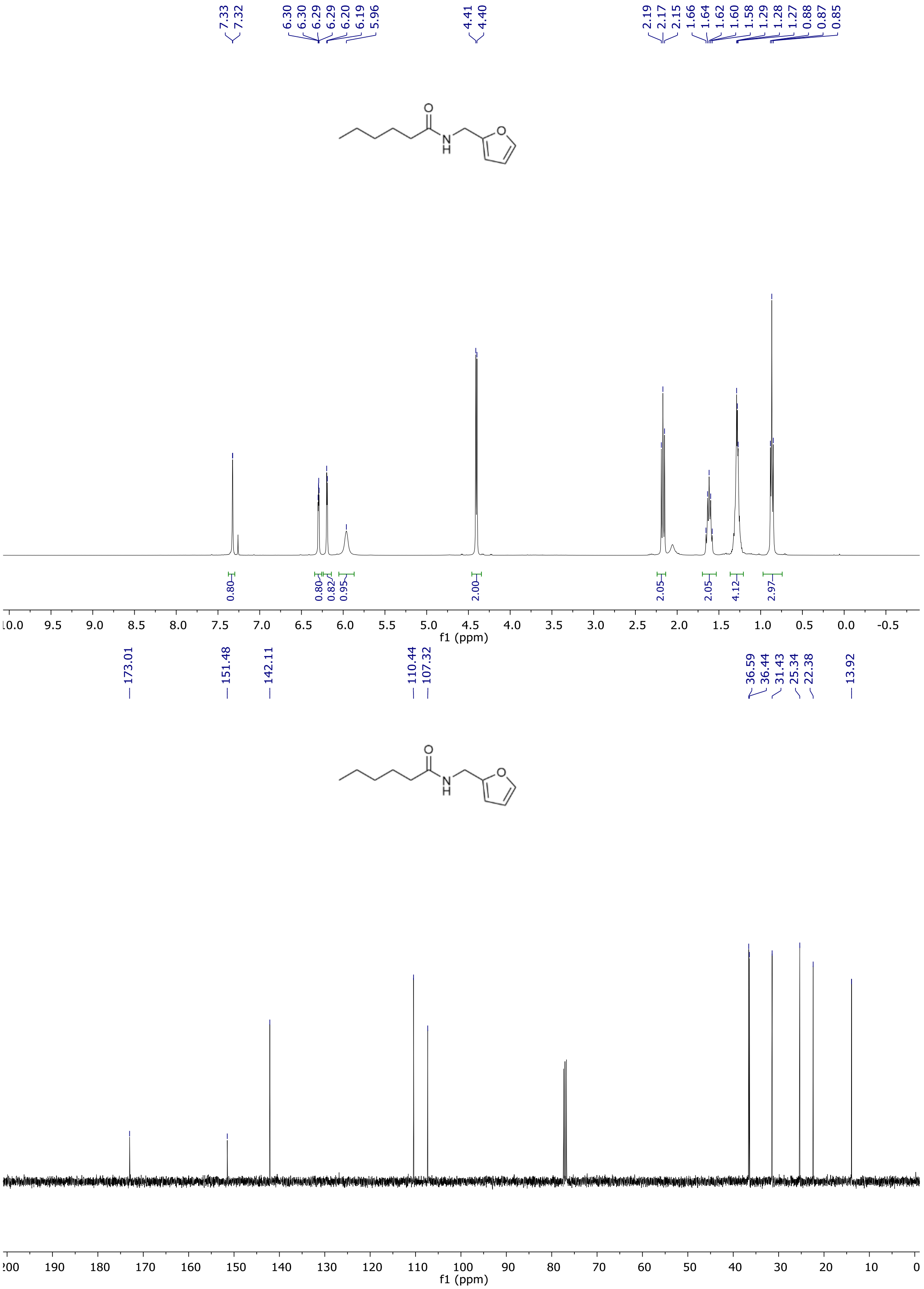

Figure S32. ^{1}H (top, 400 MHz) and $^{13}C\{^{1}H\}$ (bottom, 101 MHz) NMR spectra of isolated N-furfurylhexanamide (T2, E5) in $CDCl_3$.

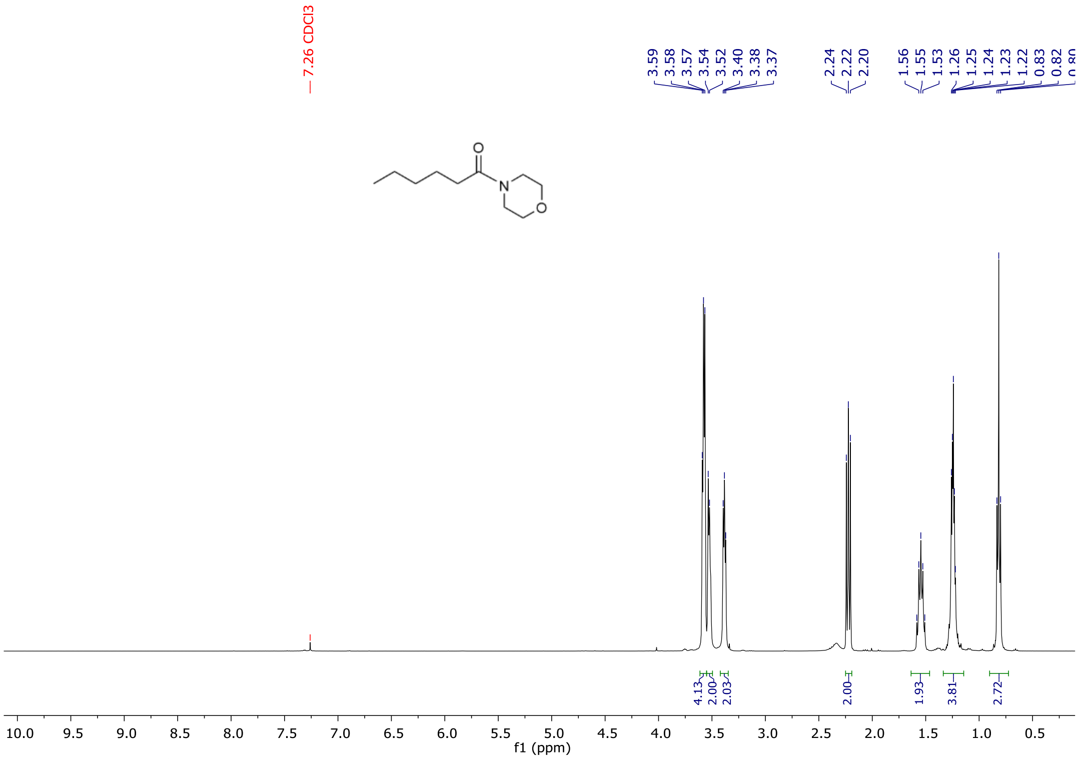

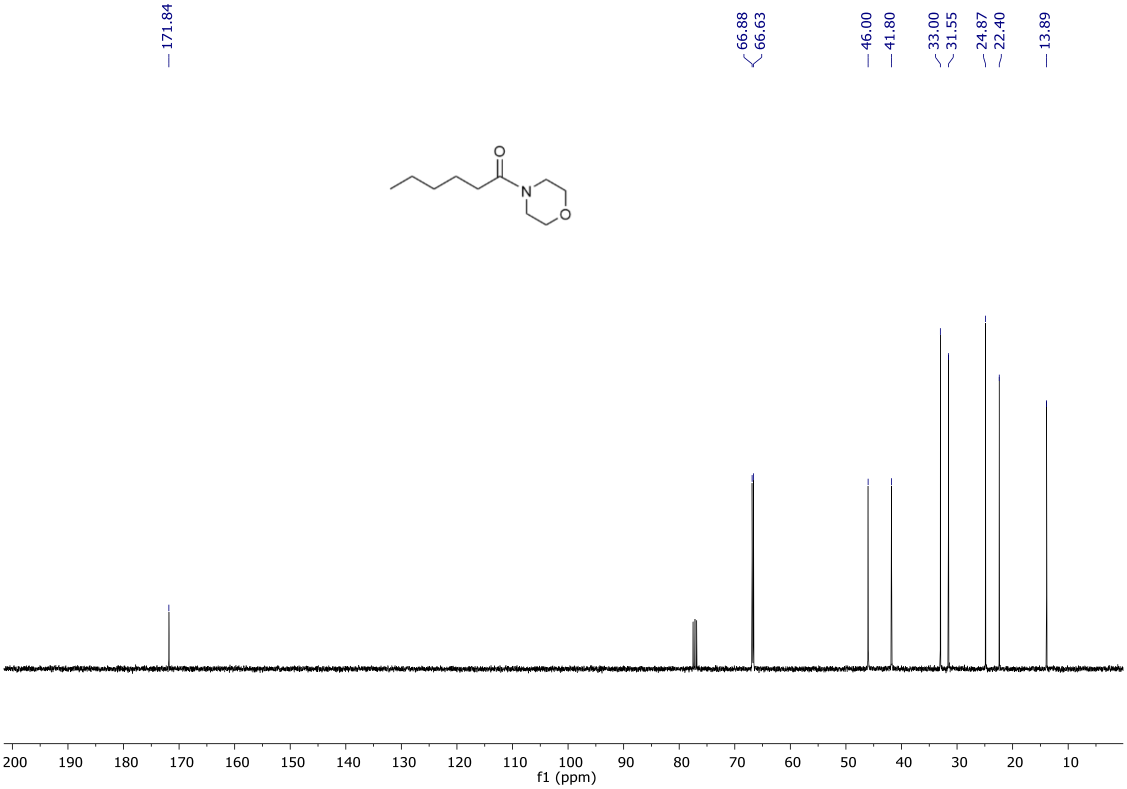

Figure S33. ^{1}H (top, 400 MHz) and $^{13}C\{^{1}H\}$ (bottom, 101 MHz) NMR spectra of isolated N-hexanoylmorpholine (T2, E7) in $CDCl_3$.

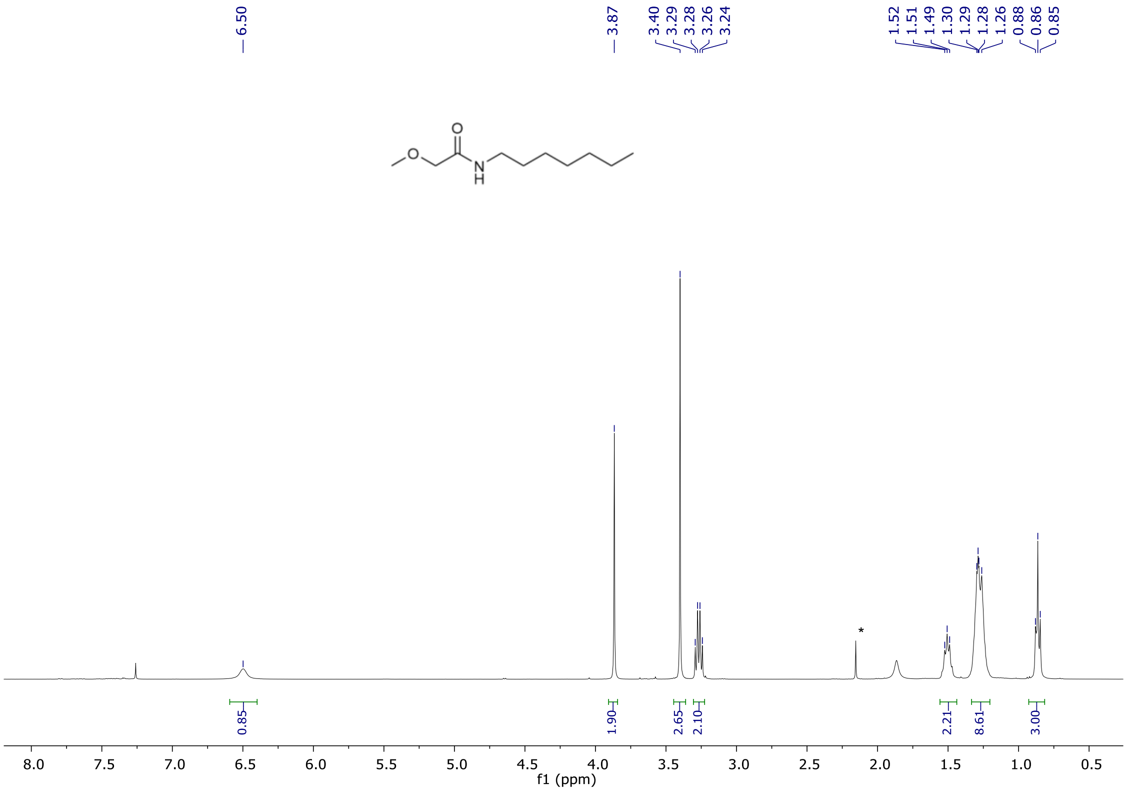

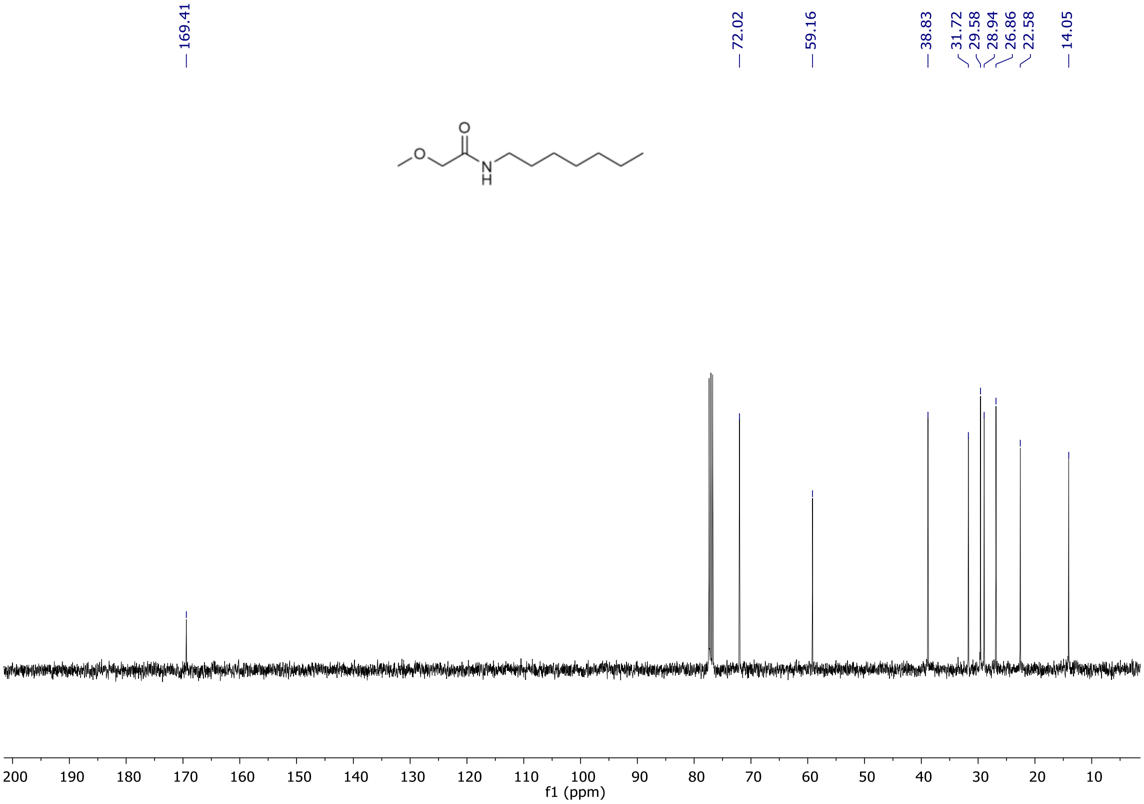

Figure S34. ^{1}H (top, 400 MHz) and $^{13}C\{^{1}H\}$ (bottom, 101 MHz) NMR spectra of isolated N-heptyl-methoxyacetamide (T2, E11) in $CDCl_3$.

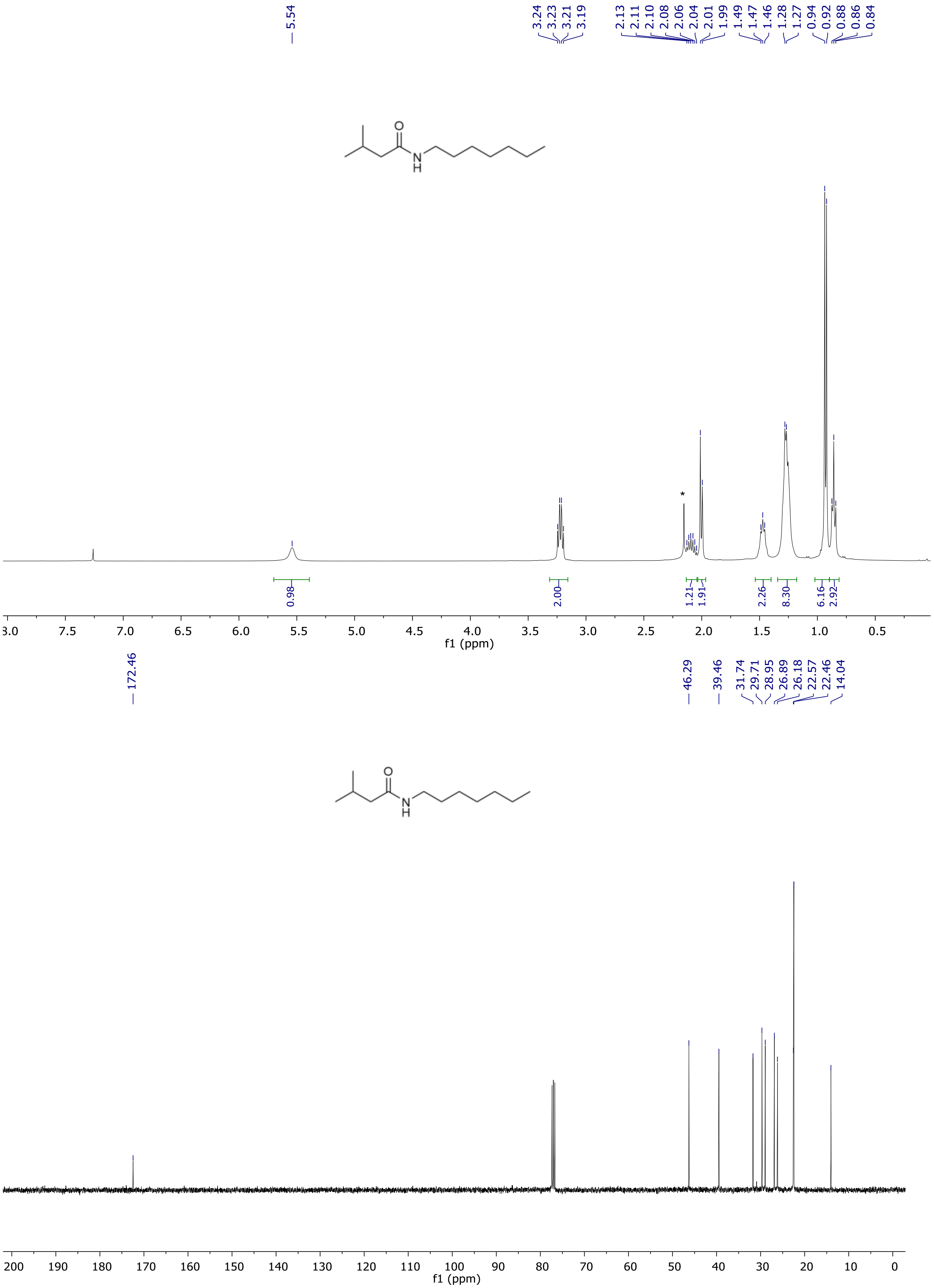

Figure S35. ^{1}H (top, 400 MHz) and $^{13}C\{^{1}H\}$ (bottom, 101 MHz) NMR spectra of isolated N-heptyl-isovaleramide (T2, E13) in $CDCl_3$. *acetone from the NMR tube.

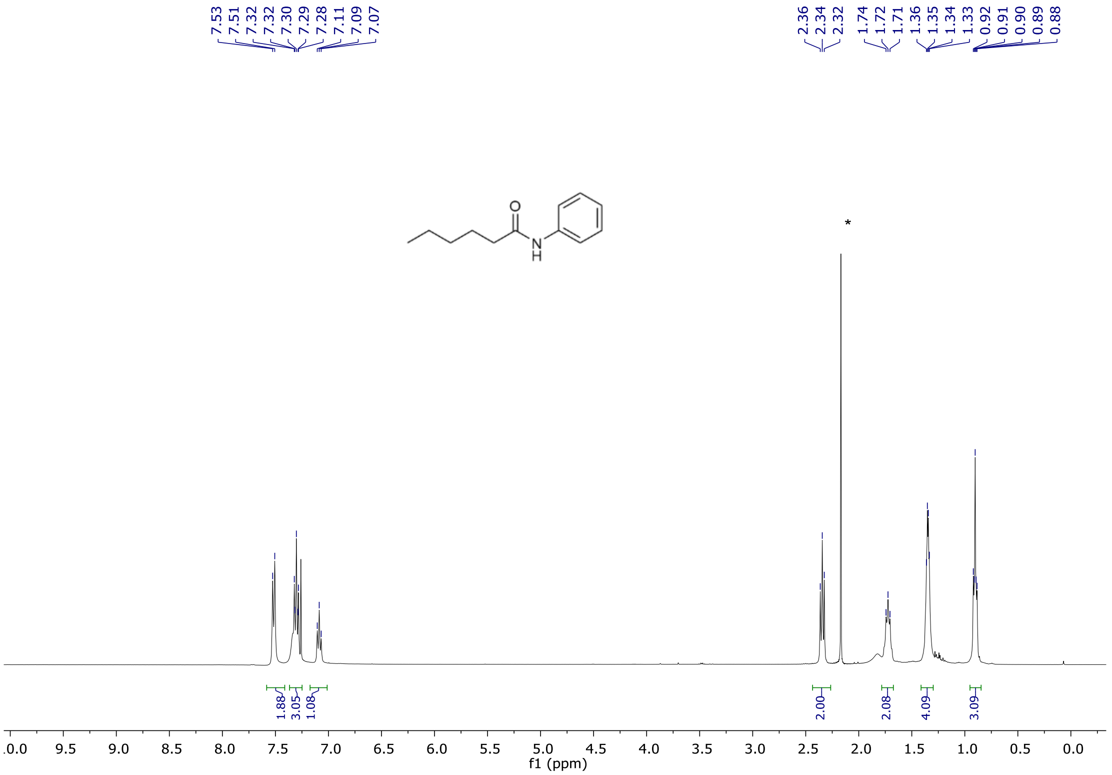

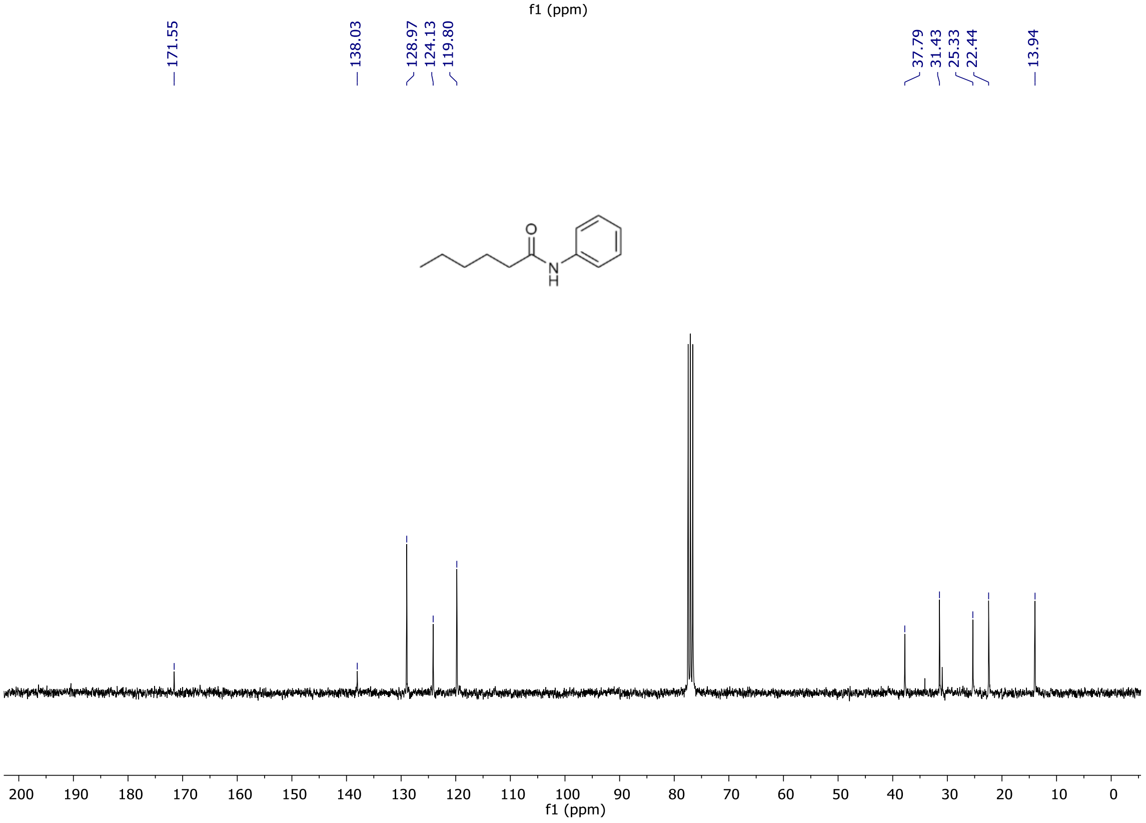

Figure S36. ^{1}H (top, 400 MHz) and $^{13}C\{^{1}H\}$ (bottom, 101 MHz) NMR spectra of isolated N-phenylhexanamide (T2, E16) in $CDCl_3$. *acetone from the NMR tube.

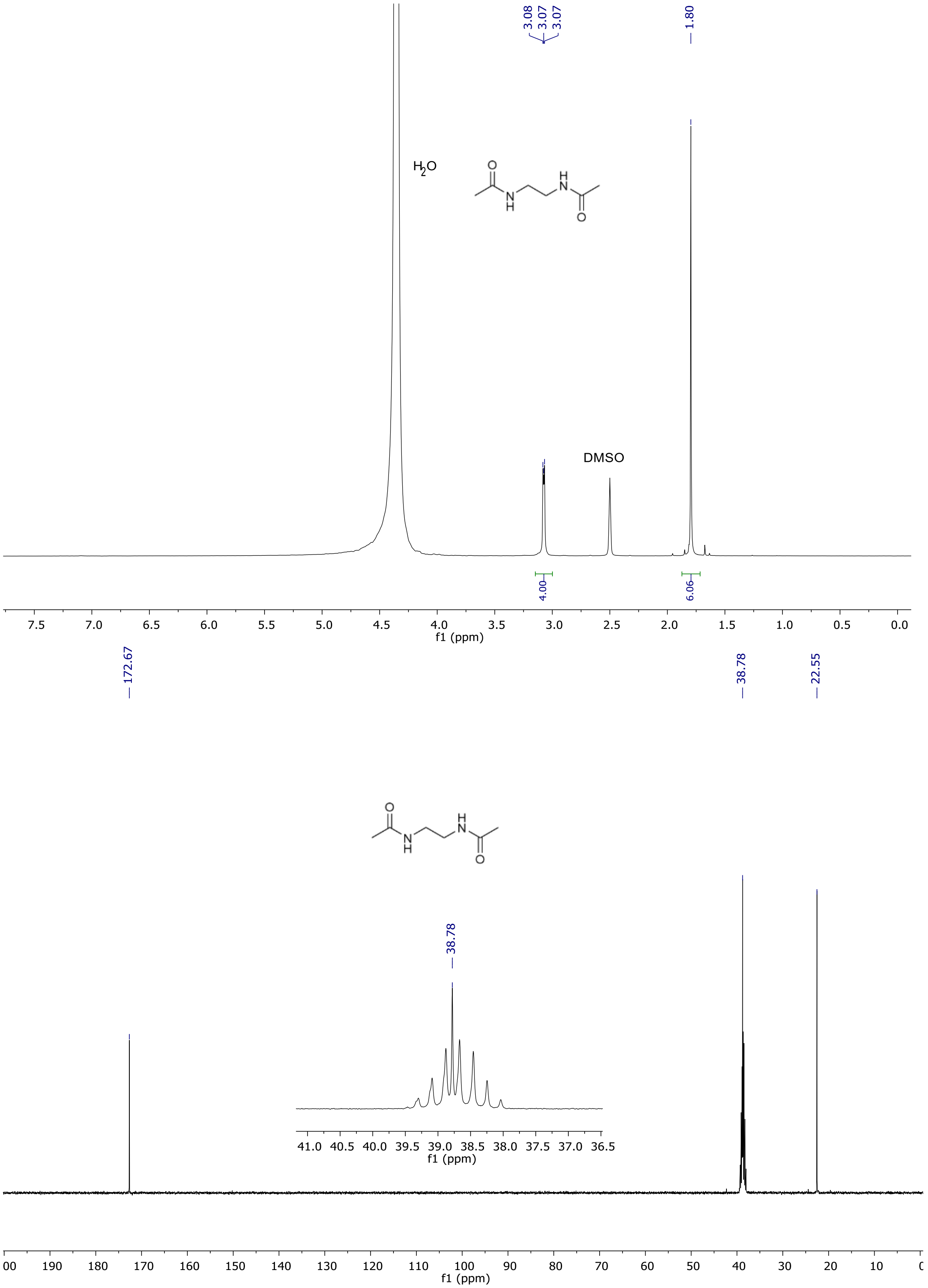

Figure S37. ^{1}H (top, 400 MHz) and $^{13}C\{^{1}H\}$ (bottom, 101 MHz) NMR spectra of isolated N,N'-Diacetylethylenediamine (T2, E18) in DMSO-d_6.

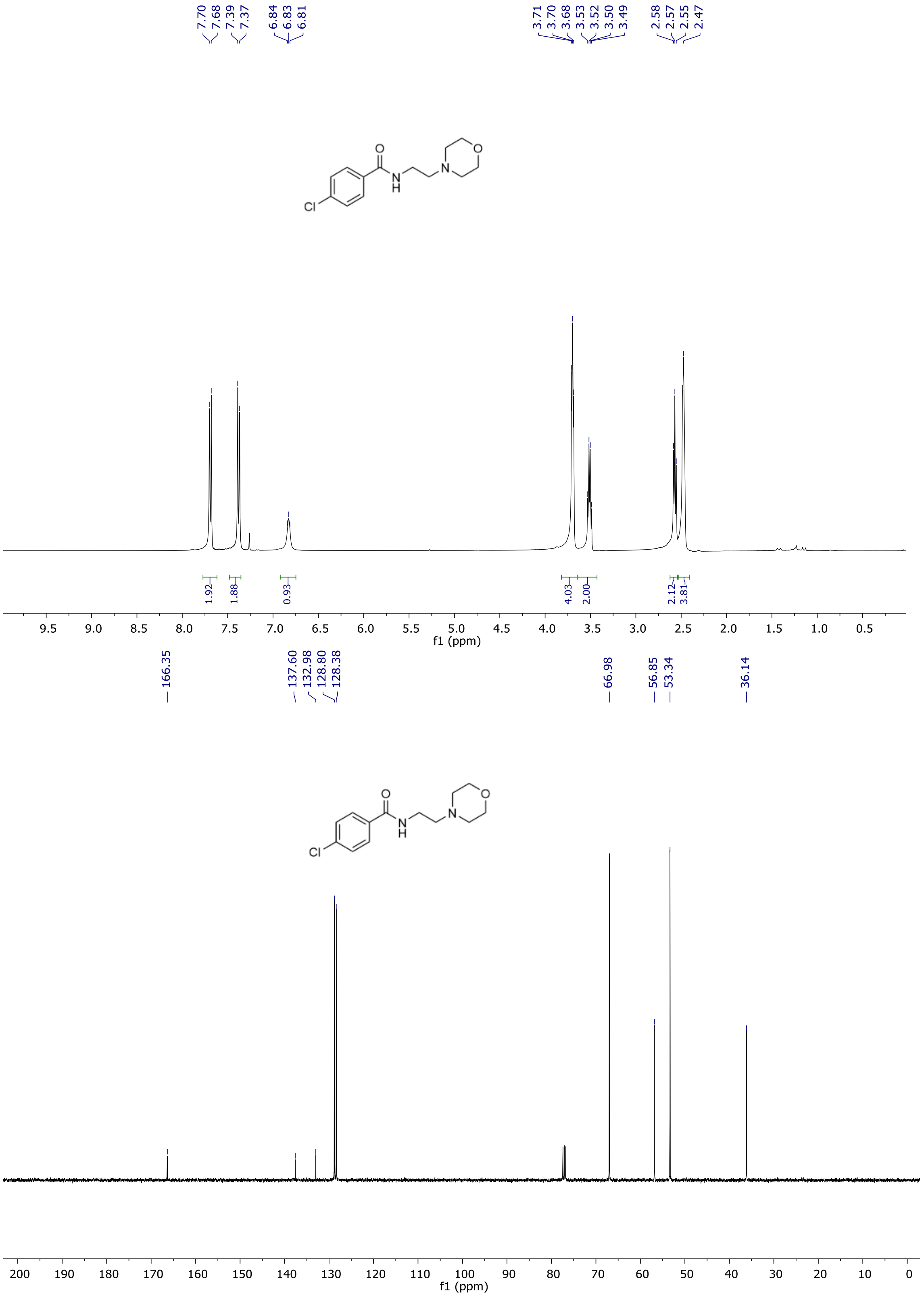

Figure S38. ^{1}H (top, 400 MHz) and ^{13}C{^{1}H} (bottom, 101 MHz) NMR spectra of isolated moclobemide (Scheme 6) in $CDCl_3$.

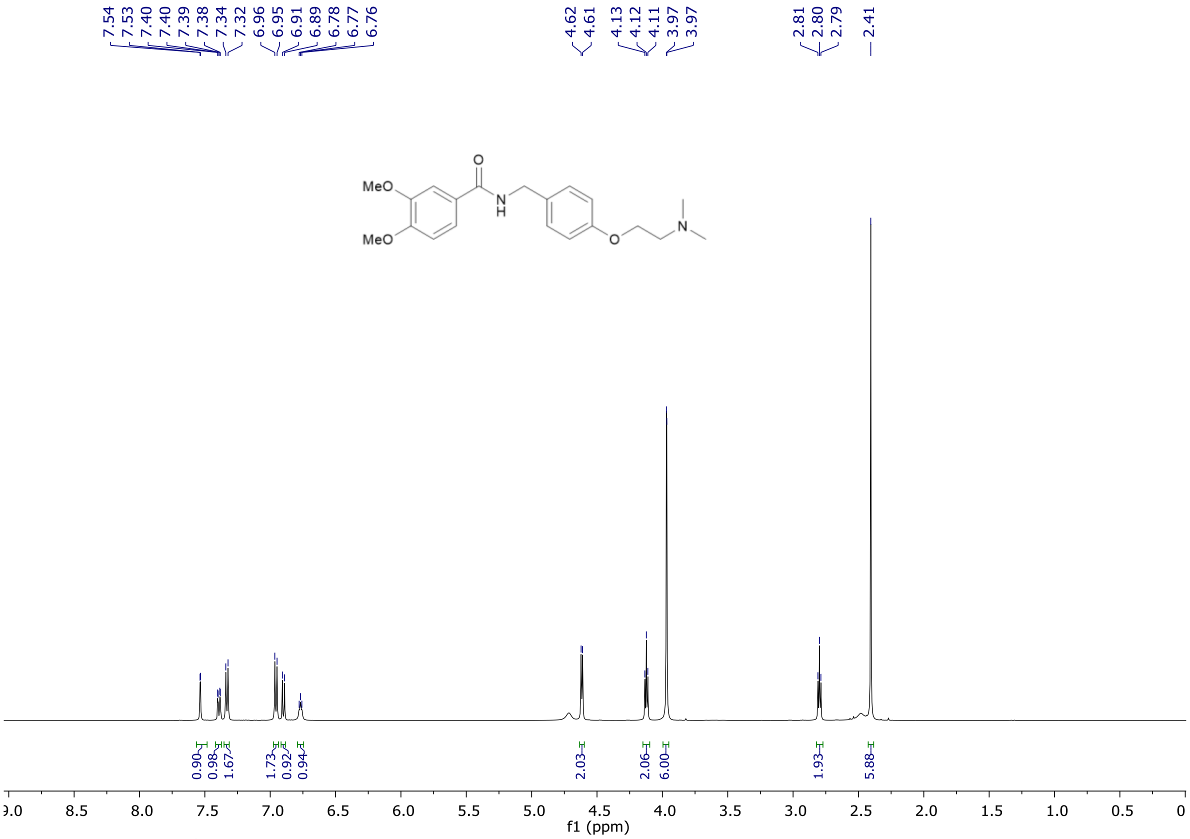

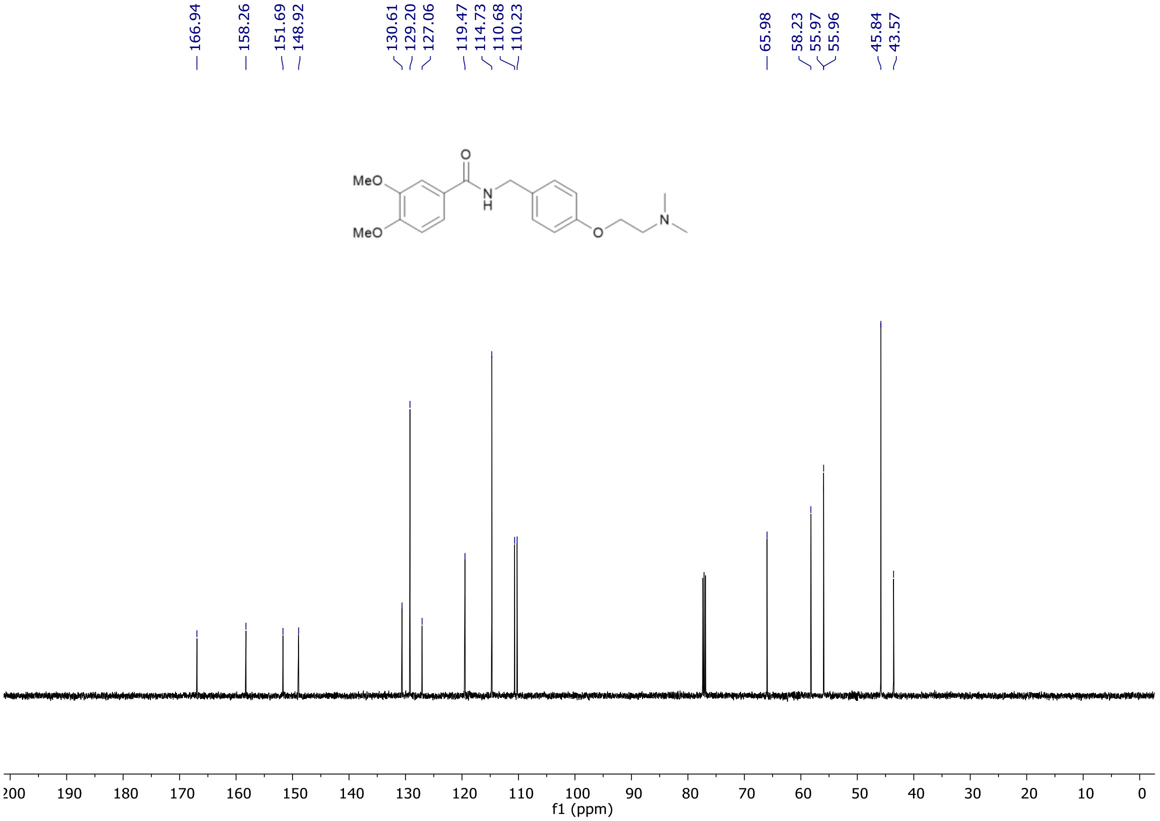

Figure S39. ^{1}H (top, 500 MHz) and $^{13}C\{^{1}H\}$ (bottom, 126 MHz) NMR spectra of isolated itopride (Scheme 6) in $CDCl_3$.

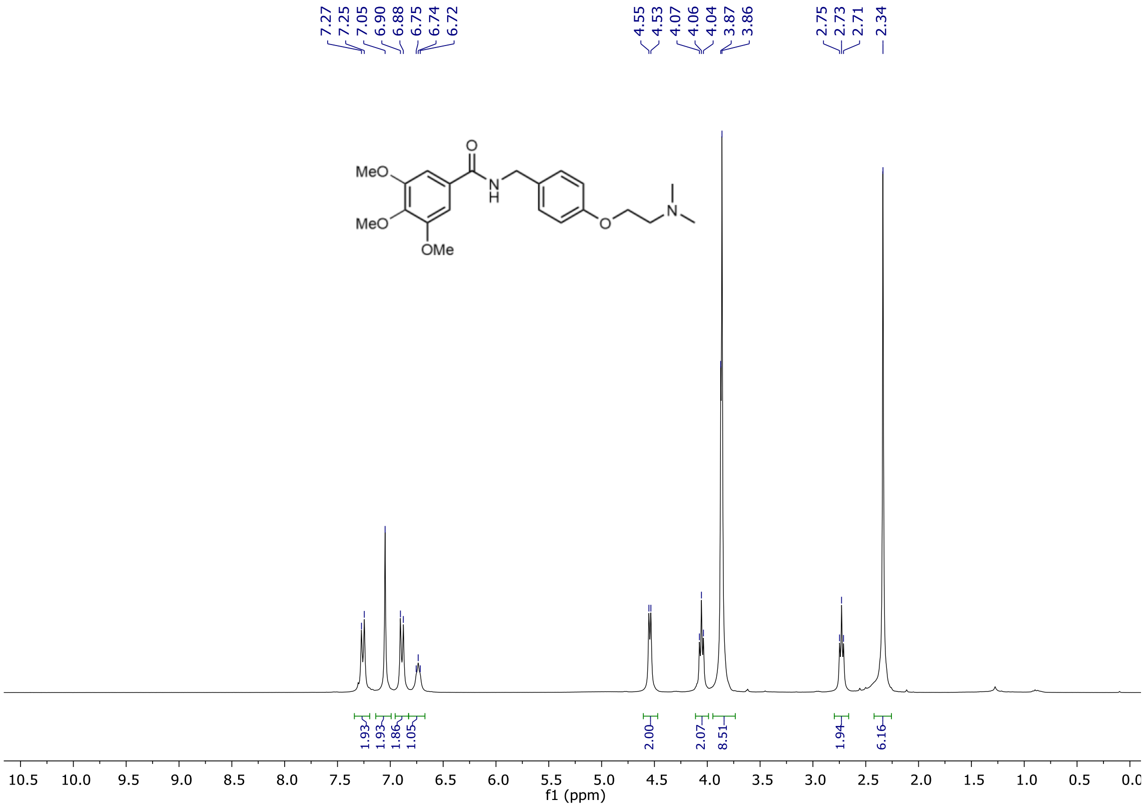

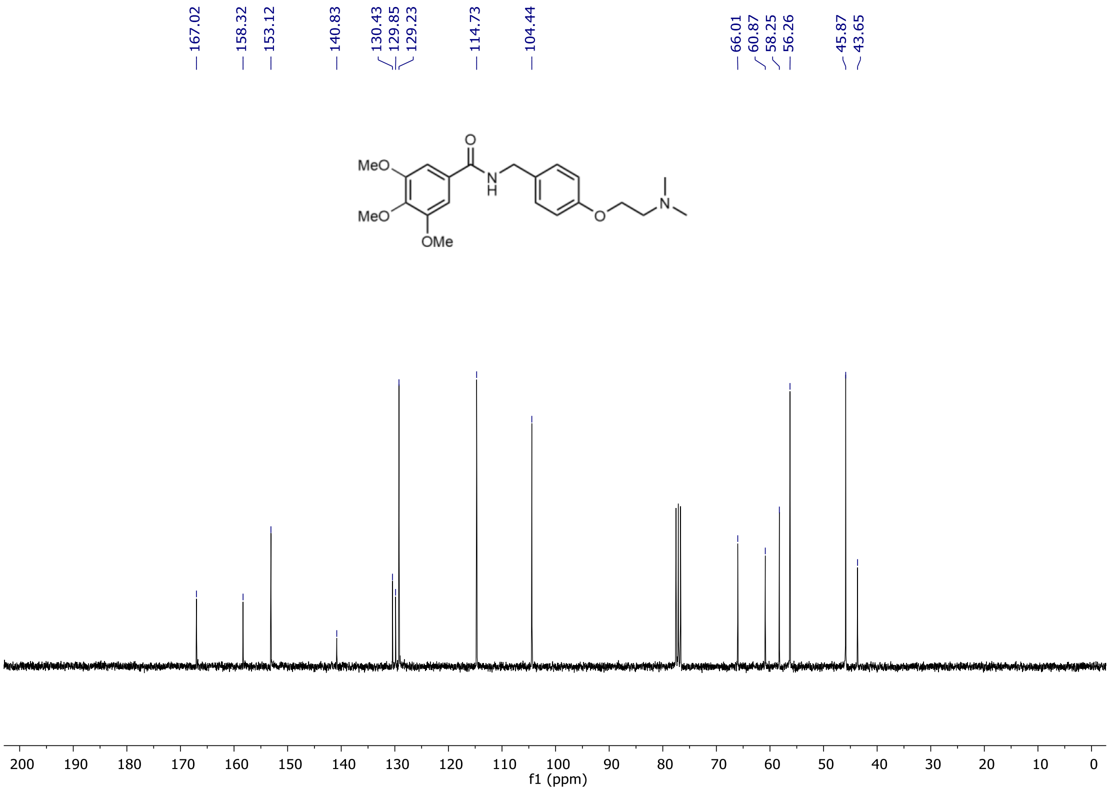

Figure S40. ^{1}H (top, 300 MHz) and $^{13}C\{^{1}H\}$ (bottom, 75 MHz) NMR spectra of isolated trimethobenzamide (Scheme 6) in $CDCl_3$.

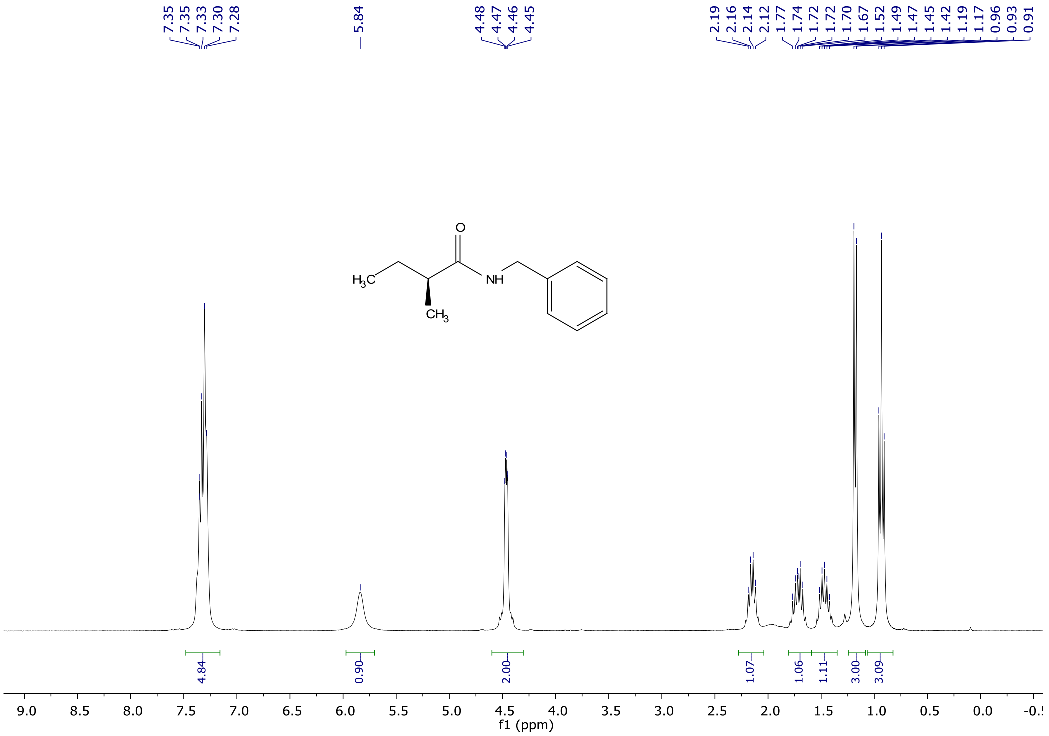

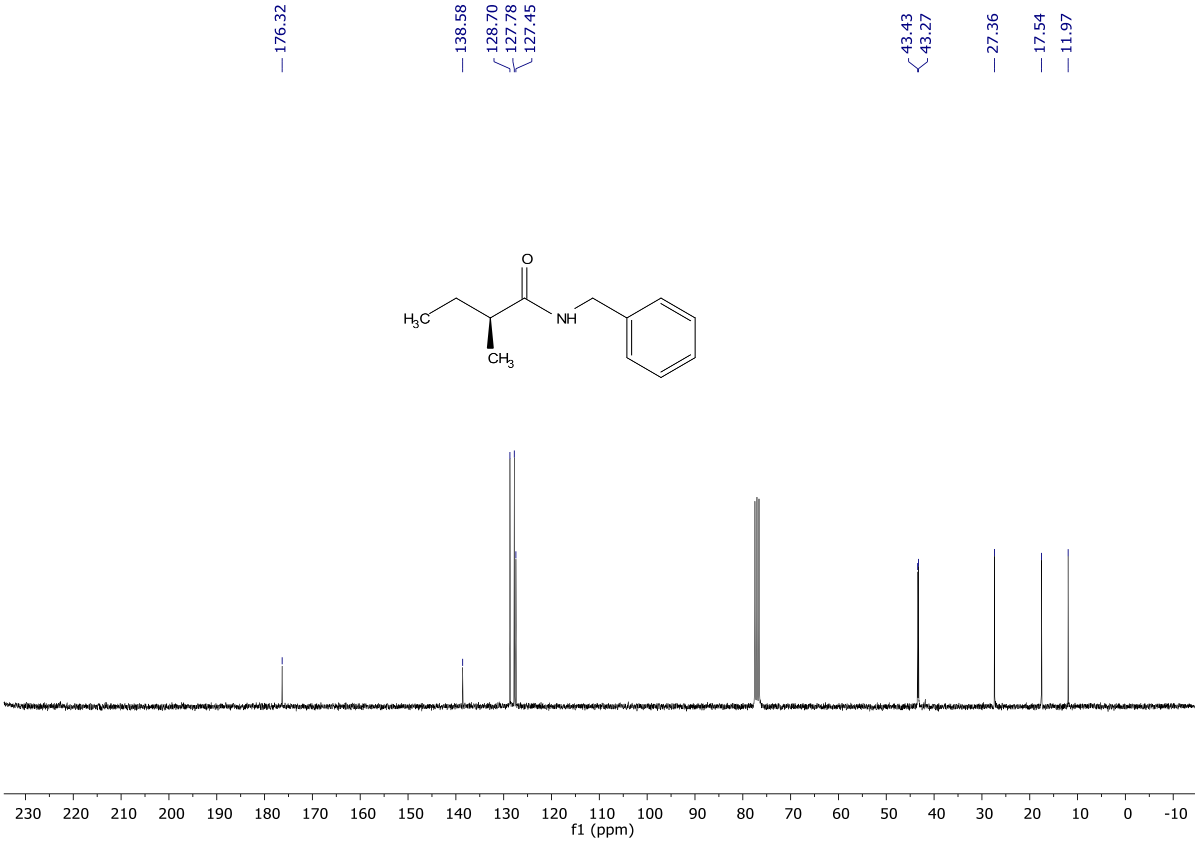

Figure S41. ^{1}H (top, 300 MHz) and ^{13}C{^{1}H} (bottom, 75 MHz) NMR spectra of isolated (*S*)-N-benzyl-2-methylbutanamide in $CDCl_3$. $[\alpha]^{589}_{20}$ measured = 13.4 (*c* = 1g/100 mL).[11]

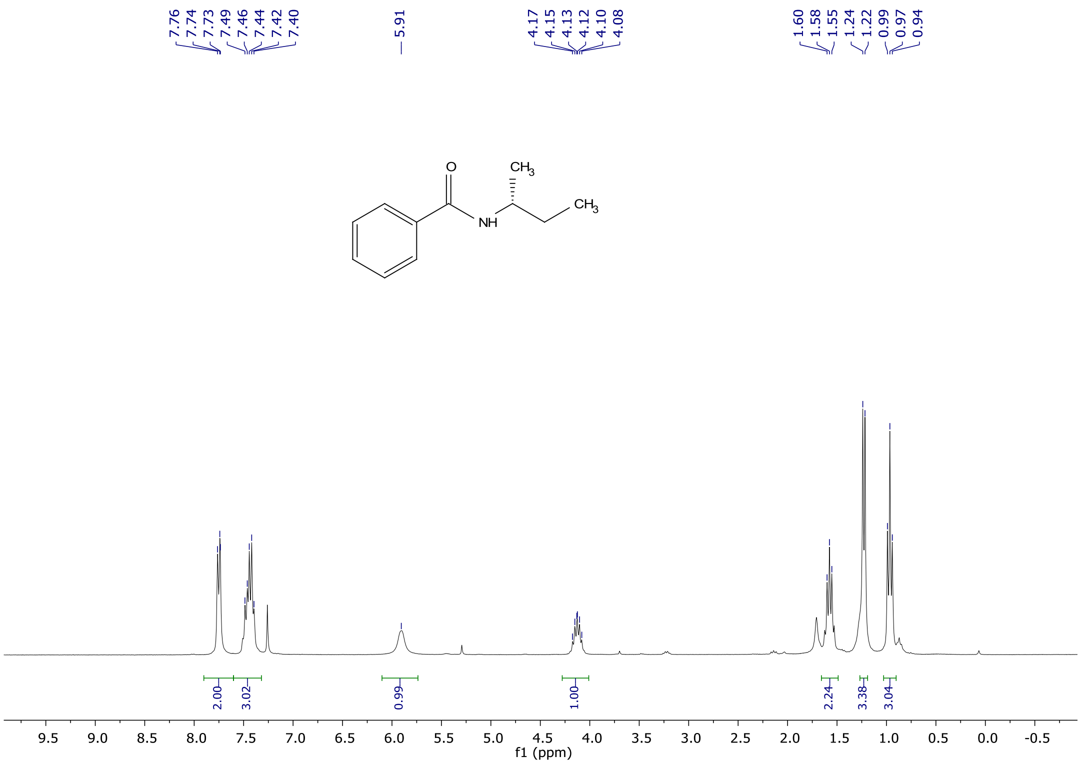

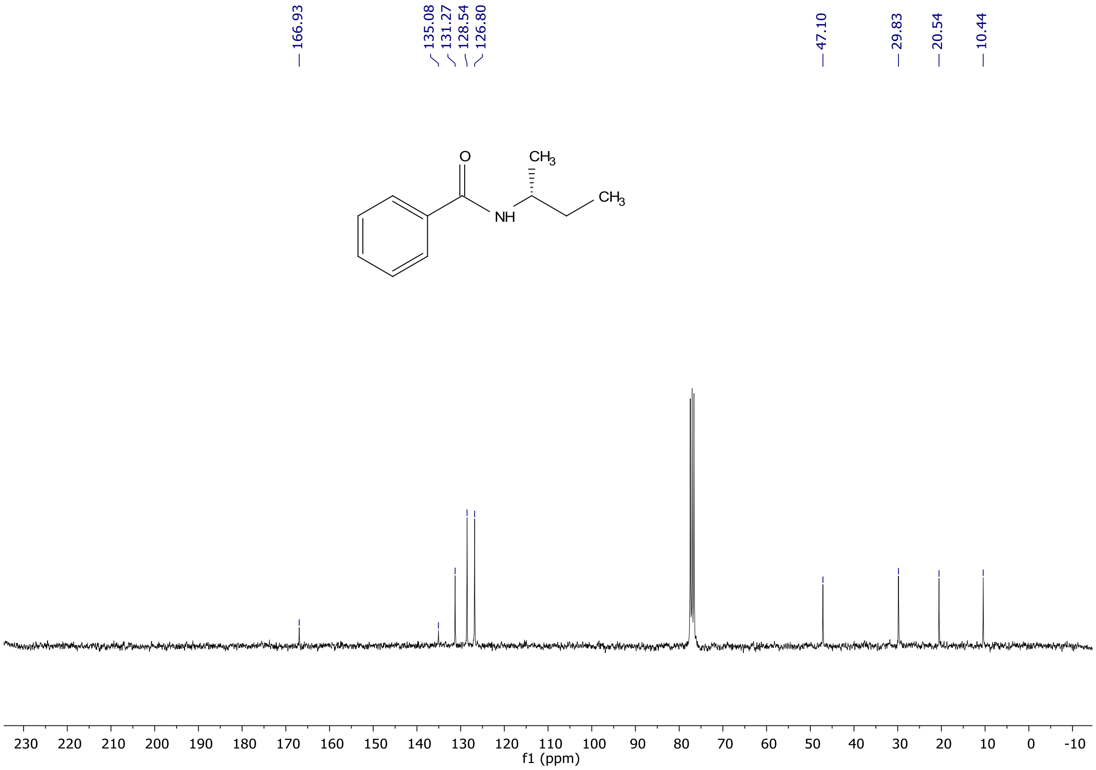

Figure S42. ^{1}H (top, 300 MHz) and $^{13}C\{^{1}H\}$ (bottom, 75 MHz) NMR spectra of isolated (*R*)-N-(sec-butyl)benzamide in $CDCl_3$. $[\alpha]^{589}_{20}$ measured = 20.0 (*c* = 1g/100 mL).[12]

9. Computational details

All geometries were optimized using Truhlar's M06-L functional,[13] the triple-ξ def2-TZVP basis set[14] and W06 density fitting to increase computational efficiency[15] as well as Grimme's D3(0) empirical dispersion correction.[16] Frequency calculations at this level of theory were run in order to confirm stationary points and transition states, as well as to compute thermodynamic properties. Single point energies of the optimized structures were computed using the range-separated meta-GGA hybrid functional ωB97M-V of the Head-Gordon group[17] including dispersion correction,[18] together with the triple-ξ def2-TZVPP basis set[14] and the corresponding auxiliary basis sets, def2/J[15] and def2-TZVPP/C, [19] for RIJCOSX density fitting. To take the influence of the solvent into account, single point calculations were performed with the integral equation formalism variant (IEFPCM) of the PCM model in the SMD variation of Truhlar and co-workers, with diethyl ether. Gibbs free energies were computed by adding the free energy correction terms from the frequency calculations to the single point energies according to

$$G^{\omega B97M\text{-}V}{}_{SMD} = E^{\omega B97M\text{-}V}{}_{el/SMD} + \text{Gcorr}^{M06\text{-}L}{}_{freq/gas} \quad (1)$$

Free energy values (G^o) were then corrected to account for changes in standard states ($G^o \rightarrow G^{o\prime}$). Specifically, all species were corrected for the condensed phase (1 atm to 1M), with the exception of H_2 (maintained at 1 atm standard state). Optimizations and frequency calculations were done using the Gaussian 16 software suite in the C.01 revision.[20] Single point calculations were performed using Gaussian 16[20] or the ORCA Software in the 4.2.1 release.[21]

Alternate concerted reaction pathway not computed in this study

Scheme S2

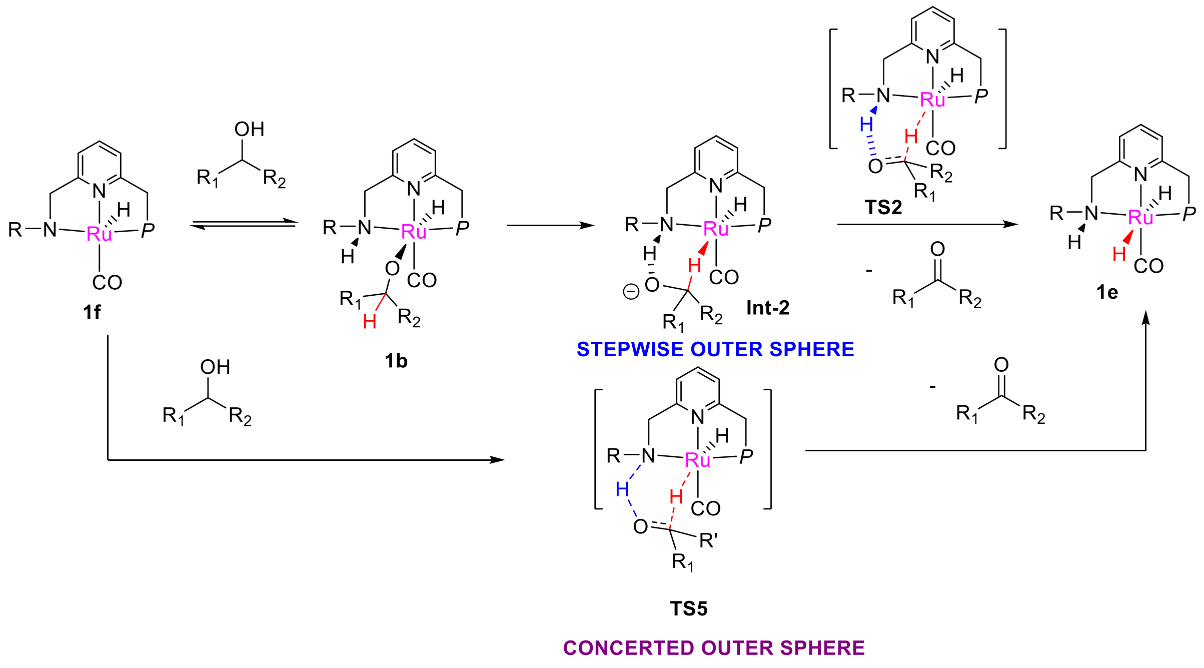

Computed Structures, First Three Frequencies, Coordinates

Note: Only relevant hydrogen atoms shown for clarity.

Color Scheme: ruthenium (pink), phosphorus (orange), nitrogen (blue), carbon (grey), hydrogen (green)

1b

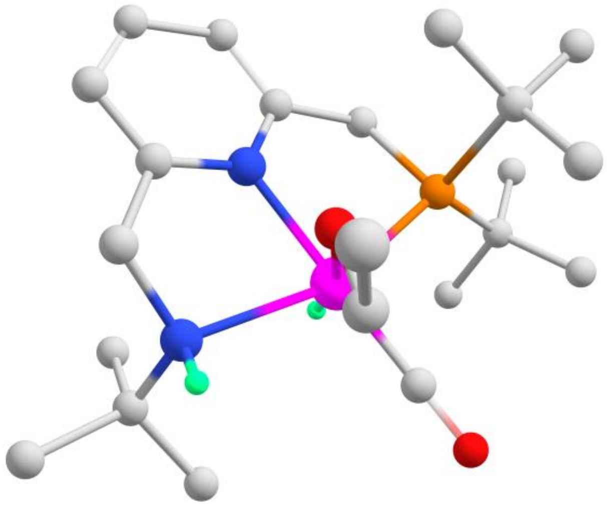

First three frequencies

	1	2	3
	A	A	A
Frequencies --	34.6601	43.2879	48.8900
Red. masses --	3.9936	3.3395	3.1858
Frc consts --	0.0028	0.0037	0.0045
IR Inten --	0.2880	0.2219	0.1714

Coordinates

44	-0.369067000	-1.156958000	-0.107984000
15	-0.467428000	1.044484000	0.211016000
7	-0.647441000	-3.403975000	0.309066000
8	0.895548000	-0.949682000	-2.810344000
6	-2.688028000	-1.324489000	4.177710000
7	-1.410226000	-1.197596000	1.755802000
6	-1.886045000	1.139709000	1.412390000
1	-1.885606000	2.045368000	2.022804000
6	-1.974151000	-0.086703000	2.260357000
6	-2.615357000	-0.124328000	3.489335000
1	-3.057055000	0.776999000	3.891970000
6	-2.117735000	-2.461123000	3.631281000
1	-2.162046000	-3.412804000	4.143902000
6	-1.470204000	-2.364326000	2.409613000
6	-0.728440000	-3.502044000	1.782766000
1	-1.148930000	-4.454306000	2.114068000
1	0.298796000	-3.435790000	2.147113000
6	0.978041000	1.794530000	1.171085000
6	2.269706000	1.430732000	0.437907000
1	2.346604000	0.350194000	0.327421000

1	3.124897000	1.773739000	1.024929000
1	2.343871000	1.890622000	-0.546046000
6	0.997986000	1.086584000	2.530321000
1	0.145166000	1.361239000	3.153767000
1	1.896351000	1.397352000	3.068896000
1	1.044671000	0.002661000	2.411288000
6	0.910749000	3.297652000	1.413235000
1	1.007593000	3.883115000	0.500534000
1	1.738692000	3.585912000	2.065393000
1	-0.009032000	3.598471000	1.917487000
6	-1.018790000	2.225865000	-1.156320000
6	-2.029741000	1.495203000	-2.040428000
1	-1.587260000	0.640918000	-2.546058000
1	-2.400381000	2.191374000	-2.796366000
1	-2.887695000	1.128161000	-1.476697000
6	-1.696538000	3.490843000	-0.626467000
1	-2.611000000	3.266648000	-0.077693000
1	-1.984076000	4.116610000	-1.474050000
1	-1.057901000	4.091690000	0.013699000
6	0.182177000	2.602240000	-2.022569000
1	0.891018000	3.242784000	-1.500969000
1	-0.166337000	3.152726000	-2.898868000
1	0.715593000	1.722738000	-2.382378000
1	0.283471000	-3.719554000	0.047758000
6	-1.638356000	-4.233440000	-0.452074000
6	-1.410148000	-5.720036000	-0.185004000
1	-0.397402000	-6.016435000	-0.462174000
1	-2.101506000	-6.318609000	-0.776873000
1	-1.568272000	-5.989152000	0.858734000
6	-1.411395000	-3.951812000	-1.928905000
1	-1.638970000	-2.913455000	-2.161630000
1	-2.043635000	-4.597409000	-2.537577000
1	-0.373427000	-4.140670000	-2.209963000
6	-3.042216000	-3.820737000	-0.043074000
1	-3.252241000	-4.063239000	0.999959000
1	-3.777472000	-4.344720000	-0.652197000
1	-3.175383000	-2.748047000	-0.186104000
6	0.427588000	-1.045913000	-1.748522000
1	-3.191975000	-1.372951000	5.134196000
1	-2.782275000	1.184767000	0.785751000
1	-1.817938000	-1.000238000	-0.828255000
8	1.368415000	-1.687432000	1.110865000
6	2.437648000	-2.291294000	0.500418000
1	2.867311000	-1.677186000	-0.318487000
1	2.169968000	-3.251138000	-0.013088000
6	3.533000000	-2.594123000	1.505849000
1	3.158363000	-3.243377000	2.299057000
1	3.871741000	-1.669232000	1.975236000
1	4.393600000	-3.080303000	1.043563000

Int 2

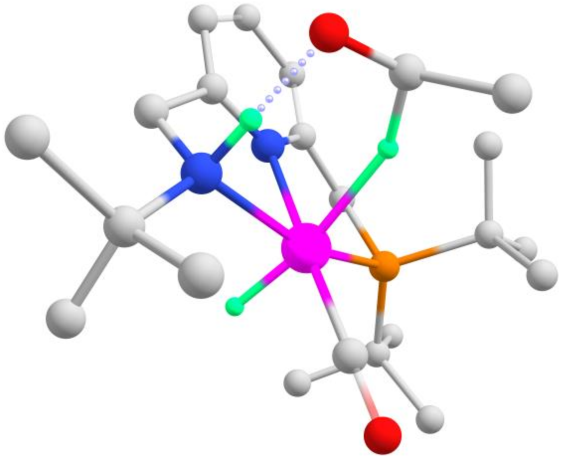

First three frequencies

```
                        1                      2                      3
                        A                      A                      A
 Frequencies --     22.2050                41.5483                45.9340
 Red. masses --      3.7004                 4.5126                 5.4340
 Frc consts  --      0.0011                 0.0046                 0.0068
```

Coordinates

44	-0.352248000	-0.842417000	0.038152000
15	-0.412739000	1.411742000	-0.104997000
7	-0.629529000	-2.900167000	0.847847000
8	1.526998000	-1.063454000	-2.281550000
6	-2.977054000	-0.181809000	4.050638000
7	-1.718012000	-0.517803000	1.640246000
6	-1.815891000	1.813343000	1.044347000
1	-1.716043000	2.788669000	1.522442000
6	-2.062724000	0.722987000	2.036173000
6	-2.684514000	0.918140000	3.257279000
1	-2.937892000	1.919795000	3.576771000
6	-2.692382000	-1.453590000	3.588045000
1	-2.934831000	-2.331161000	4.170852000
6	-2.071957000	-1.595668000	2.356467000
6	-1.827991000	-2.912892000	1.699826000
1	-2.709077000	-3.141242000	1.088577000
1	-1.760595000	-3.687754000	2.467071000
6	1.052665000	2.335755000	0.663771000
6	2.337291000	1.757564000	0.070888000
1	3.201594000	2.214384000	0.557626000
1	2.427251000	1.940726000	-0.998720000
1	2.394137000	0.681140000	0.229863000
6	1.019932000	2.021327000	2.160950000
1	0.222687000	2.558314000	2.676009000
1	1.962613000	2.331843000	2.615519000
1	0.898906000	0.957670000	2.359615000
6	1.046617000	3.851937000	0.508871000
1	1.176664000	4.177222000	-0.521194000

1	1.879815000	4.266332000	1.081149000
1	0.136124000	4.306405000	0.901469000
6	-0.935748000	2.276787000	-1.704189000
6	-1.985324000	1.396291000	-2.383990000
1	-1.570528000	0.441832000	-2.697822000
1	-2.363177000	1.915898000	-3.267037000
1	-2.837730000	1.183984000	-1.737011000
6	-1.558738000	3.656958000	-1.489269000
1	-2.457994000	3.613305000	-0.875240000
1	-1.861184000	4.059223000	-2.458363000
1	-0.880790000	4.376986000	-1.042673000
6	0.266529000	2.385813000	-2.640558000
1	-0.070804000	2.729084000	-3.620636000
1	0.759105000	1.424511000	-2.784887000
1	0.164719000	-2.930626000	1.573844000
6	-0.464657000	-4.145569000	0.020137000
6	-0.767723000	-5.389144000	0.857964000
1	-0.189958000	-5.385814000	1.783121000
1	-0.496262000	-6.282467000	0.297491000
1	-1.824874000	-5.477242000	1.107760000
6	0.997794000	-4.202302000	-0.400319000
1	1.258599000	-3.385850000	-1.068223000
1	1.197823000	-5.134923000	-0.926589000
1	1.647425000	-4.151606000	0.474785000
6	-1.372864000	-4.093164000	-1.196539000
1	-2.423572000	-4.002604000	-0.916553000
1	-1.269708000	-5.005655000	-1.784169000
1	-1.120566000	-3.243069000	-1.828695000
6	0.813070000	-1.011689000	-1.364872000
1	-3.442299000	-0.045070000	5.017959000
1	-1.550164000	-0.949986000	-1.001338000
1	-2.705808000	1.875545000	0.409929000
8	1.127577000	-2.755054000	2.721525000
6	1.546429000	-1.527136000	2.520340000
1	1.034467000	-1.018207000	1.563833000
6	3.035735000	-1.368876000	2.237834000
1	3.604907000	-1.786728000	3.067916000
1	3.325542000	-0.323019000	2.115881000
1	1.236696000	-0.799157000	3.310125000
1	3.312710000	-1.915424000	1.335466000
1	1.008953000	3.100040000	-2.289185000

Int 3

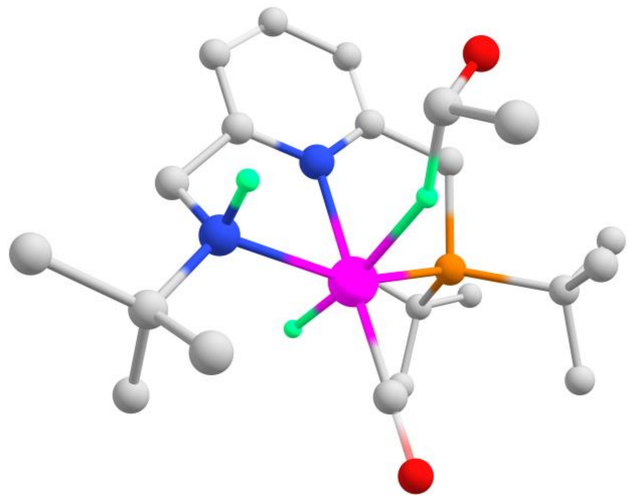

First three frequencies

	1	2	3
	A	A	A
Frequencies --	39.0330	49.6880	61.3649
Red. masses --	4.2263	4.7554	4.3068
Frc consts --	0.0038	0.0069	0.0096
IR Inten --	0.7168	1.6185	2.1183

Coordinates

44	-0.101565000	-0.632260000	-0.608338000
1	-0.641421000	-1.900634000	-1.454640000
15	-2.179828000	-0.042038000	0.030359000
8	-0.190702000	0.900898000	-3.177959000
7	1.962109000	-1.606144000	-0.440290000
7	-0.172423000	-1.745915000	1.209561000
6	-2.045512000	-0.369432000	1.842510000
6	-1.094537000	-1.474205000	2.148084000
6	-1.104778000	-2.188568000	3.338952000
1	-1.852551000	-1.966533000	4.087270000
6	-0.134295000	-3.145924000	3.563055000
1	-0.119024000	-3.696550000	4.494493000
6	0.830520000	-3.387106000	2.596199000
1	1.610729000	-4.119686000	2.750610000
6	0.776941000	-2.668625000	1.416419000
6	1.720770000	-2.874644000	0.274463000
1	2.644655000	-3.329875000	0.641066000
1	1.272675000	-3.573556000	-0.437886000
6	-0.133272000	0.316233000	-2.173317000
6	-2.713789000	1.764253000	-0.009248000
6	-1.456278000	2.591364000	0.265241000
1	-1.732219000	3.647526000	0.304246000
1	-0.990104000	2.333746000	1.219687000
1	-0.710458000	2.467713000	-0.518599000
6	-3.251675000	2.128919000	-1.390150000

1	-3.385922000	3.210954000	-1.446568000
1	-2.563218000	1.846017000	-2.186303000
1	-4.219774000	1.674529000	-1.596687000
6	-3.742524000	2.118187000	1.065582000
1	-4.684628000	1.587854000	0.960748000
1	-3.359010000	1.942030000	2.069588000
1	-3.964211000	3.185150000	0.995975000
6	-3.579498000	-1.161547000	-0.566451000
6	-3.564862000	-1.216990000	-2.093331000
1	-3.795931000	-0.257101000	-2.550997000
1	-2.593555000	-1.542148000	-2.463286000
1	-4.315742000	-1.932020000	-2.436055000
6	-4.964332000	-0.754920000	-0.074863000
1	-5.684925000	-1.524483000	-0.360960000
1	-5.009956000	-0.662134000	1.010974000
1	-5.306982000	0.180710000	-0.512774000
6	-3.275703000	-2.563000000	-0.030912000
1	-2.274095000	-2.890685000	-0.309002000
1	-3.370997000	-2.620256000	1.054223000
1	-3.990971000	-3.269921000	-0.455737000
6	2.909060000	-1.696492000	-1.605393000
6	3.293670000	-0.265838000	-1.949859000
1	2.418054000	0.334420000	-2.188426000
1	3.813860000	0.210798000	-1.116767000
1	3.961320000	-0.251069000	-2.809674000
1	-3.006603000	-0.507107000	2.340938000
6	1.234170000	0.966834000	1.636401000
1	0.627613000	0.679487000	0.593374000
8	0.465386000	1.003244000	2.658906000
6	1.938944000	2.255803000	1.216087000
1	2.647720000	2.544458000	1.992675000
1	1.222225000	3.068317000	1.106625000
1	2.004175000	0.130909000	1.633688000
1	-1.566727000	0.529190000	2.259900000
1	2.479240000	2.136166000	0.273912000
1	2.389219000	-0.968610000	0.237891000
6	4.168969000	-2.474069000	-1.229087000
1	3.974438000	-3.535885000	-1.083343000
1	4.904890000	-2.390120000	-2.027188000
1	4.624314000	-2.079470000	-0.319163000
6	2.210841000	-2.361695000	-2.778681000
1	2.895296000	-2.430378000	-3.623552000
1	1.887310000	-3.374667000	-2.538763000
1	1.334759000	-1.794425000	-3.085639000

TS1

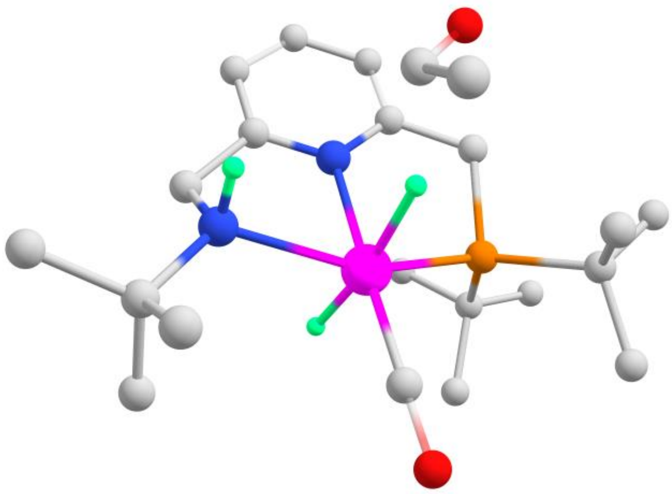

First three frequencies

		1	2	3
		1	2	3
		A	A	A
Frequencies	--	-428.8623	37.3502	49.4442
Red. masses	--	2.1631	4.2658	4.7410
Frc consts	--	0.2344	0.0035	0.0068
IR Inten	--	1111.6610	0.3269	1.2909

Coordinates

44	-0.101565000	-0.632260000	-0.608338000
1	-0.641421000	-1.900634000	-1.454640000
15	-2.179828000	-0.042038000	0.030359000
8	-0.190702000	0.900898000	-3.177959000
7	1.962109000	-1.606144000	-0.440290000
7	-0.172423000	-1.745915000	1.209561000
6	-2.045512000	-0.369432000	1.842510000
6	-1.094537000	-1.474205000	2.148084000
6	-1.104778000	-2.188568000	3.338952000
1	-1.852551000	-1.966533000	4.087270000
6	-0.134295000	-3.145924000	3.563055000
1	-0.119024000	-3.696550000	4.494493000
6	0.830520000	-3.387106000	2.596199000
1	1.610729000	-4.119686000	2.750610000
6	0.776941000	-2.668625000	1.416419000
6	1.720770000	-2.874644000	0.274463000
1	2.644655000	-3.329875000	0.641066000
1	1.272675000	-3.573556000	-0.437886000
6	-0.133272000	0.316233000	-2.173317000
6	-2.713789000	1.764253000	-0.009248000
6	-1.456278000	2.591364000	0.265241000
1	-1.732219000	3.647526000	0.304246000
1	-0.990104000	2.333746000	1.219687000
1	-0.710458000	2.467713000	-0.518599000

6	-3.251675000	2.128919000	-1.390150000
1	-3.385922000	3.210954000	-1.446568000
1	-2.563218000	1.846017000	-2.186303000
1	-4.219774000	1.674529000	-1.596687000
6	-3.742524000	2.118187000	1.065582000
1	-4.684628000	1.587854000	0.960748000
1	-3.359010000	1.942030000	2.069588000
1	-3.964211000	3.185150000	0.995975000
6	-3.579498000	-1.161547000	-0.566451000
6	-3.564862000	-1.216990000	-2.093331000
1	-3.795931000	-0.257101000	-2.550997000
1	-2.593555000	-1.542148000	-2.463286000
1	-4.315742000	-1.932020000	-2.436055000
6	-4.964332000	-0.754920000	-0.074863000
1	-5.684925000	-1.524483000	-0.360960000
1	-5.009956000	-0.662134000	1.010974000
1	-5.306982000	0.180710000	-0.512774000
6	-3.275703000	-2.563000000	-0.030912000
1	-2.274095000	-2.890685000	-0.309002000
1	-3.370997000	-2.620256000	1.054223000
1	-3.990971000	-3.269921000	-0.455737000
6	2.909060000	-1.696492000	-1.605393000
6	3.293670000	-0.265838000	-1.949859000
1	2.418054000	0.334420000	-2.188426000
1	3.813860000	0.210798000	-1.116767000
1	3.961320000	-0.251069000	-2.809674000
1	-3.006603000	-0.507107000	2.340938000
6	1.234170000	0.966834000	1.636401000
1	0.627613000	0.679487000	0.593374000
8	0.465386000	1.003244000	2.658906000
6	1.938944000	2.255803000	1.216087000
1	2.647720000	2.544458000	1.992675000
1	1.222225000	3.068317000	1.106625000
1	2.004175000	0.130909000	1.633688000
1	-1.566727000	0.529190000	2.259900000
1	2.479240000	2.136166000	0.273912000
1	2.389219000	-0.968610000	0.237891000
6	4.168969000	-2.474069000	-1.229087000
1	3.974438000	-3.535885000	-1.083343000
1	4.904890000	-2.390120000	-2.027188000
1	4.624314000	-2.079470000	-0.319163000
6	2.210841000	-2.361695000	-2.778681000
1	2.895296000	-2.430378000	-3.623552000
1	1.887310000	-3.374667000	-2.538763000
1	1.334759000	-1.794425000	-3.085639000

TS2

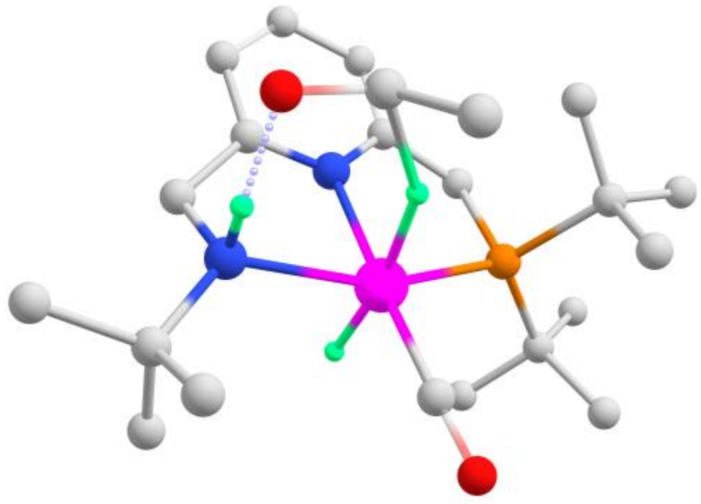

First three frequencies

```
                        1                      2                      3
                        A                      A                      A
 Frequencies --    -436.0669               22.1130                33.4792
 Red. masses --       2.2481                3.7135                 3.4662
 Frc consts  --       0.2519                0.0011                 0.0023
 IR Inten    --    1106.6903                0.1362                 0.0671
```

Coordinates

44	-0.311397000	-0.831691000	0.071887000
15	-0.380718000	1.404858000	-0.078337000
7	-0.612353000	-2.925553000	0.888843000
8	1.587901000	-1.059382000	-2.228727000
6	-3.027657000	-0.186809000	4.041585000
7	-1.695695000	-0.519429000	1.666318000
6	-1.782237000	1.811714000	1.072972000
1	-1.677317000	2.783744000	1.557022000
6	-2.049465000	0.718929000	2.055974000
6	-2.706475000	0.912434000	3.260841000
1	-2.966600000	1.914338000	3.574483000
6	-2.734622000	-1.458548000	3.579814000
1	-3.003326000	-2.338163000	4.148388000
6	-2.077823000	-1.595633000	2.367627000
6	-1.830247000	-2.916615000	1.713324000
1	-2.687785000	-3.123385000	1.063754000
1	-1.808765000	-3.700039000	2.475149000
6	1.080103000	2.358626000	0.674949000
6	2.369855000	1.803708000	0.071895000
1	3.230184000	2.259676000	0.567344000
1	2.458431000	2.009723000	-0.993541000
1	2.429539000	0.724743000	0.208363000

6	1.070782000	2.049884000	2.173299000
1	0.265523000	2.567686000	2.695832000
1	2.009992000	2.386050000	2.617520000
1	0.975188000	0.981853000	2.361051000
6	1.047051000	3.874428000	0.517469000
1	1.165435000	4.199506000	-0.514061000
1	1.876554000	4.305040000	1.083477000
1	0.131198000	4.314462000	0.914370000
6	-0.915657000	2.268487000	-1.679020000
6	-1.964906000	1.384251000	-2.353397000
1	-1.553990000	0.423089000	-2.649921000
1	-2.337341000	1.897787000	-3.242668000
1	-2.818865000	1.180016000	-1.706128000
6	-1.543733000	3.647632000	-1.470372000
1	-2.438268000	3.604696000	-0.849331000
1	-1.856638000	4.039250000	-2.440674000
1	-0.867669000	4.376609000	-1.036140000
6	0.282273000	2.377709000	-2.620949000
1	-0.060830000	2.710680000	-3.602725000
1	0.779679000	1.417842000	-2.759042000
1	0.159807000	-2.950322000	1.569362000
6	-0.445729000	-4.168149000	0.054242000
6	-0.766948000	-5.415274000	0.878526000
1	-0.201089000	-5.426133000	1.811542000
1	-0.495982000	-6.307072000	0.315392000
1	-1.827425000	-5.496292000	1.115810000
6	1.020993000	-4.221693000	-0.349132000
1	1.295347000	-3.379642000	-0.978741000
1	1.221083000	-5.135490000	-0.907051000
1	1.664103000	-4.213091000	0.532531000
6	-1.337247000	-4.096905000	-1.172042000
1	-2.391688000	-4.013709000	-0.905698000
1	-1.221678000	-5.001795000	-1.768535000
1	-1.081270000	-3.235023000	-1.785409000
6	0.856651000	-0.998644000	-1.323557000
1	-3.522513000	-0.052346000	4.994382000
1	-1.591973000	-1.017867000	-0.948206000
1	-2.669341000	1.884485000	0.435627000
8	1.243251000	-2.735662000	2.887560000
6	1.677435000	-1.583050000	2.734432000
1	0.956936000	-0.866238000	1.349866000
6	3.088141000	-1.334355000	2.276088000
1	3.766897000	-1.641615000	3.075619000
1	3.269970000	-0.283630000	2.058946000
1	1.233368000	-0.759592000	3.332176000
1	3.320651000	-1.931371000	1.396249000
1	1.021928000	3.099448000	-2.278723000

1e

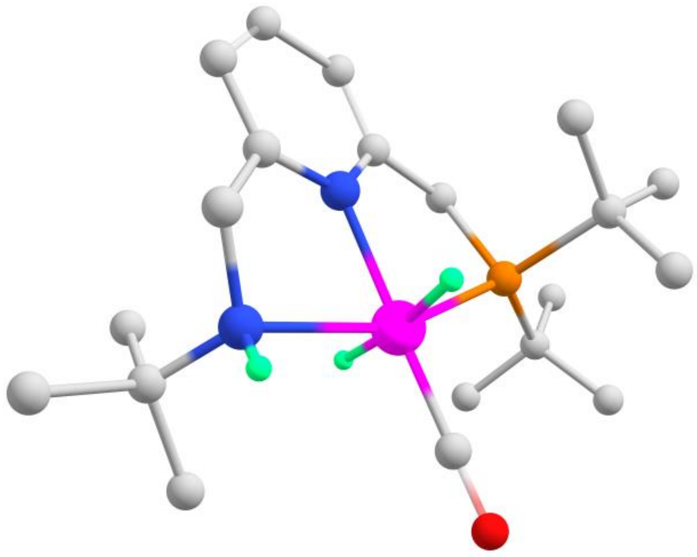

First three frequencies

	1	2	3
	A	A	A
Frequencies --	***45.5135***	48.7827	66.4632
Red. masses --	***5.3563***	3.6089	5.6567
Frc consts --	***0.0065***	0.0051	0.0147
IR Inten --	***1.2990***	0.2361	1.0997

Coordinates

44	-0.358366000	-1.142921000	-0.176958000
15	-0.453208000	1.046383000	0.193181000
7	-0.609723000	-3.399756000	0.202864000
8	1.046092000	-0.935724000	-2.815067000
6	-2.654112000	-1.430749000	4.108097000
7	-1.364407000	-1.224884000	1.692396000
6	-1.871541000	1.109413000	1.400949000
1	-1.887316000	2.003180000	2.028721000
6	-1.948884000	-0.134831000	2.222220000
6	-2.594932000	-0.214344000	3.446577000
1	-3.054061000	0.670099000	3.867298000
6	-2.055867000	-2.543981000	3.540262000
1	-2.082432000	-3.507680000	4.031563000
6	-1.402221000	-2.404335000	2.328185000
6	-0.605030000	-3.489047000	1.676947000
1	-0.919881000	-4.469599000	2.044695000
1	0.435139000	-3.337174000	1.981979000
6	0.984980000	1.804655000	1.175951000
6	2.290088000	1.507307000	0.438784000

1	2.390056000	0.437714000	0.255853000
1	3.133490000	1.831424000	1.052583000
1	2.363318000	2.025337000	-0.515535000
6	1.026094000	1.066321000	2.516890000
1	0.181821000	1.324106000	3.158168000
1	1.933649000	1.352336000	3.052260000
1	1.048645000	-0.014421000	2.371821000
6	0.874415000	3.296998000	1.465513000
1	0.960796000	3.910603000	0.570827000
1	1.688814000	3.591645000	2.131859000
1	-0.057650000	3.556178000	1.970191000
6	-1.010798000	2.268437000	-1.140446000
6	-2.005782000	1.551107000	-2.052968000
1	-1.553522000	0.704608000	-2.562934000
1	-2.365999000	2.260973000	-2.801617000
1	-2.868794000	1.168515000	-1.508480000
6	-1.709221000	3.510833000	-0.585066000
1	-2.622019000	3.259708000	-0.045228000
1	-2.004542000	4.150268000	-1.419885000
1	-1.084008000	4.109134000	0.070438000
6	0.191564000	2.680265000	-1.988563000
1	0.885690000	3.323215000	-1.450102000
1	-0.157738000	3.240348000	-2.858512000
1	0.742159000	1.815409000	-2.358851000
1	0.296043000	-3.723622000	-0.118310000
6	-1.652239000	-4.224520000	-0.500182000
6	-1.410550000	-5.711388000	-0.249355000
1	-0.415950000	-6.010692000	-0.583916000
1	-2.134907000	-6.307112000	-0.803391000
1	-1.512296000	-5.983551000	0.800841000
6	-1.510910000	-3.933690000	-1.985248000
1	-1.716655000	-2.883845000	-2.186564000
1	-2.203586000	-4.550440000	-2.556915000
1	-0.500917000	-4.155219000	-2.335447000
6	-3.028434000	-3.811948000	-0.007910000
1	-3.189365000	-4.087481000	1.035521000
1	-3.797288000	-4.310326000	-0.596580000
1	-3.155654000	-2.734089000	-0.116824000
6	0.504487000	-1.029219000	-1.788077000
1	-3.164132000	-1.509664000	5.059062000
1	1.122975000	-1.448774000	0.643580000
1	-2.763929000	1.151431000	0.768020000
1	-1.899682000	-1.016933000	-0.871167000

1g

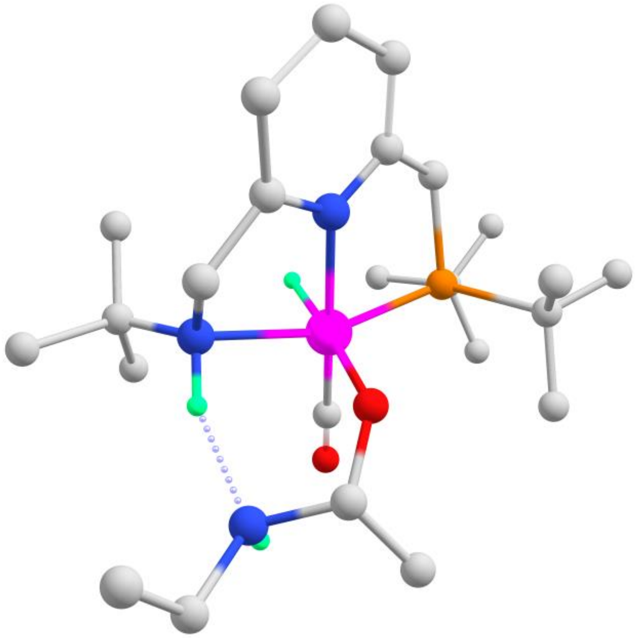

First three frequencies

	1	2	3
	A	A	A
Frequencies --	31.9365	44.1770	53.1675
Red. masses --	3.9347	3.5347	3.9008
Frc consts --	0.0024	0.0041	0.0065
IR Inten --	0.0538	0.3910	0.1451

Coordinates

44	-0.175027000	-0.874080000	-0.227645000
15	-0.506565000	1.267076000	0.334734000
7	-0.342273000	-3.150690000	-0.102874000
8	1.053252000	-0.283531000	-2.887317000
6	-2.473974000	-1.744756000	3.994638000
7	-1.209905000	-1.221242000	1.618785000
6	-1.918803000	1.077922000	1.530663000
1	-2.010403000	1.909288000	2.232710000
6	-1.875663000	-0.234249000	2.241160000
6	-2.510319000	-0.470749000	3.451124000
1	-3.033558000	0.333929000	3.949495000
6	-1.814334000	-2.756527000	3.319369000
1	-1.782886000	-3.764738000	3.710440000
6	-1.180636000	-2.461863000	2.121935000
6	-0.383845000	-3.453325000	1.338008000
1	-0.736435000	-4.466900000	1.546878000
1	0.648655000	-3.378934000	1.691244000
6	0.829541000	2.088107000	1.389464000

6	2.143700000	2.029206000	0.615794000
1	2.332428000	1.011127000	0.282552000
1	2.965952000	2.326914000	1.270657000
1	2.159390000	2.689799000	-0.248695000
6	0.979925000	1.197087000	2.627693000
1	0.105857000	1.241830000	3.279711000
1	1.829305000	1.556993000	3.212887000
1	1.183626000	0.160267000	2.350405000
6	0.546524000	3.514643000	1.842939000
1	0.550423000	4.228933000	1.021010000
1	1.329972000	3.825895000	2.538216000
1	-0.402586000	3.607668000	2.373348000
6	-1.203055000	2.515644000	-0.902359000
6	-2.110445000	1.769862000	-1.880773000
1	-1.562211000	1.041012000	-2.472210000
1	-2.563513000	2.495599000	-2.560164000
1	-2.917060000	1.237319000	-1.376472000
6	-2.038523000	3.616571000	-0.246090000
1	-2.928300000	3.219721000	0.241978000
1	-2.385254000	4.303769000	-1.020854000
1	-1.489189000	4.205005000	0.482557000
6	-0.058365000	3.139742000	-1.698510000
1	0.541710000	3.822457000	-1.099377000
1	-0.470765000	3.716519000	-2.528900000
1	0.602503000	2.383960000	-2.123156000
1	0.630602000	-3.339862000	-0.404403000
6	-1.286649000	-3.928682000	-0.958227000
6	-0.936369000	-5.416643000	-0.917223000
1	0.078795000	-5.582763000	-1.281388000
1	-1.614241000	-5.983589000	-1.554786000
1	-1.009831000	-5.839755000	0.084397000
6	-1.117132000	-3.432085000	-2.386708000
1	-1.429012000	-2.393528000	-2.475782000
1	-1.711339000	-4.038358000	-3.070190000
1	-0.073789000	-3.499267000	-2.700548000
6	-2.712799000	-3.697332000	-0.485807000
1	-2.883008000	-4.102152000	0.513397000
1	-3.418215000	-4.185476000	-1.157560000
1	-2.934794000	-2.629743000	-0.469479000
6	0.592186000	-0.521513000	-1.842503000
1	-2.968996000	-1.948569000	4.935290000
1	-2.819899000	1.093215000	0.909532000
1	-1.636914000	-0.750208000	-0.896262000
8	1.652234000	-1.426512000	0.924315000
6	2.760783000	-2.026481000	0.431856000
1	3.251358000	-2.612570000	1.236381000
6	3.802046000	-1.058750000	-0.121028000
1	4.119001000	-0.353167000	0.646132000
1	3.378015000	-0.489314000	-0.951872000
1	4.690476000	-1.580139000	-0.485282000
7	2.458472000	-3.059792000	-0.629616000

1	2.492089000	-2.586185000	-1.527625000
6	3.376707000	-4.193101000	-0.655709000
1	3.299867000	-4.668884000	-1.636653000
1	4.427402000	-3.879513000	-0.554839000
6	3.044362000	-5.203134000	0.422885000
1	2.028071000	-5.580706000	0.303014000
1	3.114207000	-4.759792000	1.416106000
1	3.725800000	-6.052604000	0.394814000

TS3

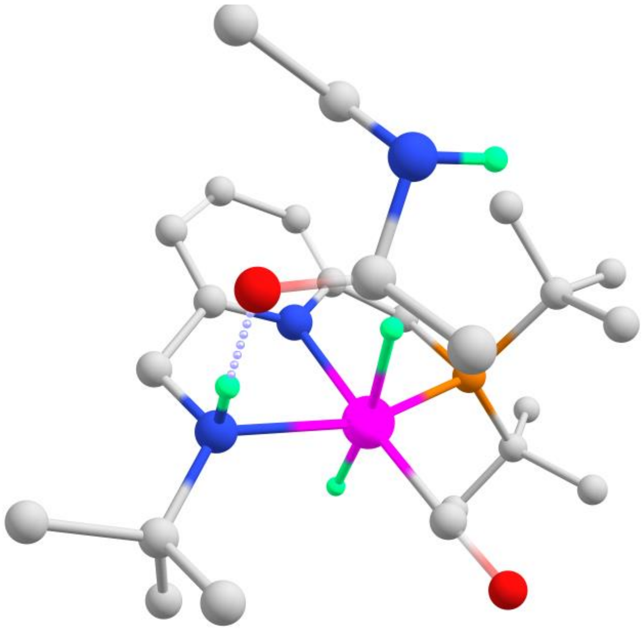

First three frequencies

	1	2	3
	A	A	A
Frequencies --	-628.9627	24.2961	42.5423
Red. masses --	1.9114	3.7489	3.5225
Frc consts --	0.4455	0.0013	0.0038
IR Inten --	2357.4464	0.3683	0.4843

Coordinates

44	-0.308031000	-0.912469000	0.142530000
15	-0.407754000	1.332740000	0.049372000
7	-0.680631000	-3.026629000	0.828318000
8	1.748896000	-1.008195000	-2.028654000
6	-3.310539000	-0.366010000	3.919357000
7	-1.809243000	-0.658881000	1.641573000
6	-1.959259000	1.659036000	1.014815000
1	-1.982196000	2.644108000	1.483147000
6	-2.250550000	0.565591000	1.989588000

6	-2.991778000	0.740400000	3.146021000
1	-3.317250000	1.732021000	3.429706000
6	-2.935692000	-1.626602000	3.490424000
1	-3.201561000	-2.512969000	4.050071000
6	-2.194190000	-1.745449000	2.324737000
6	-1.855774000	-3.062350000	1.709790000
1	-2.731502000	-3.367656000	1.126020000
1	-1.734648000	-3.810102000	2.499035000
6	0.895102000	2.293376000	1.040475000
6	2.273474000	1.785073000	0.624477000
1	3.040255000	2.241134000	1.255098000
1	2.517754000	2.017640000	-0.410757000
1	2.331844000	0.703019000	0.741857000
6	0.661839000	1.900677000	2.499176000
1	-0.214162000	2.389128000	2.927414000
1	1.522174000	2.199771000	3.102756000
1	0.538635000	0.822980000	2.601019000
6	0.842647000	3.812864000	0.947583000
1	1.099116000	4.191335000	-0.039684000
1	1.568109000	4.234008000	1.647791000
1	-0.133485000	4.212265000	1.225780000
6	-0.733789000	2.234025000	-1.581038000
6	-1.622246000	1.338705000	-2.445021000
1	-1.123668000	0.410885000	-2.713620000
1	-1.875551000	1.875510000	-3.361910000
1	-2.555516000	1.071734000	-1.947969000
6	-1.453083000	3.572996000	-1.410077000
1	-2.434982000	3.457061000	-0.951481000
1	-1.618071000	4.010803000	-2.396777000
1	-0.893357000	4.298715000	-0.828639000
6	0.587477000	2.440957000	-2.320519000
1	0.380935000	2.812765000	-3.326152000
1	1.145019000	1.510813000	-2.426849000
1	0.142504000	-3.108665000	1.481372000
6	-0.596616000	-4.226715000	-0.078176000
6	-0.885056000	-5.507713000	0.707536000
1	-0.246555000	-5.575002000	1.589413000
1	-0.681938000	-6.375521000	0.081746000
1	-1.924817000	-5.579453000	1.025549000
6	0.832318000	-4.301284000	-0.594346000
1	1.082105000	-3.443985000	-1.213978000
1	0.959692000	-5.196385000	-1.202073000
1	1.537614000	-4.346192000	0.236066000
6	-1.570071000	-4.079521000	-1.234921000
1	-2.603523000	-3.994125000	-0.895763000
1	-1.513722000	-4.953042000	-1.884475000
1	-1.336826000	-3.191751000	-1.820720000
6	0.956316000	-1.006633000	-1.176010000
1	-3.869749000	-0.245406000	4.837806000
1	-1.497223000	-0.977855000	-0.948220000
1	-2.765569000	1.650032000	0.274099000

8	1.298750000	-3.025644000	2.559554000
6	1.884958000	-1.906928000	2.332976000
1	1.048863000	-1.156938000	1.547144000
6	3.135961000	-1.941491000	1.466466000
1	3.886386000	-2.550123000	1.970936000
1	3.559819000	-0.953021000	1.286490000
1	2.907716000	-2.396575000	0.505766000
1	1.231455000	3.172051000	-1.835189000
7	2.087012000	-1.070632000	3.484065000
1	2.463257000	-0.170926000	3.217711000
6	0.949333000	-0.940596000	4.379248000
1	1.022406000	0.031317000	4.873864000
1	0.013262000	-0.918548000	3.796686000
6	0.871208000	-2.041038000	5.415226000
1	1.765401000	-2.052556000	6.037323000
1	0.787878000	-3.009020000	4.927576000
1	0.006827000	-1.895620000	6.065438000

TS4

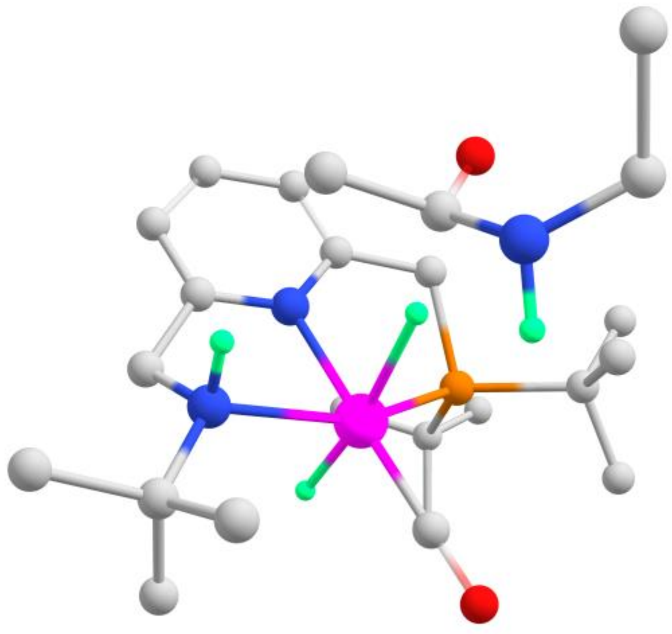

First three frequencies

	1	2	3
	A	A	A
Frequencies --	***-484.7245***	29.0918	42.4845
Red. masses --	***2.0209***	4.1131	4.4006
Frc consts --	***0.2798***	0.0021	0.0047
IR Inten --	***2334.5875***	1.1867	2.7602

Coordinates

44	-0.070946000	-0.748359000	-0.760916000
1	-0.715360000	-2.022039000	-1.551688000
15	-2.089966000	-0.066292000	-0.043402000
8	-0.201717000	0.762032000	-3.342984000
7	1.902314000	-1.924212000	-0.710200000
7	-0.119763000	-1.827217000	1.073716000
6	-1.831864000	-0.288762000	1.775326000
6	-0.951905000	-1.456385000	2.062152000
6	-0.936428000	-2.139325000	3.270551000
1	-1.599602000	-1.830432000	4.066482000
6	-0.058879000	-3.194874000	3.443372000
1	-0.030117000	-3.728495000	4.384256000
6	0.789551000	-3.561193000	2.409253000
1	1.488418000	-4.379151000	2.518533000
6	0.730705000	-2.851890000	1.223648000
6	1.563528000	-3.160939000	0.019364000
1	2.448901000	-3.727460000	0.321060000
1	0.982194000	-3.793532000	-0.658339000
6	-0.128086000	0.180571000	-2.336710000
6	-2.623583000	1.734389000	-0.177707000
6	-1.342730000	2.562761000	-0.100699000
1	-1.605626000	3.623247000	-0.097132000
1	-0.779892000	2.354220000	0.810299000
1	-0.690394000	2.382043000	-0.953429000
6	-3.296379000	1.997764000	-1.522060000
1	-3.421400000	3.074896000	-1.649390000
1	-2.692072000	1.641748000	-2.356677000
1	-4.285518000	1.548760000	-1.597720000
6	-3.530931000	2.183225000	0.968570000
1	-4.467482000	1.635811000	1.027647000
1	-3.028528000	2.109855000	1.932330000
1	-3.780928000	3.236171000	0.823045000
6	-3.546469000	-1.200597000	-0.465574000
6	-3.661815000	-1.339719000	-1.982981000
1	-3.943173000	-0.409888000	-2.472115000
1	-2.722082000	-1.680428000	-2.415783000
1	-4.431057000	-2.078116000	-2.219088000
6	-4.879653000	-0.744775000	0.118766000
1	-5.633008000	-1.513208000	-0.069255000
1	-4.832414000	-0.601646000	1.199129000
1	-5.244889000	0.175847000	-0.331656000
6	-3.226815000	-2.580960000	0.113315000
1	-2.263664000	-2.951148000	-0.237556000
1	-3.227434000	-2.583010000	1.204042000
1	-3.994821000	-3.286566000	-0.209558000
6	2.754209000	-2.109250000	-1.935931000
6	3.224122000	-0.718936000	-2.337993000
1	2.382823000	-0.062914000	-2.550688000
1	3.819095000	-0.257572000	-1.547506000

1	3.846155000	-0.775530000	-3.229748000
1	-2.752312000	-0.324654000	2.361002000
6	1.438206000	1.193505000	1.374617000
1	0.803938000	0.535219000	0.350806000
8	0.644420000	1.409438000	2.332376000
6	2.593282000	0.237237000	1.670660000
1	2.232080000	-0.724399000	2.040464000
1	3.195123000	0.689955000	2.459540000
1	-1.263439000	0.601882000	2.087575000
1	3.254523000	0.101061000	0.811364000
1	2.449269000	-1.349104000	-0.073755000
6	3.972415000	-2.976153000	-1.622457000
1	3.705845000	-4.015248000	-1.433164000
1	4.657615000	-2.971041000	-2.468669000
1	4.517720000	-2.597229000	-0.755906000
6	1.929943000	-2.734973000	-3.046647000
1	2.546244000	-2.855212000	-3.936871000
1	1.557642000	-3.722393000	-2.773477000
1	1.074132000	-2.111222000	-3.296063000
7	1.956079000	2.360144000	0.626269000
1	1.720641000	2.304363000	-0.352663000
6	1.649695000	3.666022000	1.187156000
1	1.846963000	4.401689000	0.404072000
1	0.594107000	3.772164000	1.463358000
6	2.502100000	3.957253000	2.403780000
1	3.563283000	3.895958000	2.161593000
1	2.289309000	3.234285000	3.190457000
1	2.297338000	4.951772000	2.800670000

2b (Bn alkoxy complex)

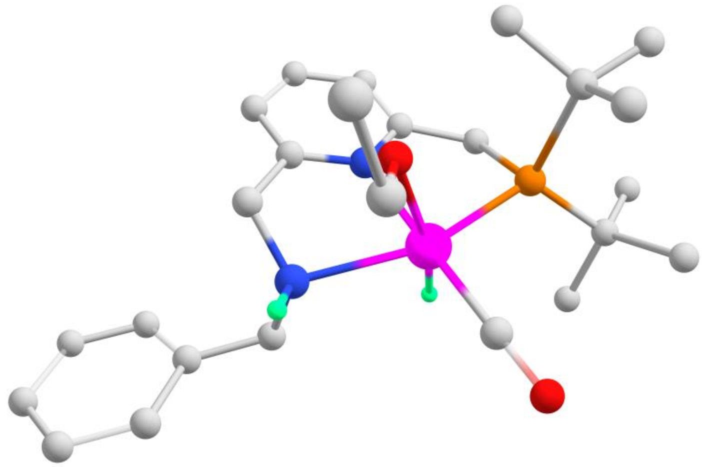

First three frequencies

	1	2	3
	A	A	A
Frequencies --	***19.5928***	27.6589	35.6768
Red. masses --	***5.1137***	4.5670	3.7471
Frc consts --	***0.0012***	0.0021	0.0028
IR Inten --	***0.3535***	0.1547	0.0660

Coordinates

44	-0.122338000	-1.214339000	-0.114591000
15	-0.433737000	0.969972000	0.195729000
7	-0.305611000	-3.451750000	0.274030000
8	1.199589000	-0.881250000	-2.776630000
6	-2.556934000	-1.609626000	4.092010000
7	-1.216671000	-1.354292000	1.715431000
6	-1.887998000	0.931665000	1.358812000
1	-1.984905000	1.831873000	1.969514000
6	-1.891375000	-0.300390000	2.203504000
6	-2.564879000	-0.402676000	3.412331000
1	-3.095043000	0.454221000	3.805188000
6	-1.874056000	-2.690950000	3.559887000
1	-1.853189000	-3.647131000	4.065604000
6	-1.195036000	-2.527791000	2.363846000
6	-0.332341000	-3.580833000	1.744696000
1	-0.637143000	-4.580260000	2.068139000
1	0.688830000	-3.388562000	2.081833000
6	0.908818000	1.852053000	1.193150000
6	2.248184000	1.597283000	0.500025000
1	2.421880000	0.526077000	0.407575000
1	3.052626000	2.021966000	1.105131000
1	2.308140000	2.051317000	-0.487733000
6	0.949362000	1.158964000	2.559403000
1	0.056462000	1.361446000	3.153821000
1	1.799318000	1.552706000	3.121806000
1	1.096680000	0.083005000	2.453179000
6	0.702746000	3.345206000	1.418945000
1	0.770017000	3.930543000	0.503525000
1	1.485501000	3.710124000	2.088333000
1	-0.252204000	3.567213000	1.898229000
6	-1.055968000	2.094223000	-1.188190000
6	-1.971945000	1.271592000	-2.094584000
1	-1.440062000	0.458974000	-2.582179000
1	-2.383895000	1.928490000	-2.864174000
1	-2.807934000	0.829938000	-1.551681000
6	-1.860359000	3.292646000	-0.681252000
1	-2.764196000	2.986738000	-0.154630000
1	-2.182686000	3.887351000	-1.538658000
1	-1.296238000	3.951266000	-0.028116000
6	0.127923000	2.577302000	-2.024625000
1	0.762059000	3.279711000	-1.486876000
1	-0.246552000	3.093383000	-2.911091000
1	0.747872000	1.749472000	-2.367857000

1	0.556799000	-3.874078000	-0.056048000
6	-1.458173000	-4.092699000	-0.399176000
6	0.710187000	-1.030561000	-1.730720000
1	-3.085640000	-1.707708000	5.031147000
1	-2.767640000	0.899020000	0.708283000
1	-1.551424000	-1.216016000	-0.891757000
8	1.607206000	-1.552823000	1.177533000
6	2.717021000	-2.144475000	0.627921000
1	3.120022000	-1.575899000	-0.235440000
1	2.508451000	-3.157374000	0.200077000
6	3.814387000	-2.296707000	1.664976000
1	3.467520000	-2.900077000	2.505652000
1	4.091911000	-1.318025000	2.059432000
1	4.708378000	-2.767150000	1.252287000
1	-2.354321000	-3.576248000	-0.052898000
1	-1.360911000	-3.866062000	-1.460481000
6	-1.563326000	-5.570748000	-0.161328000
6	-2.488637000	-6.089054000	0.740051000
6	-0.704090000	-6.452066000	-0.814250000
6	-2.556476000	-7.452496000	0.984800000
6	-0.766967000	-7.814304000	-0.572113000
6	-1.694144000	-8.317714000	0.329439000
1	-3.164404000	-5.410460000	1.250577000
1	0.015871000	-6.063512000	-1.526979000
1	-3.284045000	-7.839637000	1.686583000
1	-0.096043000	-8.486056000	-1.091791000
1	-1.746290000	-9.382262000	0.516862000

$TS3_{Bn}$ (Bn NH assisted amide formation)

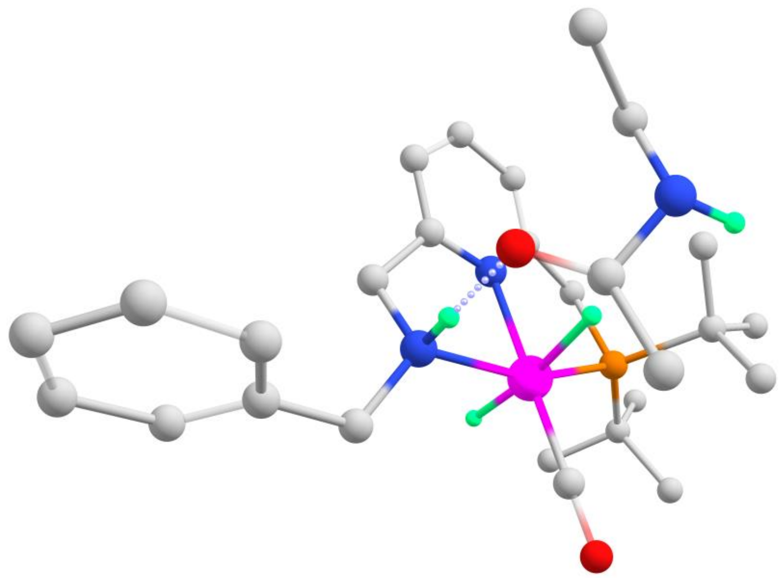

First three frequencies

	1	2	3
	A	A	A
Frequencies --	***-641.8570***	21.8936	27.5828
Red. masses --	***1.9232***	4.2675	3.9952
Frc consts --	***0.4668***	0.0012	0.0018
IR Inten --	***2430.3007***	0.4409	0.4119

Coordinates

44	-0.424481000	-0.878848000	0.113742000
15	-0.417190000	1.376118000	0.062093000
7	-0.873464000	-2.945828000	0.732121000
8	1.593139000	-1.191096000	-2.078437000
6	-3.335534000	-0.322572000	3.940698000
7	-1.889608000	-0.593497000	1.629994000
6	-1.911763000	1.753220000	1.103007000
1	-1.853710000	2.715864000	1.612960000
6	-2.247495000	0.637691000	2.040165000
6	-2.959665000	0.799666000	3.217367000
1	-3.219976000	1.793554000	3.555308000
6	-3.052007000	-1.583551000	3.443627000
1	-3.371388000	-2.477196000	3.961808000
6	-2.337554000	-1.690515000	2.261754000
6	-2.095202000	-2.980250000	1.547412000
1	-2.949558000	-3.146641000	0.879667000
1	-2.082105000	-3.817847000	2.251018000
6	0.971715000	2.250156000	1.016864000
6	2.303078000	1.666098000	0.548793000
1	3.118695000	2.086662000	1.141668000
1	2.514681000	1.876356000	-0.498582000
1	2.310001000	0.583161000	0.674400000
6	0.766002000	1.856237000	2.479078000
1	-0.057433000	2.398112000	2.945352000
1	1.666722000	2.086733000	3.053121000
1	0.571223000	0.788525000	2.573295000
6	1.008343000	3.770807000	0.938480000
1	1.255283000	4.143071000	-0.053730000
1	1.780339000	4.140513000	1.617710000
1	0.067572000	4.224965000	1.251814000
6	-0.768710000	2.328621000	-1.532725000
6	-1.755536000	1.503618000	-2.359207000
1	-1.331882000	0.548868000	-2.659566000
1	-2.019451000	2.066505000	-3.257209000
1	-2.678634000	1.291280000	-1.818385000
6	-1.389021000	3.708649000	-1.311579000
1	-2.339622000	3.652979000	-0.781106000
1	-1.599161000	4.159237000	-2.283887000
1	-0.743639000	4.396265000	-0.774697000
6	0.525888000	2.456286000	-2.334227000
1	0.299420000	2.857019000	-3.324282000
1	1.010593000	1.490623000	-2.478291000

1	-0.081546000	-3.110355000	1.407402000
6	0.816420000	-1.086417000	-1.219733000
1	-3.872201000	-0.212786000	4.873816000
1	-1.620492000	-0.913566000	-0.976541000
1	-2.743641000	1.831884000	0.395310000
8	1.004679000	-3.052425000	2.562670000
6	1.697947000	-2.008354000	2.283869000
1	0.909909000	-1.185228000	1.509808000
6	2.888369000	-2.196993000	1.356540000
1	3.628720000	-2.816343000	1.863053000
1	3.361043000	-1.257932000	1.066702000
1	2.565235000	-2.711282000	0.453668000
1	1.242811000	3.132005000	-1.871113000
7	2.032322000	-1.180059000	3.403420000
1	2.469006000	-0.317642000	3.108559000
6	0.963393000	-0.960070000	4.365251000
1	1.122684000	0.018017000	4.825668000
1	-0.005674000	-0.902863000	3.842563000
6	0.896086000	-2.029666000	5.433552000
1	1.826995000	-2.073404000	5.997736000
1	0.730155000	-3.002503000	4.977924000
1	0.084225000	-1.824628000	6.133152000
6	-0.846178000	-4.009164000	-0.288596000
1	-1.596890000	-3.781690000	-1.047749000
1	0.128048000	-3.933973000	-0.776794000
6	-2.051907000	-6.216896000	-0.249020000
6	-1.054388000	-5.393501000	0.260844000
6	-0.254346000	-5.866860000	1.301204000
1	0.514335000	-5.223006000	1.719333000
1	-2.680641000	-5.851559000	-1.053814000
1	-3.025250000	-8.124639000	-0.153352000
6	-2.246320000	-7.494378000	0.256732000
6	-1.446490000	-7.957990000	1.288916000
1	-1.596955000	-8.952792000	1.688319000
6	-0.453018000	-7.139658000	1.810243000
1	0.172804000	-7.496064000	2.618600000

4b (Et_2 alkoxy complex)

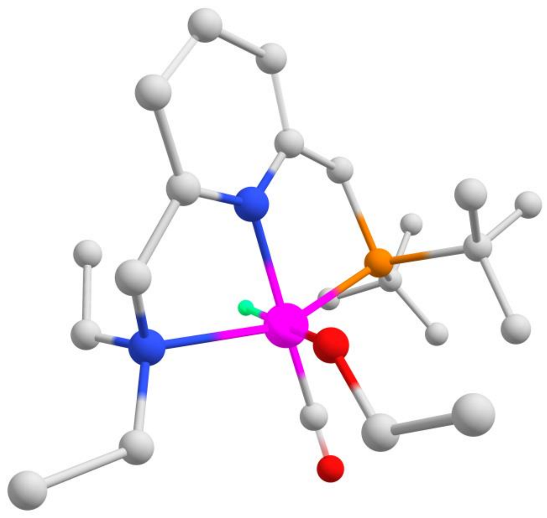

First three frequencies

	1	2	3
	**A**	A	A
Frequencies --	***38.8837***	52.0653	58.3056
Red. masses --	***3.7196***	3.6611	4.8652
Frc consts --	***0.0033***	0.0058	0.0097
IR Inten --	***0.2796***	0.0060	1.3507

Coordinates

44	-0.165135000	-1.234756000	-0.294029000
15	-0.386652000	0.949439000	0.097021000
7	-0.596193000	-3.522462000	-0.051183000
8	1.240227000	-0.881105000	-2.909289000
6	-2.421423000	-1.649748000	4.005258000
7	-1.239869000	-1.398209000	1.547089000
6	-1.820436000	0.919518000	1.284384000
1	-1.885678000	1.811086000	1.911259000
6	-1.828473000	-0.327527000	2.104768000
6	-2.424722000	-0.427087000	3.352673000
1	-2.885952000	0.443002000	3.799534000
6	-1.843698000	-2.749716000	3.396001000
1	-1.846552000	-3.720922000	3.872392000
6	-1.254966000	-2.592884000	2.150697000
6	-0.571000000	-3.702934000	1.417230000
1	-0.988101000	-4.671999000	1.716588000
1	0.484460000	-3.660134000	1.703598000
6	0.972318000	1.786911000	1.113233000
6	2.300872000	1.593514000	0.385526000
1	2.461347000	0.544289000	0.146750000
1	3.119396000	1.920685000	1.030717000
1	2.364981000	2.166937000	-0.537148000
6	1.034802000	1.011241000	2.433668000
1	0.164323000	1.202288000	3.063702000

1	1.909970000	1.347364000	2.994958000
1	1.140813000	-0.063540000	2.259904000
6	0.763969000	3.262693000	1.431398000
1	0.824144000	3.902158000	0.552266000
1	1.551289000	3.588083000	2.115747000
1	-0.187138000	3.453845000	1.930724000
6	-0.998375000	2.136874000	-1.242282000
6	-1.925535000	1.371652000	-2.185921000
1	-1.405113000	0.578153000	-2.715780000
1	-2.330313000	2.071579000	-2.920672000
1	-2.765794000	0.914548000	-1.662800000
6	-1.788926000	3.320948000	-0.681576000
1	-2.705474000	3.002730000	-0.185305000
1	-2.088302000	3.966761000	-1.509809000
1	-1.224125000	3.933246000	0.014571000
6	0.189997000	2.645542000	-2.057036000
1	0.818355000	3.334082000	-1.494957000
1	-0.179796000	3.186746000	-2.930338000
1	0.814695000	1.829639000	-2.420521000
6	-1.869095000	-3.976922000	-0.661247000
6	0.706783000	-1.034660000	-1.886360000
1	-2.880326000	-1.745966000	4.980667000
1	-2.713410000	0.914003000	0.650928000
1	-1.566314000	-1.084232000	-1.085646000
8	1.511676000	-1.803239000	1.008370000
6	2.830454000	-1.926735000	0.630232000
1	3.096678000	-1.287822000	-0.233634000
1	3.079008000	-2.953325000	0.278625000
6	3.768812000	-1.603659000	1.779936000
1	3.539758000	-2.231544000	2.642103000
1	3.654066000	-0.565258000	2.094131000
1	4.814430000	-1.763734000	1.509851000
1	-1.792698000	-3.731345000	-1.721415000
1	-1.930043000	-5.070005000	-0.587078000
6	-3.132750000	-3.383683000	-0.088319000
1	-3.120039000	-2.296186000	-0.138270000
1	-3.983150000	-3.738771000	-0.668476000
1	-3.304263000	-3.685709000	0.944784000
6	0.540115000	-4.247761000	-0.680485000
1	0.463550000	-4.060297000	-1.752629000
1	1.440582000	-3.748853000	-0.331369000
6	0.626978000	-5.736810000	-0.407330000
1	-0.237072000	-6.293353000	-0.767729000
1	0.748078000	-5.950960000	0.654291000
1	1.501387000	-6.141459000	-0.914001000

TS4$_{Et}$

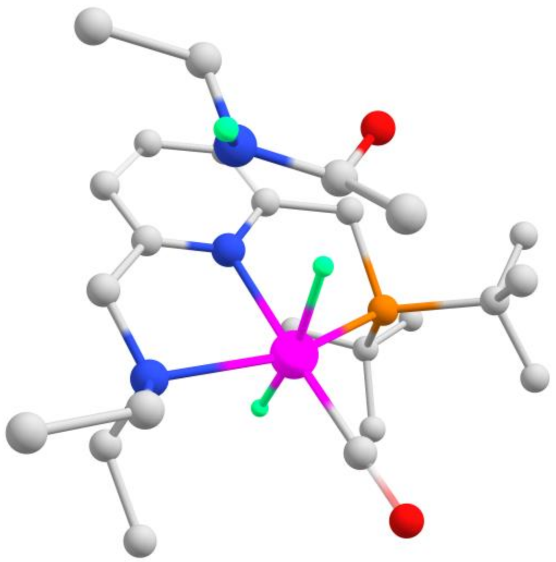

First three frequencies

	1	2	3
	A	A	A
Frequencies --	-537.7785	36.4810	49.7572
Red. masses --	2.1207	3.7039	4.8252
Frc consts --	0.3614	0.0029	0.0070
IR Inten --	2243.4448	0.7927	0.8197

Coordinates

44	-1.112085000	0.685691000	-0.412960000
1	-1.946743000	0.579836000	-1.798038000
15	-2.344326000	-1.050176000	0.313960000
8	-3.026101000	2.793519000	0.514414000
7	0.438041000	1.850157000	-1.689843000
7	0.225414000	-0.855786000	-1.021824000
6	-1.028528000	-2.345737000	0.393580000
6	0.021680000	-2.130168000	-0.643487000
6	0.818281000	-3.143579000	-1.159755000
1	0.648143000	-4.166256000	-0.852881000
6	1.822807000	-2.825139000	-2.057449000
1	2.437802000	-3.606625000	-2.484446000
6	2.058646000	-1.498297000	-2.380178000
1	2.869433000	-1.214903000	-3.037741000
6	1.253748000	-0.523277000	-1.814444000
6	1.585650000	0.933469000	-1.874646000
1	2.253442000	1.093526000	-1.020936000
1	2.140724000	1.169868000	-2.788937000
6	-2.274613000	1.980063000	0.155237000
6	-3.126868000	-1.092039000	2.031295000
6	-2.246005000	-0.229540000	2.933125000

1	-2.640306000	-0.269745000	3.951494000
1	-1.209810000	-0.573440000	2.951565000
1	-2.252967000	0.811419000	2.612899000
6	-4.520702000	-0.468675000	1.998415000
1	-4.868805000	-0.335231000	3.024648000
1	-4.512612000	0.516665000	1.531513000
1	-5.258176000	-1.084835000	1.487431000
6	-3.181780000	-2.495023000	2.635777000
1	-3.770593000	-3.201248000	2.056708000
1	-2.184946000	-2.911826000	2.774690000
1	-3.636812000	-2.431012000	3.626500000
6	-3.573394000	-1.757930000	-0.948808000
6	-4.566906000	-0.678451000	-1.375372000
1	-5.214227000	-0.356197000	-0.562985000
1	-4.050156000	0.197596000	-1.764142000
1	-5.208204000	-1.072144000	-2.167004000
6	-4.329437000	-2.987875000	-0.452668000
1	-4.918263000	-3.397799000	-1.276488000
1	-3.658494000	-3.779624000	-0.117043000
1	-5.023569000	-2.764971000	0.354132000
6	-2.769774000	-2.185075000	-2.180131000
1	-2.142577000	-1.378294000	-2.558521000
1	-2.140492000	-3.052875000	-1.979806000
1	-3.463590000	-2.469455000	-2.973786000
6	-0.111869000	2.184950000	-3.029917000
1	-0.439348000	1.239511000	-3.465086000
1	0.706387000	2.556628000	-3.661916000
6	-1.245107000	3.176546000	-3.018999000
1	-2.054108000	2.839830000	-2.374037000
1	-0.927063000	4.164978000	-2.689289000
1	-1.639982000	3.285925000	-4.027607000
6	0.870602000	3.056282000	-0.933917000
1	-0.024892000	3.637948000	-0.718389000
1	1.238310000	2.681614000	0.022457000
6	1.917513000	3.915067000	-1.612213000
1	2.193161000	4.740895000	-0.958724000
1	2.828857000	3.356629000	-1.827147000
1	1.560504000	4.348578000	-2.546274000
1	-1.403689000	-3.370412000	0.391067000
6	1.063726000	0.335509000	1.955537000
1	0.114399000	0.666156000	1.038847000
8	0.857467000	-0.845506000	2.356311000
6	0.822514000	1.505235000	2.908706000
1	1.535671000	1.441224000	3.734151000
1	-0.180121000	1.460129000	3.322147000
7	2.323250000	0.565087000	1.214981000
1	2.859192000	1.285470000	1.684568000
6	3.144280000	-0.624355000	1.040829000
1	3.368278000	-1.110565000	1.998298000
1	2.560836000	-1.362410000	0.487284000
6	4.421799000	-0.296694000	0.303320000

1	4.225584000	0.117551000	-0.687439000
1	5.020534000	0.436896000	0.846554000
1	-0.527072000	-2.159730000	1.355490000
1	5.039032000	-1.185252000	0.173418000
1	0.944470000	2.468395000	2.409769000

Ethylamine

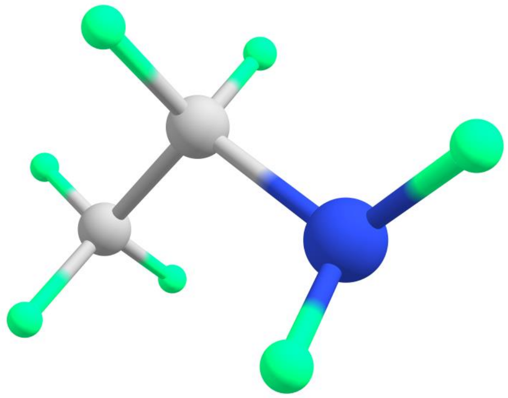

	1	2	3
	**A**	A	A
Frequencies --	***234.4040***	273.5739	412.0688
Red. masses --	***1.0655***	1.1239	2.2867
Frc consts --	***0.0345***	0.0496	0.2288
IR Inten --	***26.7733***	12.9756	9.0332

Coordinates

6	1.061228000	0.413189000	-0.106269000
6	-0.100317000	-0.546246000	0.001396000
7	-1.363388000	0.165205000	-0.162536000
1	1.039455000	0.941885000	-1.058166000
1	1.025393000	1.162895000	0.685910000
1	-0.025950000	-1.104551000	0.946520000
1	-0.036090000	-1.292390000	-0.793969000
1	-1.473335000	0.839912000	0.585025000
1	-2.141988000	-0.474746000	-0.076101000
1	2.014990000	-0.105154000	-0.021811000

Hydrogen

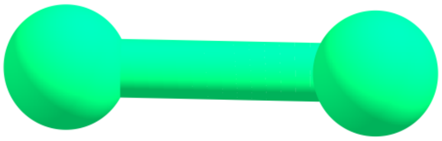

Frequency

	1
	**A**
Frequencies --	***4342.3709***
Red. masses --	***1.0078***
Frc consts --	***11.1967***

```
 IR Inten    --       0.0000
```

Coordinates

1	-3.519951000	0.215525000	0.000000000
1	-2.775939000	0.206181000	0.000000000

Acetaldehyde

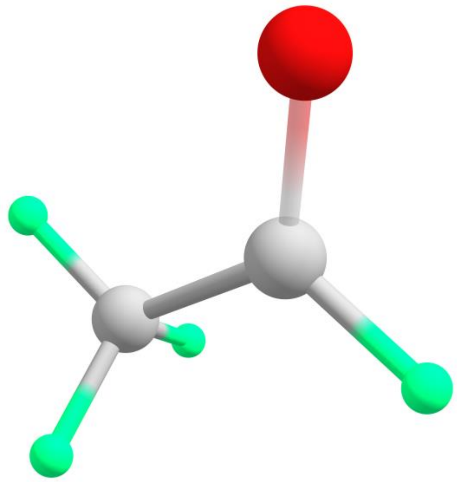

```
                      1                      2                      3
                      A                      A                      A
 Frequencies --    154.3049               506.9440               767.3056
 Red. masses --      1.2367                 2.4776                 1.1213
 Frc consts  --      0.0173                 0.3752                 0.3890
 IR Inten    --      0.4429                11.7396                 0.3559
```

Coordinates

6	3.494242000	0.601354000	-0.016792000
6	4.223765000	-0.700487000	0.003778000
1	2.547466000	0.518307000	-0.542768000
1	4.115023000	1.363965000	-0.489731000
1	3.321817000	0.943236000	1.004921000
8	3.829055000	-1.719751000	-0.492568000
1	5.206026000	-0.671724000	0.533159000

10. References

1. Fogler, E.; Garg, J. A.; Hu, P.; Leitus, G.; Shimon, L. J. W.; Milstein, D., System with Potential Dual Modes of Metal–Ligand Cooperation: Highly Catalytically Active Pyridine-Based PNNH–Ru Pincer Complexes. *Chem. - Eur. J.* **2014,** *20*, 15727-15731.
2. Kumar, A.; Janes, T.; Espinosa-Jalapa, N. A.; Milstein, D., Selective Hydrogenation of Cyclic Imides to Diols and Amines and Its Application in the Development of a Liquid Organic Hydrogen Carrier. *J Am Chem Soc* **2018,** *140*, 7453-7457.
3. Gunanathan, C.; Ben-David, Y.; Milstein, D., Direct Synthesis of Amides from Alcohols and Amines with Liberation of H_2. *Science* **2007,** *317*, 790.
4. Balaraman, E.; Gnanaprakasam, B.; Shimon, L. J. W.; Milstein, D., Direct Hydrogenation of Amides to Alcohols and Amines under Mild Conditions. *J. Am. Chem. Soc.* **2010,** *132*, 16756-16758.
5. Srimani, D.; Balaraman, E.; Hu, P.; Ben-David, Y.; Milstein, D., Formation of Tertiary Amides and Dihydrogen by Dehydrogenative Coupling of Primary Alcohols with Secondary Amines Catalyzed by Ruthenium Bipyridine-Based Pincer Complexes. *Adv. Synth. Catal.* **2013,** *355*, 2525-2530.
6. Espinosa-Jalapa, N. A.; Nerush, A.; Shimon, L. J. W.; Leitus, G.; Avram, L.; Ben-David, Y.; Milstein, D., Manganese-Catalyzed Hydrogenation of Esters to Alcohols. *Chem. - Eur. J.* **2017,** *23*, 5934-5938.
7. Das, U. K.; Ben-David, Y.; Diskin-Posner, Y.; Milstein, D., N-Substituted Hydrazones by Manganese-Catalyzed Coupling of Alcohols with Hydrazine: Borrowing Hydrogen and Acceptorless Dehydrogenation in One System. *Angew. Chem. Int. Ed.* **2018,** *57*, 2179-2182.
8. Sheldrick, G., SHELXT - Integrated space-group and crystal-structure determination. *Acta Crystallogr. A* **2015,** *71*, 3-8.
9. Sheldrick, G., Crystal structure refinement with SHELXL. *Acta Crystallogr. C* **2015,** *71*, 3-8.
10. Dolomanov, O. V.; Bourhis, L. J.; Gildea, R. J.; Howard, J. A. K.; Puschmann, H., OLEX2: a complete structure solution, refinement and analysis program. *J. Appl. Crystallogr.* **2009,** *42*, 339-341.
11. Satoh, T.; Motohashi, S.; Kimura, S.; Tokutake, N.; Yamakawa, K., The asymmetric favorskii rearrangement: A synthesis of optically active α-alkyl amides from aldehydes and (−)-1-chloroalkyl p-tolyl sulfoxide. *Tetrahedron Lett.* **1993,** *34*, 4823-4826.
12. Foubelo, F.; Yus, M., EPC-Synthesis of functionalised amides via chiral β-nitrogenated organolithium compounds. *Tetrahedron: Asymmetry* **1996,** *7*, 2911-2922.
13. Zhao, Y.; Truhlar, D. G., A new local density functional for main-group thermochemistry, transition metal bonding, thermochemical kinetics, and noncovalent interactions. *J. Chem. Phys.* **2006,** *125*, 194101.
14. Weigend, F.; Ahlrichs, R., Balanced basis sets of split valence, triple zeta valence and quadruple zeta valence quality for H to Rn: Design and assessment of accuracy. *Phys. Chem. Chem. Phys.* **2005,** *7*, 3297-3305.
15. Weigend, F., Accurate Coulomb-fitting basis sets for H to Rn. *Phys. Chem. Chem. Phys.* **2006,** *8*, 1057-1065.
16. Grimme, S.; Antony, J.; Ehrlich, S.; Krieg, H., A consistent and accurate ab initio parametrization of density functional dispersion correction (DFT-D) for the 94 elements H-Pu. *J. Chem. Phys.* **2010,** *132*, 154104.

17. Mardirossian, N.; Head-Gordon, M., ωB97M-V: A combinatorially optimized, range-separated hybrid, meta-GGA density functional with VV10 nonlocal correlation. *J. Chem. Phys.* **2016,** *144*, 214110.
18. (a) Vydrov, O. A.; Van Voorhis, T., Nonlocal van der Waals density functional: The simpler the better. *J. Chem. Phys.* **2010,** *133*, 244103; (b) Hujo, W.; Grimme, S., Performance of the van der Waals Density Functional VV10 and (hybrid)GGA Variants for Thermochemistry and Noncovalent Interactions. *J. CHem. Theory Comput.* **2011,** *7*, 3866-3871.
19. Hellweg, A.; Hättig, C.; Höfener, S.; Klopper, W., Optimized accurate auxiliary basis sets for RI-MP2 and RI-CC2 calculations for the atoms Rb to Rn. *Theor. Chem. Acc.* **2007,** *117*, 587-597.
20. Frisch, M. J.; Trucks, G. W.; Schlegel, H. B.; Scuseria, G. E.; Robb, M. A.; Cheeseman, J. R.; Scalmani, G.; Barone, V.; Petersson, G. A.; Nakatsuji, H.; Li, X.; Caricato, M.; Marenich, A. V.; Bloino, J.; Janesko, B. G.; Gomperts, R.; Mennucci, B.; Hratchian, H. P.; Ortiz, J. V.; Izmaylov, A. F.; Sonnenberg, J. L.; Williams; Ding, F.; Lipparini, F.; Egidi, F.; Goings, J.; Peng, B.; Petrone, A.; Henderson, T.; Ranasinghe, D.; Zakrzewski, V. G.; Gao, J.; Rega, N.; Zheng, G.; Liang, W.; Hada, M.; Ehara, M.; Toyota, K.; Fukuda, R.; Hasegawa, J.; Ishida, M.; Nakajima, T.; Honda, Y.; Kitao, O.; Nakai, H.; Vreven, T.; Throssell, K.; Montgomery Jr., J. A.; Peralta, J. E.; Ogliaro, F.; Bearpark, M. J.; Heyd, J. J.; Brothers, E. N.; Kudin, K. N.; Staroverov, V. N.; Keith, T. A.; Kobayashi, R.; Normand, J.; Raghavachari, K.; Rendell, A. P.; Burant, J. C.; Iyengar, S. S.; Tomasi, J.; Cossi, M.; Millam, J. M.; Klene, M.; Adamo, C.; Cammi, R.; Ochterski, J. W.; Martin, R. L.; Morokuma, K.; Farkas, O.; Foresman, J. B.; Fox, D. J. *Gaussian 16 Rev. C.01*, Wallingford, CT, 2016.
21. Neese, F., Software update: the ORCA program system, version 4.0. *WIREs Comput. Mol. Sci.* **2018,** *8*, e1327.
